# Mechanism underlying the high regulatory performance of the doxycycline riboswitch G12

**DOI:** 10.1038/s41467-026-76256-2

**Published:** 2026-08-03

**Authors:** J. Hoetzel, A. Walbrun, M. Schäfer, T. Wang, A. G. Jørgensen, O. Becker, K. Stamatakis, V. Gunawan, L. Reichardt, L. Boettger, R. W. Bruckhoff, J. Kjems, J. Wachtveitl, M. Rief, B. Suess

**Affiliations:** 1 Department of Biology, Technical University of Darmstadt, Darmstadt, Germany; 2 TUM School of Natural Sciences, Department of Bioscience, Center for Functional Protein Assemblies (CPA), Technical University of Munich, Garching, Germany; 3 Department of Molecular Biology and Genetics, Interdisciplinary Nanoscience Center, Aarhus University, Aarhus, Denmark; 4 Institute for Physical and Theoretical Chemistry, Goethe-University at Frankfurt, Frankfurt am Main, Germany; 5 Centre for Synthetic Biology, Technical University of Darmstadt, Darmstadt, Germany

**Keywords:** Riboswitches, RNA, Molecular biophysics

## Abstract

Synthetic riboswitches provide protein-independent, modular control of gene expression, yet selecting aptamers that reliably couple ligand binding to regulatory switching remains challenging. Here, we identify and mechanistically characterise G12, a doxycycline-binding aptamer with remarkably high regulatory performance in yeast and human cells. We provide evidence that RNA Capture-SELEX efficiently enriches aptamers with ligand-responsive conformational switching. We compared conventional SELEX and RNA Capture-SELEX using the same starting library followed by NGS analysis and in vivo screening, which led to the identification of G12. G12 binds doxycycline with low-nanomolar affinity and strict discrimination against close derivatives, thus enabling high-dynamic-range riboswitch control of translation in yeast and splicing in human cells. Single-molecule force spectroscopy with optical tweezers revealed that doxycycline stabilises a folding intermediate independent of the closing stem P1, which primarily acts as a scaffold for correct aptamer folding. Mutational analysis and chemical probing identified tertiary contacts between loops L2 and L3 in this intermediate state. Stopped-flow fluorescence spectroscopy further supported a two-step binding mechanism consistent with efficient regulatory switching. Together, these findings deepen our understanding of regulatory aptamer selection and function and expand the synthetic biology toolbox with a high-performance doxycycline-responsive riboswitch.

## Abstract (web summary)

Synthetic riboswitches provide protein-independent, modular control of gene expression, yet selecting aptamers that reliably couple ligand binding to regulatory switching remains challenging. In this study, the authors identified a regulatory RNA aptamer binding doxycycline for the engineering of synthetic riboswitches.

## Introduction

Efficient and precise gene regulation is a fundamental prerequisite for many areas of biological and medical research. Conditional gene expression has enabled in-depth studies of brain development, oncogenesis, and viral infection^1–3^. Commonly used approaches include the TetON-system, which employs different variants of the bacterial Tet-repressor, or the CRISPR-Cas system, which uses Cas-family proteins to control gene expression at the DNA or RNA level^4–7^. Despite their strong performance, these systems are suboptimal for applications requiring protein independence or precise regulation of individual transcripts^8^. Riboswitches offer a compact, versatile and protein-independent alternative for gene regulation and beyond^9^. Naturally found mainly in the 5’UTR of many bacteria, they exert control over transcription or translation of the mRNA they are located on^10–12^. In most cases, these riboswitches are responsive to metabolites functionally connected to the protein encoded by the controlled mRNA^13–15^. Although riboswitches are most prominent in bacteria, additional examples have been identified in the mRNA of fungi, archaea and plants, resulting in more than 55 classes of natural riboswitches to date^16–18^.

Meanwhile, this elegant concept of gene regulation has been adopted for the engineering of synthetic riboswitches. These are composed of an aptamer domain that senses a specific ligand and a regulatory domain that mediates gene regulation^19^. Owing to their simple yet effective architecture, synthetic riboswitches have been successfully applied beyond their bacterial origin for regulatory purposes across all domains of life^8^. Like their natural counterparts, synthetic riboswitches operate through structural changes that are induced by the binding of their ligand to the aptamer domain^20–23^. These changes, in turn, are transmitted to a regulatory domain and ultimately lead to the regulation of gene expression. Due to their modular nature, synthetic riboswitches have been engineered to operate in a vast number of different functional mechanisms. Depending on the regulatory domain used, this allows for different modes of gene regulation in a wide range of different species, including control of alternative splicing in human cells^24^, blocking of ribosomal scanning in yeast^25^, control of RBS accessibility in bacteria and archaea^26,27^, control of ribozyme cleavage in *Caenorhabditis elegans*^28^ or of polyadenylation in mice^29^. Despite their differences, all of the synthetic riboswitches above share the characteristic of utilising an aptamer as their sensor domain, selected in vitro through Systematic Evolution of Ligands by EXponential enrichment (SELEX)^30,31^. Developed for the purpose of selecting nucleic acid binding motifs de novo, SELEX opens the possibility to identify aptamers binding to virtually any given ligand. This creates the opportunity to engineer riboswitches using the same regulatory domain but aptamers responsive to different ligands^32^. Such modularity enables orthogonal gene regulation that allows independent control of multiple riboswitches in the same cell. The versatility of synthetic riboswitches has also led to applications beyond mere gene expression regulation like the intracellular detection of metabolites by fluorogenic readout or in vitro biosensing^33–35^. Due to their compact format and RNA only-nature, synthetic riboswitches hold great potential for the control of RNA therapeutics, therapeutic cells and viral vectors.

Regardless of the final application of a riboswitch, the key element determining functionality is the aptamer domain. Although several hundred RNA aptamers have been selected since the invention of SELEX, only a handful meet the criteria for the engineering of riboswitches. Structural changes within the riboswitch are fundamental to the regulatory response. Consequently, only aptamers in which ligand binding induces conformational changes can be used for riboswitch engineering^36^. These structural changes are accompanied by a high affinity for the ligand^37–39^ and a distinct two-step binding mechanism, observed in regulatory aptamers like the tetracycline, neomycin or ciprofloxacin-binding aptamers^40–42^. To facilitate the selection of novel aptamers that meet these criteria, two main factors in the selection have been adjusted: the SELEX method itself and subsequent screenings to identify suitable candidates. With the selection step being a decisive factor for the performance of the selected aptamers, SELEX methods like Capture-SELEX, in vivo SELEX or ultraSELEX have been developed to increase the selection efficiency of aptamers fit for their intended application^43–45^. Among these specialised SELEX methods, RNA Capture-SELEX was developed for the effective selection of RNA aptamers undergoing conformational changes upon ligand binding^46^. In contrast to conventional SELEX methods, the RNA pool is coupled to beads through hybridisation to an immobilised DNA oligonucleotide while the ligand is free in solution (conventional SELEX methods require immobilisation of the ligand). In this setup, candidates that undergo structural changes upon ligand binding detach from the DNA oligonucleotide and are subsequently selected. Previous selection attempts have shown that a follow-up screening is crucial for the effective identification of suitable candidates^38^. When selecting for regulatory aptamers, in vivo screening in *Saccharomyces cerevisiae* has proven to be an efficient method to identify aptamer candidates with favourable characteristics^38,39^.

Despite progress in riboswitch engineering, the details of the structural mechanisms governing aptamer function remain poorly understood. Aptamers often adopt structures with complex folding landscapes (including tertiary interactions), and ligand binding typically induces substantial structural rearrangements, rendering the RNA highly dynamic^47^. Moreover, ligand binding in regulatory aptamers itself is inherently dynamic and complex, occurring via a characteristic two-step binding mechanism^36,40^. Traditional structural biology approaches face limitations in this context: RNA structures are often too large or heterogeneous for high-resolution NMR, yet too flexible for Cryo-EM. As a result, single-molecule mechanical experiments have emerged as powerful tools to probe RNA structure and dynamics at high resolution^48,49^. In particular, single-molecule force spectroscopy (SMFS) using optical tweezers has provided mechanistic insights into naturally occurring riboswitches, including the *pbuE* and *add* adenine riboswitches^50,51^ and the thiamine pyrophosphate riboswitch^52^. These studies revealed details that are difficult to obtain through ensemble approaches. Despite these successes, optical tweezers remain largely unexplored for synthetic riboswitches.

In this work, we explore whether RNA Capture-SELEX is particularly well-suited for the selection of regulatory RNA aptamers. We performed a parallel in vitro selection for doxycycline binding aptamers using Capture-SELEX and conventional SELEX for a direct comparison of the two methods. We identify and characterise an aptamer with remarkably high regulatory performance. Using optical-tweezers–based SMFS and stopped-flow spectroscopy, we provide a detailed analysis of the doxycycline aptamer, revealing a unique binding mechanism and previously unreported structural behaviour. Together, these results offer important insights into the characteristics of regulatory aptamers and provide a mechanistic explanation for the high regulatory performance of the aptamer G12.

## Results

### Discovery of doxycycline-binding aptamers with regulatory potential using two in vitro selection methods and subsequent in vivo screening

**Fig. 1:**
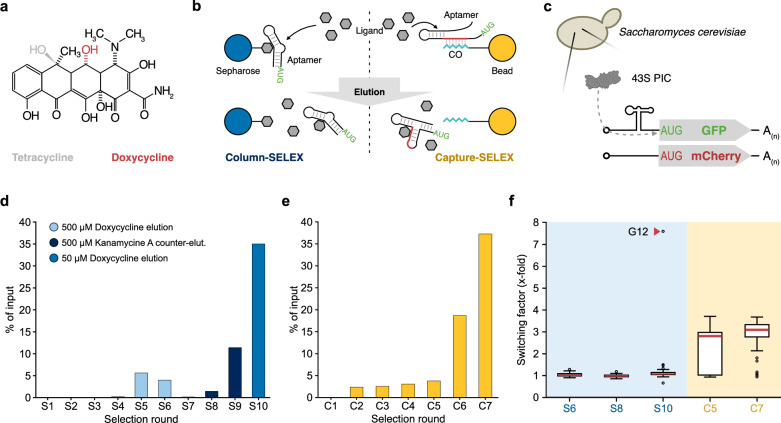
In vitro selection and subsequent in vivo screening of doxycycline-binding aptamers. **a** Natta projection of tetracycline and doxycycline (they only differ in the position of a single hydroxyl group). **b** Schematic of the selection step in column- and Capture-SELEX. Column SELEX: the ligand (grey hexagon) is immobilised on column material (blue). The elution of bound RNA sequences is performed by washing with high concentration of the ligand. Capture-SELEX: the RNA pool is immobilised by hybridisation of a docking sequence (red) to the capture-oligonucleotide (turquoise) that is coupled to magnetic beads (yellow). RNA that undergoes conformational changes upon ligand binding detaches from the capture-oligonucleotide and subsequently elutes. The Capture-SELEX pool, that was used for both selections, contains a start codon (green AUG) in its 3’ constant region, enabling to use selected sequences directly for translation control in yeast. **c** Schematic of the roadblock mechanism in S. cerevisiae. Aptamer candidates are inserted in the 5’UTR of GFP, mCherry mRNA without aptamer sequence serves for normalisation. Stabilisation of the aptamer upon binding of doxycycline can block the ribosomal 43S pre-initiation complex (43S PIC) from reaching the start codon and repress translation. **d** Progress of column-SELEX expressed as eluted RNA fraction in percent relative to the input. The different selection parameters over the course of selection are indicated by colour: elution with 500 µM doxycycline (light blue), additional counter-selection with 500 µM kanamycin A (dark blue) and elution with reduce doxycycline concentration of 50 µM (navy blue). Counter-selection was introduced to remove weak binders after observing signs of increased unspecific retention in the column. **e** Progress of Capture-SELEX expressed as eluted RNA fraction in percent relative to the input. **f** In vivo screening in S. cerevisiae for the pools obtained from column-SELEX (blue background) and Capture-SELEX (yellow background). Switching behaviour of different selection rounds are displayed. (*n* = 94, red line = median, box = first & third quartiles, whiskers = minimum / maximum, circles = outliers). The aptamer G12 is highlighted by a red arrowhead in the pool S10.

We started this project with the aim of identifying a regulatory doxycycline aptamer. This occasion was used to test our hypothesis that RNA Capture-SELEX is particularly effective for selecting aptamers undergoing conformational changes upon ligand binding. For both SELEX experiments we started with the identical library to directly compare their outcomes (Fig. 1a, b, and Figure S1a). Beginning with the conventional SELEX (column-based, column-SELEX), we obtained the first enrichment after five rounds (Fig. 1d, and Table S1). Increase in washing steps and counter-selection led to a decrease in eluted RNA, which recovered in round S9 and even further increased in round S10 with reduced doxycycline concentration. Capture-SELEX was performed starting with the identical library used for column-SELEX^46^. After six rounds of selection an increased elution of RNA was observed, which further increased in round C7 (Fig. 1e, and Table S2).

The enriched pools of round S6, S8 and S10 (column-SELEX) and round C5 and C7 (Capture-SELEX) were used for in vivo screening in *S. cerevisiae*^53^. The pools were cloned into the 5’UTR of a GFP gene where the aptamers can act as a roadblock to the ribosomal pre-initiation complex and thereby control translation initiation (Fig. 1c, and Figure S1b)^25^. mCherry was constitutively expressed from the same plasmid for normalisation. First, we pre-sorted the sequences for sufficient GFP expression in the absence of doxycycline, to remove candidates that inhibit expression independent of the ligand (Figure S2, and Table S3). With progressing selection rounds from the column-SELEX, we observed a reduction in the preselected fraction (Figure S1c, and S3a–f), in contrast to Capture-SELEX (Figure S1d, and S3g–l). This implies a difference in enriched sequences between the two SELEX methods. Among the pre-selected candidates from the column-SELEX, only a single aptamer showed notable switching with a 7-fold dynamic range (G12, Fig. 1f), whereas Capture-SELEX pools displayed a median dynamic range of 3-fold (Fig. 1f). This revealed a high fraction of switchable sequences and a striking difference in the enrichment of conformationally responsive aptamers. Sequencing of regulatory candidates identified one aptamer from column-SELEX, G12, and one major contributor to switching in the Capture-SELEX pool, C01 (both SELEX methods are compared in detail in the Supplementary Information Box).

### Next-generation sequencing reveals differences in selection dynamics between SELEX methods

The marked difference in enriched regulatory aptamers between the two SELEX methods raised the question of how selection dynamics shaped sequence composition. As SELEX mechanisms strongly influence sequence characteristics and regulatory potential^36^, all rounds were analysed by next-generation sequencing. From these data, the ten most abundant 6–12 nt motifs per round were identified by *Multiple EM for Motif Elicitation* (MEME)^54^, mapped by *Find Individual Motif Occurrences* (FIMO) for similarity^55^, and grouped into motif clades (Figure S4a–S4d). The resulting motif clades were then tracked in their abundance over the course of their respective SELEX.

**Fig. 2:**
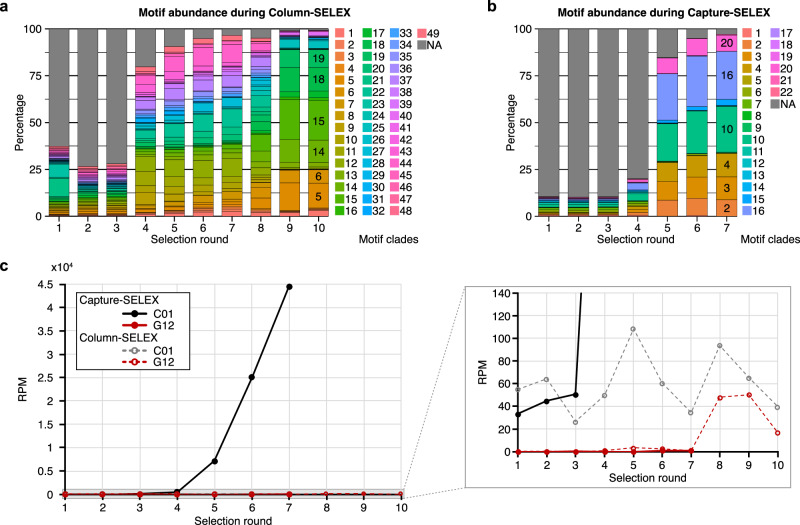
NGS analysis of selection dynamics. **a** Tracking of the most abundant motif clades over the course of column-SELEX selection. The different motif clades are indicated by colour and then plotted by their relative abundance in percent over all analysed reads. Dominant clades in the last selection rounds are indicated by their clade number. **b** Tracking of the most abundant motif clades over the course of Capture-SELEX selection. **c** Tracking of the two aptamer candidates G12 (red) and C01 (black) over the course of Capture-SELEX (solid line) and Column-SELEX (dashed line). The abundance of each candidate is indicated for each round of selection (*x*-axis) in reads per million (RPM, *y*-axis). For better visualisation of the selection progress of candidates, a zoom-in of the main figure is provided on the right to resolve the dynamics between 0 and 140 RPM.

In the column-SELEX, we found 49 motif clades for which abundance changed with selection parameters (Fig. 2a). Counter-selection notably altered clade composition, whereas reduced doxycycline elution in round S10 had little effect. Capture-SELEX, in contrast, yielded 22 motif clades, with a rapid enrichment of the few dominant ones within seven rounds (Fig. 2b). Within the first five rounds of selection, the diversity of motif clades was quickly reduced to seven with considerable abundance. Between selection round C5 and C7, six motif clades (clade 2, 3, 4, 10, 16 & 20) accumulated in a dominant way. Checking the origin revealed that three of these motif clades can be attributed to parts of the aptamer C01 (Figure S4e). In comparison, none of the dominant motif clades from column-SELEX could be attributed to G12 or C01.

To understand the enrichment of the two aptamers, we tracked their abundance in both selections. In the column-SELEX, C01 fluctuated, starting in the Top25 in early rounds, then dropping out of the Top100 while still increasing in read count (Fig. 2c). G12 started very low and did not increase in abundance until round S8. Both aptamers decreased in round S10 with reduced doxycycline in elution. In Capture-SELEX, C01 appeared after round C1 in the Top10, became dominant from round C4 onward, and enriched continuously to round C7 (Fig. 2c). In contrast, G12 remained very low, never reaching the Top100 and showing only slight enrichment in the last three rounds. Looking for an explanation, we tested G12 hybridisation to the capture-oligonucleotide and found it could not effectively hybridise (Figure S1e). Overall, the observed differences can be attributed to the differences between the selection methods (see Supplementary Information Box).

### Characterisation and optimisation of the doxycycline-binding aptamer G12

**Fig. 3:**
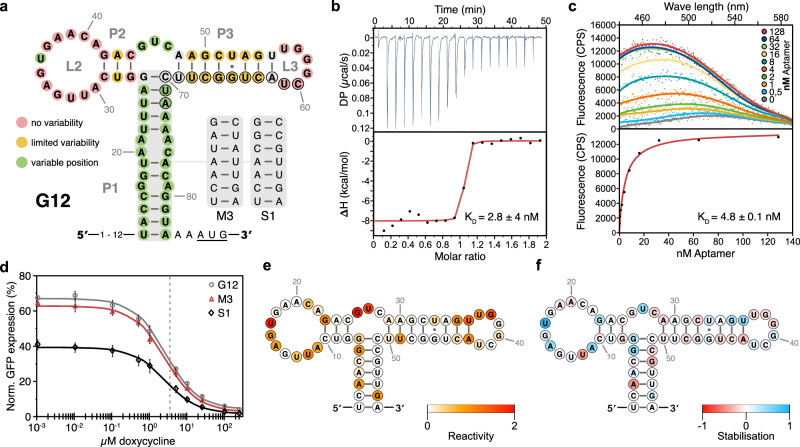
Biochemical characterisation of the doxycycline-binding aptamer G12. **a** Folding prediction of G12 as found in the in vivo screening. The colour coding represents the summary of the findings from the rational and randomised mutational studies, with red indicating positions with no mutation tolerance, yellow indicating limited mutation tolerance and green indicating positions that allows free nucleotide variability. Alternative P1 stems as used in the variants M3 and S1 are shown in the bottom right corner. Nucleotide positions in the P3 region with a black circle represent the docking sequence as found in the Capture-SELEX pool. **b** Representative ITC results of G12 showing the titration curve and thermogram. **c** Representative FTS results of G12 showing the fluorescence curves of different aptamer concentrations and the curve fit over the experimental data. (*n* = 2) **d** Dose-response curve of G12, M3 and S1 in yeast regulating GFP expression via the roadblock mechanism. Expression levels are shown over varying doxycycline concentrations on the *x*-axis, expressed as percent normalised and relative to a consecutive GFP expressing positive control (pCBB05) indicated on the *y*-axis. Experimental data are represented by the data points (as mean +/- SEM of *n* = 6), and the fitted curve is shown in corresponding colour. The EC_50_ of all three constructs was close to 3 µM as indicated by the grey, dashed line. **e** Chemical probing of S1 by SHAPE-MaP in the absence of doxycycline. Reactivity of each nucleotide position is indicated by colour (red = highest reactivity) in the 2D representation of the aptamer. **f** Stabilisation of nucleotide positions as calculated from SHAPE-MaP results of S1 with and without doxycycline and mapped in colour to the 2D representation of the aptamer. Intensity of colour (blue) indicates reduced reactivity of a nucleotide position, implying increased position, stability upon binding of doxycycline.

We decided to continue characterisation of G12 due to its remarkable regulatory behaviour (Fig. 1f). The folding prediction of G12 proposed a T-shaped secondary structure with a closing stem P1, transitioning into the J1-3 three-way junction^56^. Helices P2 and P3 were expected to end in a 12 and 6 nucleotide long loop, respectively (Fig. 3a). We validated this prediction by mutational studies and chemical probing (Figure S5–S8, Table S4). Stabilising P1 by creating perfect base pairing (M1, M2) retained regulatory activity but reduced expression (Figure S5a, S5d, S6). Truncating P1 to seven base pairs (M3) increased dynamic range without lowering expression in the absence of doxycycline. We therefore proceeded with M3. Replacing the L3 loop sequence (M4, M5) completely eliminated regulation. Disrupting the helix P3 (M9) likewise abolished regulation, whereas restoring base pairing (M10) recovered activity. Flipping the P3 stem sequence (M11) performed comparably to M3 (Figure S5b, S5e, S6). Inserting one base pair (M12) reduced, and inserting two (M13) fully eliminated regulation, indicating a structural role for P3 largely independent of sequence (Figure S5b, S5e, S6). Exchanging the three nucleotides in the J1-3 junction (M14) resulted in a behaviour similar to M3, suggesting this sequence as not critical (Figure S5b, S5e, S6). In contrast, altering P2 (M15, M16, M18, M19) strongly reduced or eliminated regulation, with the only exception of flipping the middle A–U base pair (M17), which maintained M3-like behaviour (Figure S5b, S5e, S5f, S6). Any exchange in L2 (triplet-wise mutation to CAA, M20–M23) caused complete loss of regulatory activity (Figure S5b, S5f, S6).

We then performed a random mutagenesis at the single nucleotide level (G7 to C51) in the M3 aptamer. We screened the variants for regulatory activity and analysed the outcome by NGS (Figure S5c, S7, Table S5). Most single mutations caused loss of function (Figure S5c). Only five positions tolerated changes: U17 in L2; G26, U27, C28 in J1-3; and substitution of G46 in P3 with adenosine. This confirms sequence sensitivity in L2/L3 and supports a scaffolding role for stems P1–P3 (Fig. 3a). Because P1 composition strongly affects riboswitch performance^25,57^, we created stem variants of M3 with different stabilities (S1–S5, Figure S5b), resulting in S1 as the best performing candidate (Figure S5g). SHAPE-MaP^58,59^ of G12_S1 with and without doxycycline showed unexpected low overall reactivity, suggesting a pre-structured RNA folding (Fig. 3e, Figure S8a, S8b). In line with our mutational studies, J1-3 was flexible. Additional flexibility occurred in L2 and L3, although several nucleotides showed low reactivity, which may indicate tertiary interactions. High reactivity at U17, G26 and U27 corresponded well with the mutational screening (Figure S5c). Binding to doxycycline stabilised large parts of the aptamer (Fig. 3f, Figure S8c–f), especially within L2 and L3, as well as the upper region of P1.

Finally, we determined the K_D_ of the aptamer variants G12, M3 and S1 by isothermal titration calorimetry (ITC) and fluorescence titration spectroscopy (FTS) and found similar affinities ( ≈ 4 nM, Figs. 3b, c, Figure S9a–i), indicating improved regulation was not due to stronger binding. Furthermore, we determined a comparable EC_50_ for all three variants (2.7 µM) in yeast, thus confirming the scaffolding role of the P1 stem with influence on overall expression but not on the efficiency of regulation (Fig. 3d, Table S6). When analysing the binding of selected mutants (M5, M9, M10, M14, M22) to doxycycline, all mutations with a loss of gene regulation in yeast showed no binding to doxycycline (Figure S10). Testing the binding of G12 to five close derivatives of doxycycline revealed no signs of binding, highlighting the high specificity of the RNA aptamer towards its cognate ligand (Figure S11a–S11f, S12).

### Application in vivo reveals exceptional regulatory potential of G12 in yeast and human cells

**Fig. 4:**
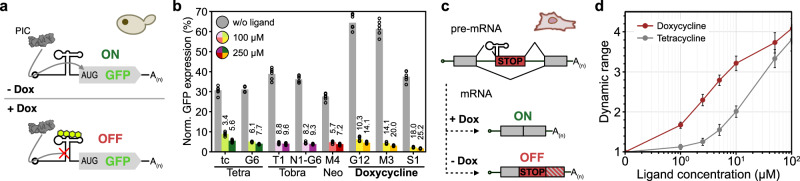
Characterisation of in vivo functionality of G12. **a** Schematic of translation initiation control in yeast through the roadblock mechanism. Translation of the reporter gene (GFP) is controlled by the stability of the aptamer placed in the 5’UTR of the gene. In absence of its ligand, the aptamer is in a relatively loose state, allowing the ribosomal pre-initiation complex (PIC) to pass. Upon binding its ligand, the aptamer stabilises and blocks the PIC from passing, ultimately supressing translation by keeping the PIC from reaching the start codon. **b** Comparative measurement of different regulatory aptamers regulating translation via the roadblock mechanism. Expression levels are shown in percent normalised and relative to the positive control. Grey bars show the expression level of the constructs in the absence of their ligand; light coloured bars show the expression level in the presence of 100 µM ligand concentration and darker coloured bars show the expression levels at 250 µM ligand concentration. The fold changes in reporter expression levels are given above the respective bars. Shown are the mean values of two independent measurements with 3 biological replicates (*n* = 6). **c** Schematic of the mechanism controlling mRNA processing through 3’ splice site accessibility. The synthetic exon (red) containing premature stop codons is spliced into the mRNA in absence of doxycycline, due to the accessibility of the splice site to the spliceosome, leading to premature termination of translation. In presence of doxycycline, the splice site is masked and the synthetic exon spliced out from the mRNA. **d** Dynamic range of reporter gene expression at different concentrations of respective ligands of the doxycycline-binding riboswitch (red) and the tetracycline-binding riboswitch (grey) in HeLa cells. The riboswitch construct containing G12 shows an earlier response at lower concentrations of its ligand and an overall higher dynamic range when compared with the tetracycline riboswitch. Data points represent the mean with error bars representing +/- SEM as calculated from two independent measurements in triplicates (*n* = 6).

The assessment of the regulation of the aptamer G12 side-by-side with previously described synthetic riboswitches revealed its enhanced regulatory capability. In a comparative measurement of controlling translation initiation in yeast, G12 and its variants M3 and S1 were compared with the tetracycline^37^, the neomycin^60^ and the tobramycin aptamers^39^. All three variants of the doxycycline aptamer showed the highest dynamic range (Fig. 4a, b, and Table S7). The improved regulatory performance largely reflects high expression in the absence of ligand, while inhibition in the presence of ligand remains comparable to, or even higher than, that observed for the other aptamers. Variant S1 performed best overall, showing 38% expression without doxycycline and a subsequent reduction to 1.5%, which corresponds to a 25-fold dynamic range. Because yeast roadblock regulation is strongly influenced by aptamer structural stability, this switching suggests substantial structural differences between ligand-bound and unbound states.

In human cells, we used G12 to control alternative splicing^24^. We decided to engineer a splice-regulating riboswitch, based on its reliable regulatory performance in human cells across target genes and cell types^8^. The 3’ splice site was incorporated into P1 of G12 in such a way that its recognition was only allowed in absence of the ligand, which lead to inclusion of a synthetic exon with multiple premature stop codons that prevented translation (Fig. 4c). Upon doxycycline binding, conformational changes stabilised P1 and sequestered the 3’ splice site, promoting exon exclusion. Although the riboswitch context was originally optimised for the tetracycline aptamer, the doxycycline aptamer showed outstanding regulatory performance without any further adaptations. In comparison to the tetracycline aptamer, G12 responded at lower concentrations than the tetracycline aptamer resulting in an overall higher dynamic range (Fig. 4c, d). Assessment of the effects of this riboswitch on single cell level by cytometry revealed a distinct effect on reporter gene expression and clear separation of cell populations depending on addition of doxycycline (Figure S13, and Table S8). The high ligand specificity of the riboswitch G12 was also proven in vivo by no response to five derivatives of doxycycline (Figure S14).

### Single-molecule experiments identify a P1-independent intermediate for doxycycline binding

**Fig. 5:**
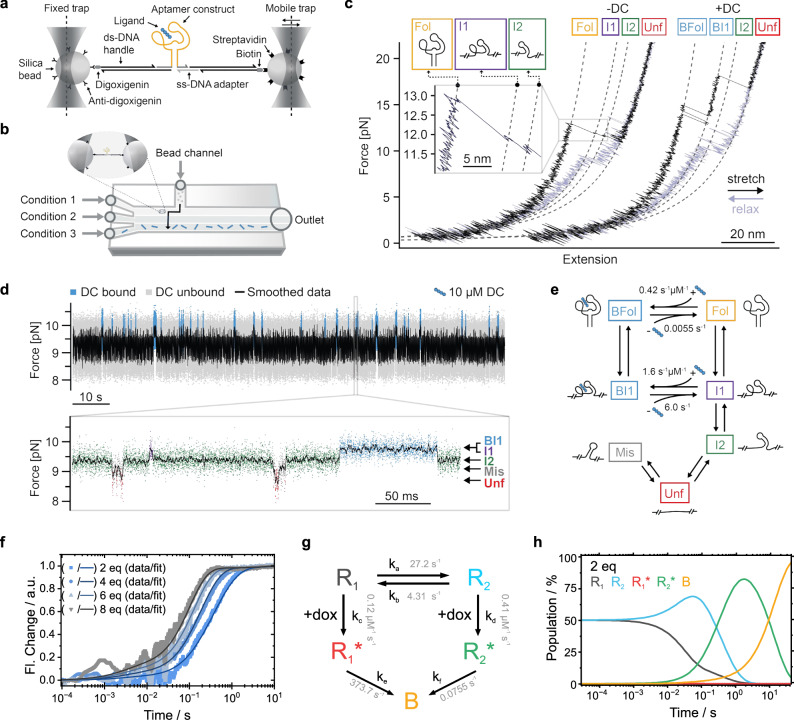
Investigation of structural and binding dynamics of G12. **a** Schematic of the optical tweezer setup with the aptamer immobilised by hybridisation to DNA handles between two silica beads. **b** Schematic of the microfluidic setup enabling single-molecule measurements with varying buffer and ligand concentrations. **c** Representative force-extension curves of the short P1 stem variant of G12 with and without doxycycline. Note that the extension axis does not show absolute values, as the traces have been offset for side-by-side comparison. The identified intermediates are modelled via WLC fits (see Methods) and corresponding structural models are indicated above the curves. **d** Top, representative passive mode trace of the G12 variant without a P1 stem. Bottom, zoomed-in section with Hidden Markov Modelling (see Material and Methods) revealing 5 states: bound intermediate 1 (blue), intermediate 1 (purple), intermediate 2 (green), misfolded (grey) and unfolded (red). **e** Folding and binding network of G12 and doxycycline as determined by SMFS. **f**) Experimental data from fluorescence stopped flow experiments and the fitted curves from the best fitting model describing the binding dynamics. The time observing fluorescence is plotted on the *x*-axis with the observed change in fluorescence in comparison to the starting intensity plotted on the *y*-axis. Experimentally measured data at different aptamer-to-doxycycline ratios are shown as data points, with the model fit shown as a coloured line. **g** Schematic representing the different conformations required to explain the observed binding dynamics. The aptamer has two unbound conformations (R_1_, R_2_), two intermediate bound conformations (R_1_*, R_2_*) and one fully bound conformation (B). The unbound conformations are in equilibrium with a preference to fold into R_2_. Association rates are indicated at their respective steps. **h** Modelling of the populations of the different conformations over time and binding to doxycycline, best explaining the observed binding behaviour. The graphs were calculated for 2 equivalents of aptamer to doxycycline and were started with a 1:1 ratio between the unbound conformations R_1_ and R_2_.

Given the high regulatory performance of G12, we next investigated the structural determinants of its properties using SMFS. By immobilising the aptamer via DNA linkers between two optically trapped beads (Fig. 5a)^61^ and transferring the molecule between microfluidic channels under different conditions (Fig. 5b)^62^, we could apply controlled pulling forces and observe folding, unfolding, and ligand binding in real time.

We first probed a G12 variant with a 5-bp P1 stem (short stem). In force-ramp experiments (Fig. 5c), the aptamer unfolded in a major peak at ~13 pN, followed by two short-lived on-pathway intermediates, denoted I1 and I2 (representative traces in Figure S15a). Addition of doxycycline markedly stabilised I1, which led to unfolding at forces higher than the main peak, while the main peak’s unfolding force remained unchanged (Fig. 5c and Figure S15a, S15b). This indicates that I1 alone is competent to bind doxycycline. Repeated transitions between the folded state (BFol) and the ligand-bound I1 (BI1) suggest that doxycycline not only stabilised I1 but may also increase the refolding rate toward BFol. Upon relaxation, the aptamer refolds, albeit with pronounced hysteresis (grey traces in Fig. 5c). Contour-length fits (dashed lines; Table S9) allowed assignment of coarse structural models: I1 corresponds to a conformation with P2 and P3 folded and P1 fully unfolded, whereas in I2 only the P3 stem remains structured.

To assess the role of P1 length, we examined constructs with an extended P1 (long stem, 12 bp) and one lacking P1 entirely (no stem). As expected, the long-stem construct exhibited a larger contour-length gain and a slightly higher unfolding force (Figure S15a). Notably, P1 unfolding forces remained independent of doxycycline, and the extent of I1 stabilisation was identical to that observed for the short-stem construct. Interestingly, the no-stem construct also showed doxycycline-dependent stabilisation of I1 comparable to the other two constructs, confirming that I1 alone is capable of binding doxycycline (Figure S15a, S15c).

For reference, we performed identical measurements on the tetracycline aptamer. Unlike G12, this aptamer unfolded in a single cooperative peak under both ligand-free and ligand-bound conditions, with tetracycline increasing the P1 unfolding force (Figure S15a, S15b). This behaviour reflects the essential role of P1 in tetracycline binding. In contrast, G12 can already bind its ligand in an intermediate conformation (I1) that lacks P1, revealing a distinct binding mechanism.

To monitor doxycycline binding in real time and extract kinetic rates, we performed passive-mode measurements, applying a constant average force to a single molecule in the presence of doxycycline. Using the no-stem construct enabled continuous observation of a single molecule for hundreds of seconds, capturing near-equilibrium transitions between multiple folding intermediates (Fig. 5d). Using a Hidden Markov Model (HMM) analysis^63^, we resolved a network of five states: unfolded (Unf), misfolded (Mis), I2, I1, and ligand-bound I1 (BI1). Based on contour-length changes (Table S9) and state connectivity, a clear folding and binding model emerges (Fig. 5e): starting from Unf, G12 can either misfold into Mis or fold the P3 stem (I2). From I2, the aptamer transitions to I1, which involves P2 folding and formation of the tertiary contacts required for doxycycline binding. BI1 has the same contour length as I1, supporting that the necessary structure for binding is already present in I1. As expected, BI1 lifetimes are substantially longer than I1, reflecting stabilisation by doxycycline.

Kinetic analysis over a range of force biases (Figure S16) demonstrated that binding occurred exclusively to I1, with on-rates precisely tracking the I1 population. This supports a conformational capture mechanism and further corroborates that the tertiary contacts required for recognition are pre-formed in I1. The deduced off-rate for BI1 was 6.0 s⁻¹, corresponding to an affinity of 3.8 µM for the no-stem construct, considerably weaker than affinities measured for full-length G12 via isothermal titration calorimetry and fluorescence titration spectroscopy (Figs. 3b, c). To explore this discrepancy, we assessed the role of the P1 stem on binding kinetics using the short-stem construct. Equilibrium measurements at constant force were not feasible, as the short-stem construct predominantly occupies BFol at relevant forces. Instead, we implemented a jump protocol, shuttling the molecule between channels with and without doxycycline to determine binding and unbinding rates at near-zero force (Figure S17). These experiments revealed that P1 strongly reduces the off-rate (0.0055 s⁻¹) while only modestly affecting the on-rate (0.42 s⁻¹ µM⁻¹) (Figure S17, and Table S10), yielding an affinity of 13.1 nM that closely matched bulk measurements. We therefore conclude that P1 is not required for doxycycline recognition, as the I1 intermediate, which lacks P1, is already binding-competent. Instead, P1 acts as a kinetic gatekeeper (Table S11), strongly slowing ligand dissociation, stabilising the bound state, and ensuring the high binding affinity observed in bulk measurements.

### Two-step binding of doxycycline can be achieved by two distinct pathways

To gain further insights into the aptamer’s binding dynamics, stopped-flow spectroscopy was performed utilising the intrinsic fluorescence changes of doxycycline caused by binding to the aptamer. Four mixing experiments with increasing ligand concentrations (2, 4, 6 & 8 equivalents) and G12 were conducted (Fig. 5f). The fluorescence intensity increased during the ligand binding and the binding event accelerated from 3 s to 1 s with higher equivalents of ligand. The binding mechanism of doxycycline to G12 was analysed with a kinetic modelling (KM) approach. Starting with a simple one-step binding process, the model was progressively refined by incorporating additional conformational states, intermediate steps and reverse reactions (Table S12). The comprehensive kinetic modelling analysis is described in detail in the Supplementary Information. All tested models were compared by means of the goodness of fit as well as the number of parameters used. The best model revealed two initial conformations of ligand-free aptamer in equilibrium (Fig. 5g, and Table S13). Both starting conformations (R_1_ and R_2_) bind doxycycline via a two-step binding mechanism, involving an initial ligand-aptamer association followed by a ligand-independent conformational rearrangement. The population plot based on this model indicates that binding primarily occurred in one of the two starting conformations (R_2_) and its intermediate (R_2_*). At the beginning of the measuring window the R_1_/R_2_ equilibrium rapidly shifted towards R_2_. Some of the doxycycline was also bound by R1 to form the intermediate R1*. However, its population remained negligible due to its fast rearrangement rate (k_e_), which is significantly faster than its formation rate (k_c_).

These results suggest two aptamer conformations in equilibrium, while one (R_2_) facilitates faster ligand binding. However, in the second binding step, its intermediate R2* requires more extensive rearrangement towards the final bound conformation (B). In contrast, the conformation of R_1_ slows down initial interaction with the ligand, while its intermediate R_1_* allows for rapid conformational rearrangement.

### Magnesium ions are required for binding doxycycline but not for structure formation

Finally, we investigated whether doxycycline binding to G12 depends on magnesium ion stabilisation of the tertiary structure, as it has been shown for the tetracycline-binding aptamer^64,65^. To this end, we performed single-molecule force-ramp measurements on the short-stem construct in the presence of doxycycline at varying Mg^2+^ concentrations (Figure S18). In the absence of both Mg^2+^ and doxycycline, the folding pathway of G12 was essentially identical to that observed in the presence of Mg^2+^ (Fig. 5c left). The slightly lower overall unfolding forces are likely due to the reduced salinity without Mg^2+^. Consistently, melting curve analysis showed that G12 folding does not depend on Mg^2+^ (Figure S19a, b, and Table S14).

In force-jump experiments at 1 µM doxycycline, the binding probability increased from ~30% in 50 µM Mg^2+^ to ~100% in 5 mM Mg^2+^ (Figure S18b, c), indicating that magnesium ion availability limits doxycycline binding at micromolar concentrations. Note that the events where doxycycline did not bind at 5 mM Mg^2+^ can be entirely explained by insufficient equilibration times at low force. From the ~30% binding probability at 50 µM Mg^2+^, we estimate a K_D_ of ~120 µM of Mg^2+^. To further quantify the magnesium ion dependence of binding, we performed FTS at varying Mg^2+^ concentrations (Figure S11g, h). In the absence of Mg^2+^, no fluorescence was detected, indicating a lack of doxycycline binding to G12, whereas fluorescence increased progressively with magnesium ion concentration. With detectable binding emerging at >40 µM Mg^2+^, the results confirm a dependency on magnesium ions for binding doxycycline with a K_D_ of 210 µM. Literature values for Mg^2+^ binding to tetracyclines are in the hundreds of micromolar range^66^, consistent with the interpretation that Mg^2+^ binding to doxycycline is the limiting step, while no additional magnesium ion-mediated structural stabilisation of G12 is required for ligand binding.

## Discussion

Engineering of synthetic riboswitches greatly depends on the availability of well-characterised, regulatory aptamers. We discovered a regulatory RNA aptamer responding to doxycycline with high regulatory response in yeast and human cells. Motivated by the question of whether and how regulatory aptamers for the engineering of synthetic riboswitches can be efficiently identified, we ran a conventional, column-based SELEX and a Capture-SELEX in parallel. Our objective was to test the hypothesis that RNA Capture-SELEX is a more efficient method for enriching regulatory aptamers. The direct comparison provided the proof for the enhanced efficiency of RNA Capture-SELEX, as previously suggested^36^. Bioinformatical analyses of the two selections confirmed faster and more effective enrichment of regulatory aptamers by Capture-SELEX. However, the best candidate, aptamer G12, was identified from column-SELEX, against all statistical probability. The inability to hybridise to the capture-oligonucleotide prevented selection with Capture-SELEX, despite the higher efficiency of this method to enrich sequences with regulatory properties. These results should be understood in the context with the limitation of only two in vitro selections being compared. Yet, the stark difference in enrichment of regulatory aptamers by RNA Capture-SELEX makes it the preferable method for the selection of regulatory aptamers.

Although RNA motifs that bind doxycycline have been reported previously^67,68^, the aptamer G12 is the only suitable for riboswitch engineering. Its sequence and structure show no similarity to binding motifs described earlier. While all doxycycline-binding RNA sequences exhibit affinities in the low nanomolar range, none of the previously described motifs has been shown to elicit regulatory response in vivo. This is even more astonishing as one of them has been selected as part of an aptazyme with self-cleavage activity in vitro^67^. In contrast, G12 was readily applied for gene regulation in both yeast and human cells, highlighting its versatility and robust regulatory performance across species. Its potential was highlighted by the comparison with other regulatory aptamer domains. While the aptamers binding tetracycline, neomycin, and tobramycin possess robust regulatory properties in yeast, G12 showed improved dynamic range and expression levels. The use of the roadblock regulation has been shown to be difficult in human cells, possibly due to the strong helicase activity of the ribosome^69,70^. Therefore, we decided to utilise the regulatory potential of the doxycycline aptamer for splice regulation. G12 was applied to an existing riboswitch design, without further need to adapt the context. As a reliable synthetic tool, it can now be deployed for control of gene expression, including biomedical studies, similar to the investigation of the role of CD20 in resting B lymphocytes^71^. Its portability, compact size, robust function and good pharmacokinetic properties of the ligand allow its broad applicability, while its distinct ligand specificity enables orthogonal gene regulation.

Understanding the regulatory potential of G12 required its thorough characterisation and provides the basis for sophisticated riboswitch engineering. Detailed mutational studies and chemical probing revealed a T-shape structure of G12 and implied complex tertiary interactions of the loops that are involved in interaction with doxycycline. Analysis of the binding properties of the aptamer revealed that G12 binds doxycycline in a two-pathway mechanism in two steps and with an affinity in the low nanomolar range, while strictly discriminating against close derivatives of doxycycline. The two-step binding mechanism was already reported for other regulatory aptamers^40–42^, the binding dynamics follow two pathways. This specific binding kinetic is caused by two unbound conformations in equilibrium (R_1_ and R_2_) that are both binding-competent. The association constants of individual binding steps calculated from stopped flow experiments are supported by experimental data from single-molecule force spectroscopy experiments. Although these methods probe different aspects of the binding process, their results are highly complementary, and key metrics such as the doxycycline binding rate constants and the K_D_ are highly consistent across methods. The aptamer’s high affinity may be explained by its unusually low off-rate. Thus, G12 aligns with observations from other regulatory aptamers (tetracycline, neomycin and ciprofloxacin), for which low off-rates have been proposed as a key factor underlying regulatory performance^36,41,42^. Interestingly, the role of magnesium ions differ between them. The tetracycline aptamer requires magnesium ions for correct folding and ligand binding^64^, whereas the neomycin aptamer is completely independent^72^. Here, we observed an intermediate I1 for the doxycycline aptamer even in the absence of magnesium ions, i.e., the aptamer does not require magnesium ions for correct folding, yet depends on it for doxycycline binding, as supported by SMFS.

Although a direct structural assignment of the proposed states from the stopped-flow spectroscopy is not possible, it is intriguing that our SMFS experiments likewise find two binding-competent states of the aptamer. These binding-competent conformations represent the fully folded aptamer (Fol) and a partly folded conformation (I1). Binding of doxycycline leads to a pronounced stabilisation of this intermediate conformation (BI1) within the aptamer, which is independent from the P1 stem. Observations of the folding kinetics revealed a distinct order of structures forming (I2- > I1->Fol), where after initial folding of the P3 stem (I2), I1 emerges in a single cooperative step with no separation between secondary-structure formation and the establishment of tertiary contacts between loops L2 and L3. This behaviour contrasts sharply with several natural aptamers, including the *pbuE* adenine,^47^*add* adenine^50^, and *xpt-pbuX* guanine riboswitches^73^, in which P2 and P3 fold independently and tertiary interactions form only in a subsequent step. The full folding cooperation of secondary and tertiary structure in the I2- > I1 transition likely reflects the short P2 stem, which cannot stably form in isolation but only in the context of additional loop interactions. In many aptamers, including the tetracycline aptamer and other previously tested aptamer domains, ligand binding involves a stabilisation of the whole aptamer, including the P1 stem^50,73^. Such concerted structural interaction is central to their regulatory function. This principle applies broadly to both synthetic and natural aptamers^74,75^. In striking contrast, our results reveal a distinct mechanism for G12 that relies on I1 as the key ligand-binding structure, while P1 plays only a secondary role, namely in modulating kinetic stability. This mechanism distinguishes G12 from classical riboswitches and illustrates a modular architecture in which the core (lacking P1) determines specificity, while peripheral stems exclusively modulate kinetic stability^50,73^. This mechanism has direct implications for gene regulation applications, e.g. in yeast. Here, efficient regulation via a roadblock mechanism requires a balance between low structural stability in the free state, which permits translation in the absence of ligand, and high stability in the bound state to prevent leaky expression^76^. G12 meets these requirements with its tolerance to P1 shortening and a mechanically weak tertiary structure in the free state, while transitioning into an overall strong structure upon binding to doxycycline.

In sum, we provide evidence that RNA Capture-SELEX selects regulatory aptamers efficiently. We offer mechanistic insights into different selection processes that led to the identification of G12. Together, these findings facilitate the selection of regulatory aptamers that are crucial for riboswitch engineering. Furthermore, we identify G12 as the doxycycline-binding RNA aptamer with demonstrated regulatory activity in vivo. Owing to its remarkable regulatory potential and the widespread availability of its ligand, G12 represents a prime candidate for engineering synthetic riboswitches in eukaryotes. Its binding kinetics and structural dynamics upon ligand binding support the two-step binding mechanism, low off-rates and structural stabilisation that are key features of regulatory aptamers. However, with its two alternative binding pathways and a distinct stabilisation of an intermediate conformation, G12 exhibits previously unreported behaviour. These insights deepen our current knowledge of regulatory aptamers and riboswitch function and highlight the necessity for selection and comprehensive characterisation of regulatory aptamers. Overall, the discovery of G12 advances our understanding of the requirements for RNA-mediated regulation and provides a versatile doxycycline-responsive regulator with broad potential across synthetic biology and biomedicine.

## Methods

### Materials

Chemicals were purchased from Carl Roth (Karlsruhe, Germany) and used without any further purification unless otherwise stated. Molecular biology enzymes were purchased from New England Biolabs (Ipswich, USA). All Oligonucleotides (Table S15) were synthesised either by Sigma-Aldrich (St. Louis, USA) or Microsynth (Balgach, Switzerland). Single RNA and DNA nucleotides were purchased from Sigma-Aldrich (St. Louis, USA). ^32^P-*α*-ATP was purchased from Hartmann Analytic (Braunschweig, Germany). Epoxy-activated Sepharose 6B was purchased from Cytiva (Marlborough, USA). Poly-Prep Chromatography columns were purchased from BIO-RAD (Hercules, USA). T7 RNA polymerase and Taq DNA polymerase were prepared in-house. All cell lines were obtained from the DMSZ (German Collection of Microorganisms and Cell Cultures).

### Pool preparation

DNA template for the Capture-SELEX pool was assembled by PCR with 2 µM of the primers Pool_fw (5’- CCA AGT AAT ACG ACT CAC TAT AGG GCA ACT CCA AGC TAG ATC TAC CGG T-3’) and Pool_rev (5’-AGT GAA AAG TTC TTC TCC TTT GCT AGC CAT TTT-3’) and 70 nM of the template Pool_oligo_rev (5’-AGT GAA AAG TTC TTC TCC TTT GCT AGC CAT TTT NNN NNN NNN NTA GAA GCC AGT AGN NNN NNN NNN NNN NNN NNN NNN NNN NNN NNN NNN NNN NNN ACC GGT AGA TCT AGC TTG GAG TTG CCC-3’, Table S15) in 20 mM TRIS pH 8.8, 10 mM (NH_4_)_2_SO_4_, 10 mM KCl, 2 mM MgSO_4_ and 0.1 % (v/v) Triton X-100 using Taq polymerase (prepared in-house). The PCR amplification was carried out with an initial denaturation of 2 min at 96 °C, followed by 5 cycles of 30 sec at 96 °C, 30 sec at 57 °C and 30 sec at 72 °C with a final 3 min at 72 °C. A T7 promoter upstream of the pool was used for in vitro transcription by T7 RNA polymerase. The RNA pool used for both selections was transcribed in 200 mM TRIS pH 8.0, 20 mM Mg(OAc)_2_, 20 mM DTT, 4 mM ATP/UTP/GTP/CTP, 2 mM spermidine. Transcription products were ethanol-precipitated, gel-purified and stored for later use.

### In vitro selection (SELEX)

The in vitro selection was performed in accordance with the MAPS guidelines (minimum aptamer publication standards)^77^.

For the first round of selection, non-radioactive labelled molecules of prepared RNA were used. Afterwards, RNA pools were transcribed from round 2 on in 100 µL with the addition of 33 nM ^32^P-*α*-ATP in 200 mM TRIS pH 8.0, 20 mM Mg(OAc)_2_, 20 mM DTT, 4 mM ATP/UTP/GTP/CTP, 2 mM spermidine and 15% DMSO using T7 RNA polymerase (prepared in-house), resulting in ^32^P body-labelling of the transcribed RNA. Transcribed RNA was precipitated using ethanol and NH_4_OAc and resolved in ddH_2_O. Radioactivity was measured using a liquid scintillation analyser (TriCard 2800 TR, PerkinElmer, Waltham, USA) to determine RNA concentration.

For column-based SELEX, epoxy-activated Sepharose 6B (Cytiva, Marlborough, USA) was used. Doxycycline was coupled according to the manufacturer’s instructions at pH 12.0, utilising the hydroxy groups for immobilisation. Following the same steps but leaving out doxycycline, uncoupled column material was prepared for pre-selection. The first round of selection was performed with a non-radioactive-labelled RNA pool of 1.2×1016 molecules. Every round the RNA pool first underwent pre-selection by allowing the RNA pool in 500 µL 1x Capture-SELEX buffer (CSB, 40 mM HEPES pH 7.5, 250 mM KCl, 20 mM NaCl, 5 mM MgCl_2_, 0,01 Tween-20) to flow through the pre-selection column of 0.5 mL Sepharose 6B without an immobilised target, which removed any sequences binding to the column material. The pre-selection column was then washed with 1 mL 1x CSB and the resulting 1.5 mL pre-selected RNA in 1x CSB was collected and allowed to flow through 1.5 mL of doxycycline-coupled Sepharose 6B. Next, the column was washed either 12x or 18x with 1.5 mL 1x CSB (detailed selection parameters see Supplementary Table S1). In rounds with a counter-selection step, 4.5 mL of 500 µM kanamycin A in 1x CSB were washed through the column after 12 washing steps and followed by 6 further washing steps. Counter-selection was performed for three consecutive rounds to increase specificity^78^. RNA bound to the column was eluted by allowing 4.5 mL 500 µM doxycycline in 1x CSB to flow through the column. The eluted RNA was recovered through ethanol precipitation and resuspended in 50 µL MQ H_2_O. The RNA was then reverse-transcribed using SuperScript II reverse transcriptase (Thermo Fisher Scientific, Waltham, USA) and amplified using Taq DNA polymerase with 1x ThermoPol buffer, 0.2x SSII first strand buffer, 2 mM DTT, 1.5 mM MgCl_2_, 0.3 mM dATP/dTTP/dGTP/dCTP and 3 µM Pool_fw and Pool_rev. The cDNA pool was then stored and used for transcription of RNA for the next round of selection.

Capture-SELEX was started by mixing 6 ×1012 molecules of the prepared RNA pool with 2 nmol of capture-oligonucleotide in 200 µL of 1x CSB. The mixture was then heated to 65 °C for 5 min and cooled down to 21 °C for 30 min using a thermoblock. The resulting RNA-capture-oligonucleotide complex was mixed with 1 mL paramagnetic Dynabeads M-270 (Thermo Fisher Scientific) at room temperature with gentle agitation for 1 h. The beads washed in advance three times with 500 µL bind & wash buffer (BW, 5 mM TRIS, 0.5 mM EDTA, 1 M NaCl, 0.01% Tween-20) and then finally equilibrated in 300 µL 1x CSB. After the immobilisation of the RNA pool, the beads were collected with a magnetic rack and the supernatant was discarded. The beads were washed three times with 500 µL 1x CSB and incubation under mild agitation. Afterwards, the immobilised RNAs were specifically eluted by incubation at room temperature with 100 µM doxycycline in CSB for 5 min. The eluted RNA in the supernatant was recovered, ethanol precipitated in the presence of 0.3 M NaOAc (pH 6.5), air-dried, dissolved in 50 µL MQ-H_2_O and reverse transcribed using Superscript II reverse transcriptase (Thermo Fisher Scientific) and Taq DNA polymerase (homemade), as described above. The PCR products were analysed using agarose gel electrophoresis and 10 µL were used as template for in vitro transcription for the subsequent selection round.

### In vivo screening in *S. cerevisiae*

In vivo screening was performed in *S. cerevisiae* RS453*α* (MAT*α ade*2-1 *trp*1-1 *can*1-100 *leu*2-3 *his*3-1 *ura*3-52, received from group of Norbert Sauer^79^). RT-PCR pools from SELEX were cloned into the pCBB06 backbone via homologous recombination^53^. The in vivo screening system utilised a plasmid containing a GFP gene as readout for regulatory activity, containing the aptamer candidate in its 5’UTR, and mCherry as a constitutively expressed control. The backbone pCBB06 was digested with NheI-HF and AgeI-HF and complementary overhangs were added to the sequences obtained from the SELEX pools via PCR using the primers Yeast-HR_fw (5’- CTC GTC ATT GTT CTC GTT CCC TTT CTT CCT TGT TTC TTT TTC TGC ACA ATA TTT CAA GCT ATA CCA AGC ATA CAA TCA ACT CCA AGC TAG ATC TAC CGG T-3’) and Yeast-HR_rev (5’- CAC CCT CTC CAC TGA CAG AAA ATT TGT GCC CAT TAA CAT CAC CAT CTA ATT CAA CAA GAA TTG GGA CAA CTC CAG TGA AAA GTT CTT CTC CTT TGC TAG C-3’). Both fragments were then transformed into yeast by electroporation using a MicroPulser Electroporator (Bio-Rad, Hercules, USA) and assembled in the cells via homologous recombination. The transformed cells were first cultured in 250 mL SCD-Ura medium (0.2% YNB w/o AA (Difco), 0.55% ammonium sulphate, 2% glucose, 12 µg/mL adenine (Sigma-Aldrich), 1x MEM amino acids (Sigma-Aldrich) in a baffled flask for 48 h at 30 °C and 125 rpm shaking. 25 mL of this culture were transferred to fresh 225 mL SCD-Ura medium and cultured for another 24 h at 30 °C and 125 rpm shaking to reduce the risk of multiple plasmids in a single cell. The cells were sorted using fluorescence-activated cell sorting (Beckman Coulter, Brea, USA) for viability, correct mCherry expression and GFP expression. Selected cells were transferred to SCD-Ura plates (SCD-Ura medium with 2% agarose) and cultured. Single clones from these plates were used to inoculate 200 µL per well in 96-deep-well round bottom plates (Thermo Fisher Scientific) and incubated for 24 h at 30 °C and 1200 rpm shaking. 20 µL of this culture each were used to inoculate 180 µL of fresh SCD-Ura in one plate without and one with 100 µM doxycycline and incubated for another 24 h. 20 µL of the culture were transferred to 180 µL PBS for GFP and mCherry fluorescence measurements using a CytoFLEX S (Beckman Coulter, Brea, USA). About 20 000 cells were measured per sample, the GFP fluorescence median value was taken and normalised to the mCherry fluorescence^53^.

### Mutational studies

For mutational studies in yeast, mutants of G12 were ordered as DNA oligonucleotides (Sigma-Aldrich, Supplementary Table S15) and hybridised by heating to 95 °C and slowly cooling down. pCBB06 was digested with NheI and AgeI and the hybridised oligonucleotides were ligated into the backbone using T4 DNA ligase. The resulting plasmids were transformed into *S. cerevisiae* RS453*α* via electroporation using a MicroPulser Electroporator (Bio-Rad, Hercules, USA). The cells were spread on SCD-Ura plates and cultured for 48 h at 30 °C. Single clones were picked to inoculate individual wells of a 96-deep-well plate containing 200 µL of SCD-Ura and cultured for 48 h at 30 °C and 1200 rpm. From the pre-culture, 20 µL were used to inoculate one well each on one plate containing 180 µL SCD-Ura and on one plate containing 180 µL SCD-Ura with 100 µM doxycycline and cultured for 24 h at 30 °C and 1200 rpm. For fluorescence measurements, 20 µL from each well were transferred to 180 µL PBS in a 96-well plate and measured on a CytoFLEX S (Beckman Coulter, Brea, USA) as previously reported^53^. Measurements of individual sequences were always performed twice in biological triplicates.

For randomised mutation studies of the variant M10, an oligonucleotide with a mutation rate of 4.5% per nucleotide (e.g. a position originally encoding an “A” would be synthesised as 95,5% A, 1,5% T, 1,5% G, 1,5% C) from G7 to C51 (Supplementary Table S11) was ordered from Microsynth and assembled by PCR using Q5 DNA polymerase with the primers Yeast_HR_fw and Yeast-HR_rev (Sigma-Aldrich) adding overhangs for homologous recombination into pCBB06. 1 µg of NheI and AgeI digested pCBB06 with 3-times molar excess of insert was transformed by electroporation into *S. cerevisiae* RS453*α* using a MicroPulser Electroporator. The transformed cells were recovered in 8 mL Sorbitol-YEPD (1% yeast extract, 2% peptone, 4% glucose, 0.5 M sorbitol) and incubated 1 h at 30 °C shaking at 130 rpm. The pre-cultured cells were transferred to 100 mL SCD-Ura and incubated in a baffled flask for 48 h at 30 °C and shaking at 125 rpm. 20 mL of the culture were transferred to fresh 80 mL SCD-Ura medium and cultured for further 24 h. For FACS, the original construct M10 was first measured using a CytoFLEX SRT (Beckman Coulter, Brea, USA) in the absence of doxycycline to set a gate covering the measured events 33% below and 33% above the medium GFP intensity. Afterwards, the cells of the partly mutated pool were measured until 100 000 cells were sorted showing GFP expression in the set reference gate. The selected cells were transferred to 100 mL SCD-Ura and regrown for 48 h at 30 °C and 125 rpm. 1 mL was transferred to 50 mL SCD-Ura containing 100 µM doxycycline and incubated for 24 h at 30 °C and 125 rpm shaking. For the second sorting step, the original construct M10 was again measured first in the presence of 100 µM doxycycline. Three gates were set: One gate was the gate used for the initial sorting, defined as non-regulatory since no change in expression would have occurred in these mutants. A second gate covering all events 33% below and 33% above the GFP expression of M10 with 100 µM doxycycline, defined as functional as these mutants would have shown similar expression levels to M10. A third gate was set that covered all events showing GFP expression lower than the second gate, defined as improved function since these mutants would show even stronger reduction in expression compared to M10. The sorted cells from the third gate were plated on SCD-Ura plates and used for flow cytometry to confirm their switching (as described for the in vivo screening). The cells from the first two gates were re-cultured, the plasmids isolated, and the area of the aptamer PCR was amplified adding Illumina adaptors in preparation for NGS.

### Next-generation sequencing

For next-generation sequencing, Illumina adaptor sequences were added to the SELEX pool via PCR using a Q5 DNA polymerase and primers annealing in the constant regions of the pool (Figure S1a). For each round, a specific forward primer (NGS-index) was used containing an individual 6-nucleotide barcode to allow multiplexing of samples in combination with the Pool_rev primer (5’- AGT GAA AAG TTC TTC TCC TTT GCT AGC CAT TTT-3’, Table S15). The samples were sequenced by GENEWIZ (Leipzig, Germany) using an Illumina NovaSeq X sequencer (Illumina, San Diego, USA) using the P5 and P7 adaptor sequences. A Python-based pipeline was developed for the extraction, mutation analysis, and visualisation of aptamer sequences from high-throughput sequencing data. This pipeline first extracted aptamer regions from both forward and reverse raw sequences by identifying specific primer markers and performing reverse complementation where necessary. Extracted aptamers were then subjected to global pairwise alignment against a reference aptamer sequence, followed by quality control based on length and alignment similarity. Sequences below 10 reads were filtered out.

For analysis of the mutational studies, the pipeline analysed mutations by comparing aligned aptamer sequences to the reference, quantifying base substitutions and indels. Finally, it calculated and visualised mutation ratios between two samples (e.g., maintain *vs*. loss functions) using Log2 fold change of frequencies, generating comprehensive plots with Matplotlib that include total mutation ratios, detailed base-specific mutation ratios with significant sites highlighted, and read coverage. All mutation counts and ratios were also exported to a CSV file for further analysis.

### Identification and analysis of motifs

To identify sequence motifs enriched during the two SELEX strategies, we performed de novo motif discovery using the MEME algorithm (MEME Suite v5.5.8)^54^. FASTA files containing the unique sequences identified through NGS analysis were generated for each selection round and SELEX strategy. These files served as input for MEME. Motif discovery was configured to detect up to 10 enriched motifs per selection round, with motif lengths ranging from 6 to 12 nucleotides and a runtime limit of 14400 sec. MEME output files from individual selection rounds were consolidated into a single file per SELEX strategy. Motifs were filtered to retain only those with statistically significant E-values (<0.05). In cases where the same motif appeared in multiple rounds, only its first occurrence was retained to prevent redundancy and bias in downstream analyses. To map motifs back to the sequences, we employed the FIMO algorithm (default p-value threshold: 1e-4)^55^. Recognising that motifs may evolve over successive selection rounds, we aligned all identified motifs using Clustal Omega and clustered them based on phylogenetic similarity. Pairwise distances were calculated using the Damerau-Levenshtein distance (via the stringdist package v0.9.15)^80^, and hierarchical clustering was performed using complete linkage (hclust function base R v4.5.1)^81^. Clades were defined using cutree, with a maximum pairwise distance threshold of 0.4.

This clustering approach enabled the identification of distinct motif groups, which were visualised as clades across selection rounds using the ggplot2 package (ggplot2 v4.0.0)^82^ in stacked bar plots. Additionally, the temporal dynamics of each motif were illustrated using a combined dendrogram and bubble plot, highlighting motif abundance across the SELEX rounds and phylogenetic similarity.

### Preparation of RNA aptamers

Aptamers were transcribed from hybridised oligonucleotides using the respective aptamer sequence (G12_IVT, G12_M3_IVT, G12_S1_IVT, C01_IVT, Table S15) and a complementary oligonucleotide for the T7 promoter (T7_fw, 5’-CGA ATT CCA AGT AAT ACG ACT CAC TAT AGG-3’, Table S15). Run-off transcription was done in 200 mM TRIS pH 8.0, 20 mM Mg(OAc)_2_, 20 mM DTT, 4 mM ATP /UTP/GTP/CTP, 2 mM spermidine using T7 RNA polymerase (prepared in-house) and incubating for 6 h at 37 °C. Mg-pyrophosphate was dissolved in the solution by dropwise adding 0.5 M EDTA pH 8.0, the reaction products were ethanol-precipitated and separated on a 10% denaturing polyacrylamide gel. The aptamer RNA was detected by ultraviolet shadowing and the respective band cut from the gel, crushed and left over night in 300 mM NaOAc solution. The supernatant was ethanol-precipitated, the RNA pellet resuspended in MQ H_2_O, the concentration measured using a NanoPhotometer N60 (Implen, Munich, Germany) and frozen for later use.

### Isothermal titration calorimetry

Gel-purified aptamers and a 100 µM ligand solution were prepared in CSB. ITC measurements were performed using a MicroCal PEAQ-ITC (Malvern Instruments, Malvern, United Kingdom), containing the RNA in the simple cell (200 µL) and the ligand in the syringe (40 µL). After thermal equilibration to 25 °C and an initial delay of 150 s, one initial injection of 0.4 µL was carried out. Serial injections of 2 µL were performed in an interval of 120 s at a stirring speed of 750 rpm. The thermal differences occurring between the sample and reference cell were integrated and plotted against the molar ratio of RNA and ligand. The dissociation constant (K_D_) from each experiment was calculated using a curve fit model provided by the MicroCal PEAQ-ITC Analysis Software (v1.1.0.1262). Each measurement was performed at least twice, and final K_D_ values were calculated from the average of the individual experiments.

### Fluorescence titration spectroscopy

Binding-induced fluorescence of doxycycline was measured using a Fluorolog FL3-22 fluorometer (Horiba, Kyoto, Japan)^37^. Measurements were performed in 2 mL FTS buffer (20 mM KPO_4_ pH 7.5, 100 mM NaCl 10 mM MgCl_2_) with 0.5 nM doxycycline, 1 nM tetracycline, 5 nM 7-chloro-tetracycline, 20 nM anhydrotetracycline, 20 nM minocycline or 5 nM sancycline and varying concentrations of aptamer. The excitation wavelength was set to 470 nm and the fluorescence spectra acquired from 450 to 600 nm in 1 nm increments with an integration time of 0.1 s and slits set to 4 nm. Measurements were taken from buffer only, doxycycline in buffer and increasing concentrations of aptamer. The aptamer was titrated in small volumes to not exceed a total volume of 4%. After each titration step, the solution was stirred and allowed to equilibrate for 5 min before data collection. The fluorescence peak area around 490 nm was averaged, normalised for background fluorescence and plotted against the RNA concentration. The fractional saturation (F_S_) was determined as followed:

1 $${F}_{S}=\frac{S-{S}_{\min }}{{S}_{\max }-{S}_{\min }}=\frac{\Delta S}{\Delta {S}_{\max }}$$

∆S being defined as the difference between the fluorescence signal (S) and the background signal (S_min_) at each titration step. ∆S_max_ is defined as the difference between the signal at complete saturation S_max_ and the S_min_. As the used tetracyclines act as the acceptor molecule, the degree of saturation equals F_S_. The concentration of free aptamer was calculated as:

2 $${c}_{{free}\,{aptamer}}={c}_{{total}\,{aptamer}}-({F}_{S}\times {c}_{{Tetracycline}})$$

The saturation isotherm was used to calculate the dissociation constant (K_D_):

3 $${F}_{S}=\frac{{B}_{\max }\times {c}_{{free}\,{aptamer}}}{{K}_{D}\times {c}_{{free}\,{aptamer}}}$$

The dissociation constant was calculated for each construct from two measurements as previously described^37^.

### Control of mRNA processing in human cells

Regulation of mRNA processing in human cells was tested using a dual reporter system in HeLa cells (purchased from the German Collection of Microorganisms and Cell Cultures, ACC 305), using Firefly and Renilla luciferase, and in HEK293 cells (purchased from the German Collection of Microorganisms and Cell Cultures, ACC 57), using GFP and mCherry. For luciferase measurements, a total of 1.5×10^4^ HeLa cells were seeded in each well of a 96-well plate and subsequently transfected with 22 fmol of the pWHE237 vector and 22 fmol of the pRL-SV40 vector using Lipofectamine 3000 (Thermo Fisher Scientific). The vector pWHE237 contained Firefly luciferase with the riboswitch at the 3’ end of the synthetic exon, while the vector pRL-SV40 constitutively expressed a Renilla luciferase for normalisation. The measured luminescence of the controlled Firefly luciferase was divided by the measured luminescence of the Renilla luciferase to obtain normalised luminescence values, as previously described^24^. Four hours post transfection, white DMEM with or without ligand was added and the cells were incubated at 37 °C and 5% CO_2_ for 24 h. Luciferase activity was measured with the Dual-Glo® Luciferase assay (Promega, Madison, USA). 100 µL of the Dual-Glo® reagent was added to each well and incubated for 20 min at room temperature and shaking at 450 rpm. Luminescence was measured using an Infinite M200 Pro plate reader (Tecan, Männedorf, Switzerland). Subsequently, the Dual-Glo® Stop&Glo Substrate (50X) was diluted 1:200 with Dual-Glo® Stop&Glo Buffer, and 100 µL was added to each well. Following a further 20 min of incubation at room temperature on a shaker at 300 rpm, the luminescence was measured again. Each construct was tested three times in triplicates, and the relative light units (RLU) of the firefly luciferase were normalised using the RLU of the Renilla luciferase. All samples were normalised to a positive control (pWHE237 without a synthetic exon). The dynamic range was calculated as the factor of signal increase between the expression level without ligand and the respective measurement with ligand. For single-cell fluorescence measurements, a total of 1×10^5^ HEK293 cells were seeded in each well of a 24-well plate and subsequently transfected with 200 ng of the pCMV-eGFP-G12sr vector and 200 ng of the pCMV-mCherry vector using FuGENE® HD (Promega, Madison, USA). The vector pCMV-eGFP-G12sr contained eGFP with the riboswitch at the 3’ end of the synthetic exon, while the pCMV-mCherry vector expressed mCherry constitutively for normalisation. Two h after transfection, DMEM with or without the ligand was added, and the cells were incubated for 24 h at 37 °C and 5% CO_2_. Following incubation, the cells were washed once with 1× PBS and detached using 200 µL Trypsin/EDTA solution. Subsequently, 100 µL of the cell suspension from each well was transferred into 150 µL PBS in a transparent 96-well plate and analysed using a CytoFLEX S (Beckman Coulter, Brea, USA). HEK293 cells were manually gated based on SSC-A versus FSC-A plots, followed by singlet selection using SSC-A versus SSC-H plots. A total of 10,000 events within the HEK293 gate were recorded for each sample. Mean fluorescence intensities of the FITC-A channel (GFP fluorescence) and the ECD-A channel (mCherry fluorescence) were exported from the singlet population. Each construct was analysed in two independent experiments performed in technical triplicates. The measured fluorescence of eGFP was normalised to the mCherry fluorescence.

### Cell viability assay

Cell viability was determined using the alamarBlue HS Cell Viability Reagent (Thermo Fisher Scientific). HeLa cells were seeded into transparent 96-well plate at a density of 0.5×105 cells per well. Following a 24 h incubation period, cells were treated with DMEM containing increasing concentrations (up to 500 µM) of the respective ligand (doxycycline, tetracycline, anhydrotetracycline, sancycline, minocycline, or 7-chlorotetracycline) and incubated for an additional 24 h at 37 °C and 5% CO_2_. Two h before the end of the incubation period, 10 µL of alamarBlue HS reagent were added to each well, followed by a further two h incubation. Fluorescence was subsequently measured at 560/590 nm (excitation/emission), and absorbance was measured at 570 nm using an Infinite M200 Pro microplate reader (Tecan). Absorbance at 600 nm was used as the reference wavelength. All measurements were performed in technical triplicates for each ligand concentration.

### Single-molecule force spectroscopy

RNA aptamer constructs (long stem, short stem, no stem) were synthesised with 5’ and 3’ overhangs for integration into the dumbbell assay^61^. DNA handles (0.5 kb) for tethering the target molecules to silica beads were PCR-amplified from λ-DNA, separated on a 2% (w/v) agarose gel, and purified to remove the side product using a QIAGEN Gel Extraction Kit. After agarose- and PAGE-gel purification, the aptamer and adaptor strands were mixed at a 1:1 molar ratio with dT-biotin- and dT-digoxigenin-labelled DNA handles (40 mM), respectively. The samples were dried in an Eppendorf Concentrator 5301, resuspended in folding buffer (1 M NaCl or 20 mM MgCl₂ in 50 mM HEPES, pH 7.8) and annealed using temperature-cycling protocols at 65 and 63 °C. The assembled constructs were verified by agarose gel electrophoresis^49,62^. The complete construct for measurement, consisting of the sample RNA, a single stranded adaptor strand, and handles, was assembled in two hybridisation steps following Walbrun et al^62^. In the first step, biotin-functionalized handles were hybridised to the ssDNA adaptor, while digoxigenin-functionalized handles were directly hybridised to the sample RNA. Handle DNA was diluted to 0.071 µM, mixed with the target molecule in a 1:1 molar ratio, and annealed. In the second step, products from the first hybridisation were combined and incubated for 1 h at 45 °C, 1 h at 35 °C and 4 h at 25 °C. The resulting constructs were verified via agarose gel electrophoresis (1.5%, TAE buffer) and stored at −80 °C. The final dumbbell construct was obtained by connecting the hybridised molecule to anti-digoxigenin- and streptavidin-functionalized micron-sized silica beads, which were trapped by two focused laser beams (Fig. 5a).

To minimise the chance of multiple molecules binding to the same bead pair, assembled constructs were diluted to ~0.1 nM and incubated for 10 mi at room temperature with micron-sized streptavidin-coated (SV) beads (Bangs Laboratories, Inc.) in the Optical Tweezer (OT) measurement buffer (40 mM HEPES, 5 mM MgCl₂, 250 mM KCl, 20 mM NaCl, pH 7.4). The mixture was then diluted into 300 µl of the same buffer. Similarly, anti-digoxigenin-coated (AD) beads were diluted to the same concentration.

In parallel, two measurement channels were prepared, each containing 500 µl of the OT measurement buffer. One of these contained varying amounts of Doxycycline depending on the individual measurement. Additionally, a scavenger system (final concentrations: 26 U/ml glucose oxidase (Sigma-Aldrich) 17,000 U/ml catalase (SERVA), and 0.65% glucose (Sigma-Aldric) was present in the measurement channels as well as an RNase Inhibitor (Thermo-Fisher) with a final concentration of 0.1X.

Experiments were performed on a commercial microfluidic chip (C-Trap® Optical Tweezers–Fluorescence & Label-free Microscopy, LUMICKS) with channels separated by laminar flow (Fig. 5b). The prepared mixtures were loaded into the C-Trap® syringe pump. Laminar flow and channel separation were maintained by applying ~0.35 bar pressure to the syringes, producing a flow velocity of ~20 µm/s. In each bead channel, an SV bead (pre-incubated with the sample construct) and an AD bead were captured in separate optical traps.

Next, a molecular tether was formed between the two beads and single-tether formation was confirmed through a few stretch–relax cycles showing the molecule’s characteristic fingerprint. The tethered molecule was then either kept in the same channel or transferred between channels, depending on the experimental design. Force ramp traces were recorded at a pulling velocity of 500 nm/s if not stated otherwise. Passive mode experiments were performed where the trap distance was held constant to gain more detailed insights into the folding/unfolding and binding/unbinding dynamics (Fig. 5d).

All measurements were performed at trap stiffnesses between 0.25–0.40 pN/nm and a temperature of ~25 °C. Data acquisition was initially at 78.125 kHz, then down sampled by a factor of 3 before analysis.

#### Force ramp traces

For the force (F) versus extension (e) curves shown in Fig. 4c, measurements were performed in so-called constant-velocity mode, i.e. the distance between the traps was increased at a fixed speed until the molecule was fully unfolded, then decreased to allow the RNA aptamer to refold. A pulling speed of 500 nm/s was used. These traces served as molecular fingerprints, enabling identification of distinct conformational states during unfolding and refolding.

For modelling polymer elasticity in force ramp cycles, segments of the force–extension traces corresponding to folded RNA aptamer were fitted with the extensible worm-like chain (eWLC) model^83^.

4 $${F}_{{eWLC}}\left(e\right)=\frac{{k}_{B}T}{{p}_{{dsDNA}}}\left[\frac{1}{4}{\left(1-\frac{e}{{L}_{{dsDNA},{linker}}}+\frac{F}{K}\right)}^{-2}-\frac{1}{4}+\frac{e}{{L}_{{dsDNA},{linker}}}-\frac{F}{K}\right]$$

where *k*_*B*_*T* represents the thermal energy, p_dsDNA_ is the persistence length of the dsDNA linker, *L*_dsDNA,linker_ is its contour length and *K* is the elastic stretch modulus. When portions of the RNA were unfolded, the elastic response was modelled as an eWLC connected in series with a standard WLC^84^.

5 $${F}_{{WLC}}\left(e\right)=\frac{{k}_{B}T}{{p}_{{ssRNA}}}\left[\frac{1}{4}{\left(1-\frac{e}{{L}_{{ssRNA}}}\right)}^{-2}-\frac{1}{4}+\frac{e}{{L}_{{ssRNA}}}\right]$$

where *p*_ssRNA_ denotes the persistence length of the unfolded ssRNA and *L*_ssRNA_ its contour length. For fits involving unfolded ssRNA (eWLC in series with WLC), the values of *L*_ds_DNA,linker, p_ds_DNA and *K* were fixed to those obtained from the previously fitted folded state of the same force ramp trace. In addition, *p*_ssRNA_ was fixed to 0.9 nm during fitting. The increase in unfolded contour length between successive states, determined from the WLC fits, was used to calculate the number of base pairs that had opened.

#### Passive-mode traces

To more accurately identify intermediate states, determine their lifetimes, and map a detailed folding/refolding pathway, we conducted passive-mode experiments in which the distance between the optical traps was held constant. This setup enabled real-time observation of RNA fluctuations between multiple intermediate states near equilibrium (Fig. 5d). In the resulting force–time traces, higher forces correspond to more folded states and lower forces to more unfolded states, as folding shortens the construct and pulls the beads out of the trap, which increases the load. Each data point was assigned to a specific state using Hidden Markov Modelling (HMM)^63^. The resulting state-assigned time trajectories were used to calculate transition rates^85^ and to reconstruct the folding network (Fig. 5e).

#### Off-rates and on-rates determination

No-stem construct: To determine the off-rates of doxycycline binding to the no-stem aptamer construct, we analysed the HMM-assigned passive-mode traces. As illustrated in Figure S15a, doxycycline-bound states are distinguished by a considerably longer lifetime on the force level of I1. Because the mean lifetimes of the unbound I1 and bound BI1 states differ by more than an order of magnitude, doxycycline-bound events can be readily identified. The off-rate $({k}_{{off}})$ was calculated as the reciprocal of the average bound-state lifetime $\left\langle {\tau }_{{bound}}\right\rangle$:

6 $${k}_{{off}}=\frac{1}{\left\langle {\tau }_{{bound}}\right\rangle }$$

Measured off-rates (Figure S16b) showed no significant dependence on applied force; therefore, the zero-force off-rate ${k}_{{off},0}$ was approximated as the mean of the force-dependent values.

The apparent on-rate (*k*_*on*_) was obtained from the reciprocal of the average unbound-state lifetime, normalised by the doxycycline concentration [*D*]:

7 $${k}_{{on}}=\frac{1}{\left\langle {\tau }_{{unbound}}\right\rangle \cdot \left[D\right]}$$

Because the binding rate to the unfolded state is significantly lower than to I1, *k*_*on*_ decreases with increasing force (grey data points, Figure S16c), where the molecule spends more time unfolded. To extract the on-rate for the native folded I1 state, the calculation was adjusted to

8 $${k}_{{on},I1}=\frac{1}{\left\langle {\tau }_{{unbound}}\right\rangle \cdot \frac{{P}_{I1}}{{P}_{{unbound}}}\cdot \left[D\right]}$$

where ${P}_{I1}$ is the probability of being in state I1 and ${P}_{{unbound}}$ is the probability of being unbound. This adjusted on-rate $\left({k}_{{on},I1}\right)$ showed no force dependence (Figure S16c), and the zero-load value ${k}_{{on},I{{\mathrm{1,0}}}}$ was calculated as the average of the measured ${k}_{{on},I1}$. The dissociation constant at zero load *K*_*D,0*_ was then determined as

9 $${K}_{D,0}=\frac{{k}_{{off},0}}{{k}_{{on},I1,0}}$$

Short-stem construct: The kinetics of the short-stem construct involving the folded state were too slow to apply the passive-mode rate determination used for the no-stem construct. Instead, we performed force-jump experiments. In these experiments, the molecule was initially held at a low trap distance, producing a low force and maintaining the folded state. The trap separation was then rapidly increased. The unfolding profile revealed whether the aptamer had been bound or unbound before the jump: a short lifetime at the I1 level indicated an unbound state, whereas a long lifetime indicated a bound state (see Figure S17 for more details).

To measure off-rates, experiments were initiated in a channel containing a doxycycline concentration at least two orders of magnitude higher than the expected *K*_*D*_, ensuring a high probability of starting in the bound state. The construct was then rapidly transferred to a channel without doxycycline, and after a defined waiting period, a force jump was applied to assess whether the ligand remained bound to the folded aptamer. Two waiting times were tested ( ~ 50 s and ~100 s). This process was repeated, and for each jump, the lifetime of the state on the I1 level was recorded. The resulting lifetime distribution exhibited a double-exponential form. Because a brief delay ( ~ ms) is required to reach the final trap separation and a finite observation window is imposed, lower (T1) and upper (T2) cutoffs were incorporated into the double-exponential cumulative distribution function:

10 $${{CDF}}_{{dexp}}\left(t\right)=\frac{A\left({e}^{-{k}_{1}t}-{e}^{-{k}_{1}{T}_{1}}\right)+\left(1-A\right)\left({e}^{-{k}_{2}t}-{e}^{-{k}_{2}{T}_{1}}\right)}{A\left({e}^{-{k}_{1}{T}_{2}}-{e}^{-{k}_{1}{T}_{1}}\right)+\left(1-A\right)\left({e}^{-{k}_{2}{T}_{2}}-{e}^{-{k}_{2}{T}_{1}}\right)}$$

From the fit, the amplitude *A* was obtained, representing the fraction of bound events. Using the amplitude and the corresponding waiting time ${t}_{{wait}}$, the off-rate for the folded state of the short-stem construct was calculated from a single-exponential cumulative distribution function:

11 $${{{{\rm{CDF}}}}}_{\exp }({{{{\rm{t}}}}}_{{{{\rm{wait}}}}})=1-{{{{\rm{e}}}}}^{-{{{\rm{k}}}}({{{\rm{off}}}},{{{\rm{shortstem}}}})\cdot {{{{\rm{t}}}}}_{{{{\rm{wait}}}}}}$$

To measure the on-rates, similar jump experiments were performed. In this case, jumps were carried out in a single channel containing either 0.5 or 1 µM doxycycline. After a defined waiting time of 4, 7 or 10 s, the force was rapidly increased from a low level to a higher level to assess whether the ligand was bound prior to the jump. Following aptamer unfolding, the force was reduced again to allow the molecule to refold into its native I1 state, enabling doxycycline binding from the surrounding solution.

This cycle was repeated, and the dwell times on the force level of state I1 were recorded. The resulting distributions again followed a double-exponential form. The fit amplitude *A* corresponded to the fraction of jumps in which the ligand was still unbound after the waiting period. It can be assumed that the time it takes for the aptamer to refold is small compared to the waiting time, given the rapid refolding rate to state I1 even under high force (see Fig. 5d). Moreover, because the mean bound lifetime of doxycycline to the aptamer is at least an order of magnitude longer than the waiting time, the probability of binding and subsequently unbinding within the same interval is negligible.

Using the single exponential cumulative function described above, and normalising the rate by the ligand concentration, we determined the on-rate to the folded state of the short-stem construct (Table S10).

The dissociation constant at zero load ${K}_{D,0}$ for the short-stem construct was obtained by dividing the off-rate by the on-rate as described above. The values in Table S10 are reported as the mean value ± standard error of the mean determined by error propagation method.

### Stopped flow spectroscopy

The ligand capture process was monitored using a stopped-flow (SF) π*-180 device from Applied Photophysics (Leatherhead, UK) at 20 °C. The following parameters were applied: excitation wavelength (λ_ex_) = 375 nm, emission wavelength (λ_em_) > 380 nm, fluorescence detection mode at a 90° angle to the excitation source, photomultiplier tube (PMT) voltage = 450 V, acquisition of 10,000 data points (logarithmic spacing) over 50 sec, slit width = 1 nm, and pressure hold “on”. All measurements were conducted in SFS buffer (40 mM KP_i_, 200 mM NaCl, 20 mM MgCl_2_, pH 7.5) at an RNA concentration of 4 µM, with varying ligand concentrations of 8, 16, 24, and 32 µM (2, 4, 6, and 8 equivalents (eq)). Preliminary experiments (Figure S19c-d) confirmed a fully bound state under these conditions. The experimental dead time for each measurement was estimated to be ~150 µs. All time traces were subjected to baseline correction, averaging, smoothing (using a weighted moving average algorithm with a window size of 50 points) and normalisation (to a range of 0 to 1). Kinetic modelling (KM) analysis of the normalised ligand-dependent SF data was carried out using the DynaFit4 software^86^. A more comprehensive explanation of the data processing methodology and the various binding models employed for analysis can be found in the Supplementary Information.

### Reporting summary

Further information on research design is available in the Nature Portfolio Reporting Summary linked to this article.

## Supplementary information

Supplementary Information

Reporting Summary

Transparent Peer Review file

## Source data

Source Data

## Data Availability

All experimental data generated in this study are provided in the Supplementary Information/Source Data files. The NGS data generated in this study have been deposited in the European Nucleotide Archive (ENA) under the accession code PRJEB121469 and on the Zenodo database under accession code 20424385 the NGS data are available with the code used for analysis. Source data are provided with this paper.

## Code availability

All code and bioinformatic data used in this manuscript is publicly available on Zenodo under accession code 20424385 (https://zenodo.org/records/20424385; DOI: 10.5281/zenodo.20424385).
