# Supplementary material for: Mechanism underlying the high regulatory performance of the doxycycline riboswitch G12
Source: Nat Commun. 2026 Aug 3;17:7697. doi: 10.1038/s41467-026-76256-2 (PMC13434769; doi:10.1038/s41467-026-76256-2)
Supplement: Supplementary file 1 [file 41467_2026_76256_MOESM1_ESM.pdf]

SUPPLEMENTARY INFORMATION

Mechanism underlying the high regulatory performance of the doxycycline riboswitch G12

Hoetzel, J.[1], Walbrun, A.[2], Schäfer, M.[1], Wang, T.[1], Jørgensen, A.G.[3], Becker, O.[1], Stamatakis, K.[4], Gunawan, V.[1], Reichardt, L.[2], Boettger, L.[1], Bruckhoff, R.W.[1], Kjems, J.[3], Wachtveitl, J.[4], Rief, M.[2], Suess, B.[1,5*]

[1] Department of Biology, Technical University of Darmstadt, Darmstadt, Germany

[2] TUM School of Natural Sciences, Department of Bioscience, Center for Functional Protein Assemblies (CPA), Technical University of Munich, Garching, Germany

[3] Interdisciplinary Nanoscience Center, Aarhus University, Aarhus, Denmark

[4] Institute for Physical and Theoretical Chemistry, Goethe-University at Frankfurt, Frankfurt am Main, Germany

[5] Centre for Synthetic Biology, Technical University of Darmstadt, Darmstadt, Germany

*Correspondence: bsuess@bio.tu-darmstadt.de

Table of content

Supplementary Information Box p.1

Supplementary Figures

Figure **S1**	Pool design & in vivo screening	p. 5
Figure **S2**	Cytometry data of pre-sorting prior to in vivo screening	p. 7
Figure **S3**	Representative cytometry data of in vivo screening in yeast	p. 8
Figure **S4**	Reads attributed to identified sequence motifs and their clustering	p. 9
Figure **S5**	Mutational studies on the aptamer G12	p. 10
Figure **S6**	Representative cytometry data of rational mutations of G12	p. 12
Figure **S7**	Cytometry data of the randomised mutagenesis screening of G12_M3	p. 13
Figure **S8**	Structural insights by SHAPE-MaP	p. 14
Figure **S9**	K_D measurements of the aptamer G12 and its variants M3 and S1	p. 15
Figure **S10**	Fluorescence titration spectroscopy of rational mutations of G12	p. 17
Figure **S11**	Testing binding of the aptamer G12 and its variants M3 and S1 to tetracycline and magnesium dependence of G12 for the binding to doxycycline	p. 18
Figure **S12**	Fluorescence titration spectroscopy of G12 with doxycycline derivatives	p. 20
Figure **S13**	Test of G12-based splice-regulator controlling GFP expression in human cells	p. 21
Figure **S14**	Test of ligand specificity of G12 in human cells	p. 22
Figure **S15**	Comparison of G12 and the tetracycline aptamer in force-ramp experiments	p. 24
Figure **S16**	Determination of doxycycline ON and OFF rates measured by SMFS	p. 26
Figure **S17**	Determination of doxycycline ON and OFF rate for the short stem construct of G12 using single-molecule force-jump experiments	p. 27
Figure **S18**	Effect of Mg^{2+} on aptamer folding and doxycycline binding	p. 28
Figure **S19**	Initial fluorescence titration and circular dichroism thermal melting of G12	p. 29

Supplementary Methods p. 30

Supplementary Tables

Table **S1**	Selection parameters and elution fractions of the column-SELEX	p. 31
Tabel **S2**	Selection parameters and elution fractions of the Capture-SELEX	p. 32
Table **S3**	Cytometer data from the cell-sorting prior to *in vivo* screening in yeast	p. 33
Table **S4**	Regulatory performance of G12 mutants tested in yeast	p. 34
Table **S5**	Cytometer data of the randomised mutation screening in yeast	p. 35
Table **S6**	Dose dependency of the regulatory activity of G12 and its variants in yeast	p. 36
Table **S7**	Regulatory potential of different synthetic riboswitches in yeast	p. 37
Table **S8**	Cytometer data of GFP/mCherry measurements in HEK293 cells	p. 38
Table **S9**	Contour-length differences between intermediate states of the long-stem, short-stem and no-stem G12 aptamer constructs as determined by SMFS	p. 39
Table **S10**	Affinities and rate constants for doxycycline binding to the G12 aptamer measured by SMFS	p. 40
Table **S11**	Unfolding rate analysis via single-molecule force-ramp measurement	p. 41
Table **S12**	List of all 24 tested models during the comparative analysis of the ligand-binding kinetics	p. 42
Table **S13**	Summary of the comparative analysis of the ligand binding kinetics of G12	p. 43
Table **S14**	List of determined melting points of the CD thermal melting assay	p. 44
Table **S15**	Oligonucleotide sequences	p. 45
Table **S16**	Plasmid sequences	p. 49

Supplementary Information Box

Column-SELEX *vs.* Capture-SELEX: Operational guidelines and decision-making framework

Within this information box, detailed information about the *in vitro* selection process (SELEX) is provided. The two methods applied are described in detail and compared with one another. The box includes a detailed workflow including a table that directly compares both methods (pg.2). This is followed by a table discussing respective biases and considerations (pg. 3), followed by a practical decision framework – when to use Capture- and when column-SELEX? (pg. 4)

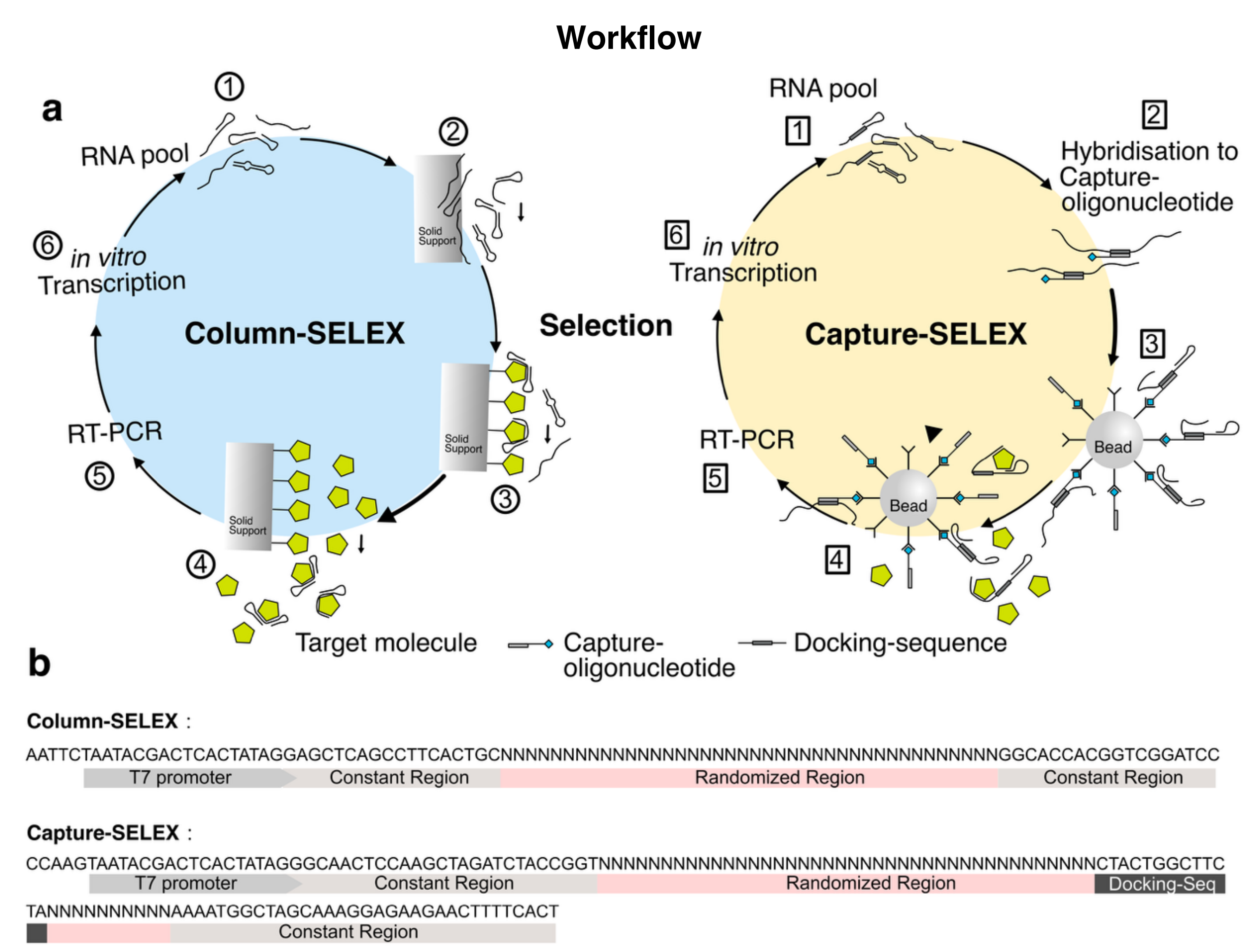

Comparison of Column-SELEX and Capture-SELEX workflows. a) In both approaches ①/[1] a randomised DNA library (commercially purchased) is transcribed *in vitro* into an RNA pool (about 10^{15} RNA molecules). For Column-SELEX, a pre-selection step ② is performed to remove sequences with strong nonspecific affinity for the solid support (e.g. Sepharose). The remaining RNA pool is then incubated with a target molecule (green pentagon) which is immobilised on the column matrix ③, enabling retention of high-affinity binders. Bound aptamers are subsequently eluted using free ligand (specific elution), alternatively non-specific elution is also feasible (see below) ④. For Capture-SELEX, the RNA pool is first hybridised to biotinylated DNA capture oligonucleotides [2]. The resulting complexes are immobilised on streptavidin-coated magnetic beads via biotin–streptavidin interaction [3]. Upon addition of the target molecule (green pentagon) [4], ligand binding leads to the dissociation of some RNAs from the capture oligonucleotides. For both methods, the eluted RNA sequences are reverse transcribed into cDNA ⑤/[5] and amplified by PCR. The enriched DNA pool is then *in vitro* transcribed into RNA ⑥/[6], and the selection cycle is repeated to progressively enrich high-affinity aptamers. **b**) Examples of pool designs for the two SELEX methods. A pool used for Column-SELEX is only constrained by the need for constant regions, enabling its reverse transcription and PCR amplification after each selection step. The randomised region can be freely adjusted to fit the selection aims (e.g., with partially fixed sequences). The Capture-SELEX pool contains a docking-sequence that is necessary for the immobilisation of the RNA pool and restricts pool design. **Note:** In the present study, the Capture-SELEX pool has been used for both Column- and Capture-SELEX for comparability.

Workflow: Direct comparison Column/Capture-SELEX

Steps	Procedure	Column-SELEX	Capture-SELEX
①/[1]	**RNA Pool Design**	**Pool Design:** The library consists of a continuous random region flanked by two constant regions. The length of the randomised region can be chosen freely; it is typically 25–70 nt (Fig. S18 b).	**Docking-Constrained Design:** The random region (about 50 nt) incorporates a fixed docking sequence (e.g., 13 nt) designed to hybridise with a complementary capture oligonucleotide (biotinylated single-stranded) (Fig. S18 b).
②/[2]	**Pre-selection / Hybridisation**	The *in vitro* transcribed aptamer pool is incubated with the solid support (e.g., Sepharose) to remove aptamers with unspecific binding affinity.	The *in vitro* transcribed aptamer pool is hybridised to the biotinylated capture oligonucleotide.
③/[3]	**Target/Pool Immobilisation**	**Target Immobilisation:** The target molecules are covalently immobilised onto a solid support. The RNA pool flows through the column in the liquid phase, allowing binding aptamers to attach while non-binders are washed away.	**Library Immobilisation:** The RNA pool is immobilised onto magnetic beads via the capture oligonucleotide. The target molecules are introduced in the liquid phase and diffuse freely without the necessity for chemical modification.
④/[4]	**Elution Mechanism**	**Affinity Elution:** Bound aptamers are eluted by introducing a high concentration of free target molecules, which competitively displace the aptamers from the immobilised targets. **Unspecific Elution:** Heat or chaotropic agents can be used to unspecifically elute.	**Allosteric Elution:** Addition of target molecules causes dissociation of RNAs from the capture oligonucleotide and elute into the supernatant.
⑤/[5]	**Recovery & Amplification**	The eluted RNA pool is precipitated, reverse transcribed into cDNA and subsequently amplified via PCR.	The supernatant containing the eluted RNA pool is precipitated, reverse transcribed into cDNA and amplified via PCR.
⑥/[6]	**Regeneration**	The amplified DNA serves as a template for *in vitro* transcription to generate the RNA pool for the subsequent selection round.	

Biases and Considerations

Parameters	Column-SELEX	Capture-SELEX
**Library Constraints**	**Low**, the randomised region can be designed without any limitations and provides complete structural diversity, allowing for structural complex aptamers	**Moderate**, the required docking sequence of the capture oligonucleotide restricts folding potentials, which may limit the overall structural diversity.
**Target Requirements**	May require targets with available functional groups (e.g., -NH2, -COOH) for immobilisation. Immobilisation must not mask critical binding epitopes.	Unlimited, highly suitable for small molecules, even some hydrophobic targets, or targets lacking functional groups, as they remain in their native state in solution.
**Elution Specificity**	Relies on affinity competition. High concentrations of free targets may elute non-specific binders or aptamers with moderate affinity.	Relies on target-induced dissociation, favouring conformational changes.
**Anticipated Biases**	Tends to enrich aptamers with the highest absolute binding affinity (K_a) and slower dissociation rates (k_{off}).	Tends to enrich aptamers that are sensitive to ligand binding and possess flexible, structure-switching conformations.
**Failure Modes**	Target immobilisation may alter its chemical identity or mask potential epitopes. Incomplete elution of high affinity binders may occur.	Target molecules and agents to solve them can lead to unspecific dissociation of RNA. Ligand-RNA interactions can be insufficient to dissociate the RNA from the capture oligonucleotide.

Practical Decision Framework - How to choose?

The selection between Capture-SELEX and column-SELEX should be guided by the nature of the target molecule, the desired properties of the resulting aptamer and the intended downstream applications of aptamer. Capture-SELEX tends to enrich aptamers that undergo conformational change upon binding of the ligand, providing the prerequisite to be further engineered into synthetic riboswitches for gene regulation. In contrast, Column-SELEX allows maximal freedom in pool design since the method does not require immobilisation of the pool. This leaves out the necessity of a docking sequence like in the Capture-SELEX and allows to freely use fully or partly randomised sequences for the selection. Yet, this method only selects for binding to the ligand, lowering the chances of identifying an aptamer that is suited for riboswitch engineering.

When to choose column-SELEX?

- Easy to immobilise target: The target molecule is relatively large or possesses accessible functional groups that allow for covalent immobilisation without significantly altering its spatial conformation or masking binding epitopes.
- Pursuit of binding affinity: The primary objective is to isolate aptamers with the lowest possible dissociation constant (KD), instead of their structural flexibility or suitability for specific switch architectures.
- Easy library design: A simple library design without internal fixed sequences is preferred to maximise sequence and structural diversity.

When to choose Capture-SELEX?

- Small molecule targets: The target is a relatively small molecule (e.g., antibiotics) that is difficult to immobilise or whose binding sites would be completely obscured upon immobilisation.
- Towards biosensor or gene regulation application: The intended downstream application requires aptamers capable of conformational switching (e.g., synthetic riboswitch, electrochemical or fluorescent biosensing assays). Capture-SELEX inherently selects for this functional property.
- Native liquid-phase dynamics: The target molecules can remain in its native, unmodified state in solution, thereby avoiding any solid-phase surface effects.

Supplementary Figures

Figure S1

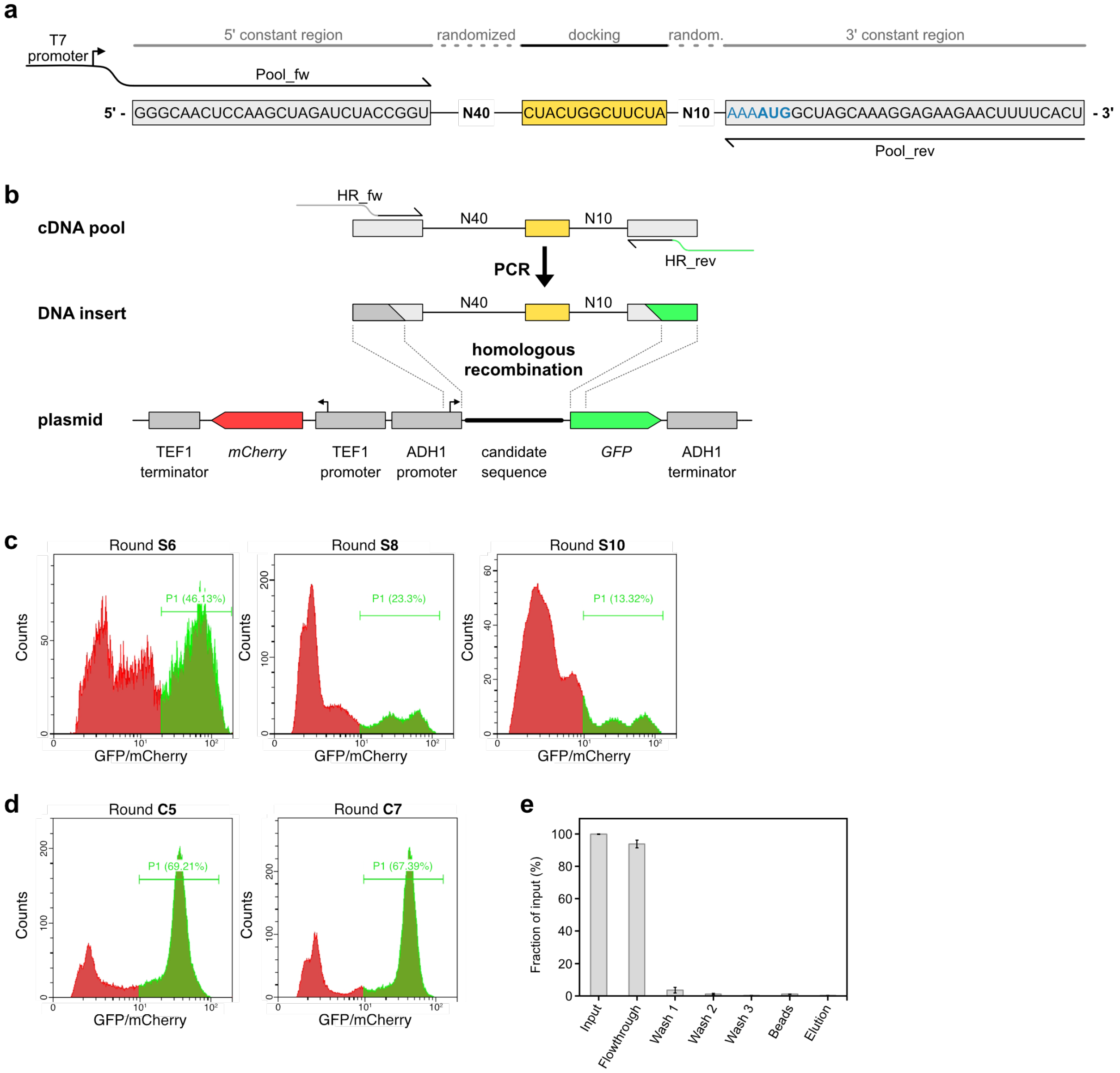

Figure S1: Pool design & *in vivo* screening. a) Design of the Capture-SELEX pool. The pool consisted of a 5' and 3' constant region (grey), a N40 and N10 randomized region and the docking sequence (yellow) for hybridisation to the capture-oligonucleotide separating the randomized regions. The 3' constant region contains the yeast-optimised Kozak sequence (blue) for subsequent *in vivo* screening in yeast. Primer used for RT-PCR are indicted. The forward primer added the sequence of the T7 promoter to allow *in vitro* transcription of the pool for the next selection round. **b**) Schema of the procedure for the insertion of the SELEX derived pools into pCBB06 for subsequent *in vivo* screening in yeast. PCR was performed with the DNA pool obtained from SELEX to add overhangs for the homologous recombination. The PCR product was then brought into yeast with the digested plasmid pCBB06 by electroporation. After successful homologous recombination the aptamer candidate was positioned in the 5'UTR of the GFP reporter gene. **c**) Pre-selection of aptamer candidates from rounds S6, S8 and S10 obtained from column-SELEX. Selected were viable cells with strong mCherry expression and a good GFP expression (P1, green area). The proportion of selected cells from all measured events is shown in percent. **d**) Pre-selection of aptamer candidates from rounds C5 and C7 obtained from Capture-SELEX. Selected were viable cells with strong mCherry expression and a good GFP expression (P1, green area). The proportion of selected cells from all measured events is shown

in percent. **e**) Test of the aptamer G12 in the Capture-SELEX setup. Bars depict the measured radioactive signal from the ^{32}P-labeled RNA. The candidate G12 was almost completely washed of the beads in this selection, as represented by the high fraction of RNA in the flowthrough. Remaining RNA was removed by the following washing steps, resulting in no RNA recovered in the elution step with doxycycline. This direct loss of the whole RNA in the flowthrough and washing steps indicates that the RNA has not hybridized to the capture-oligonucleotide since it was not immobilised on the beads.

Figure S2

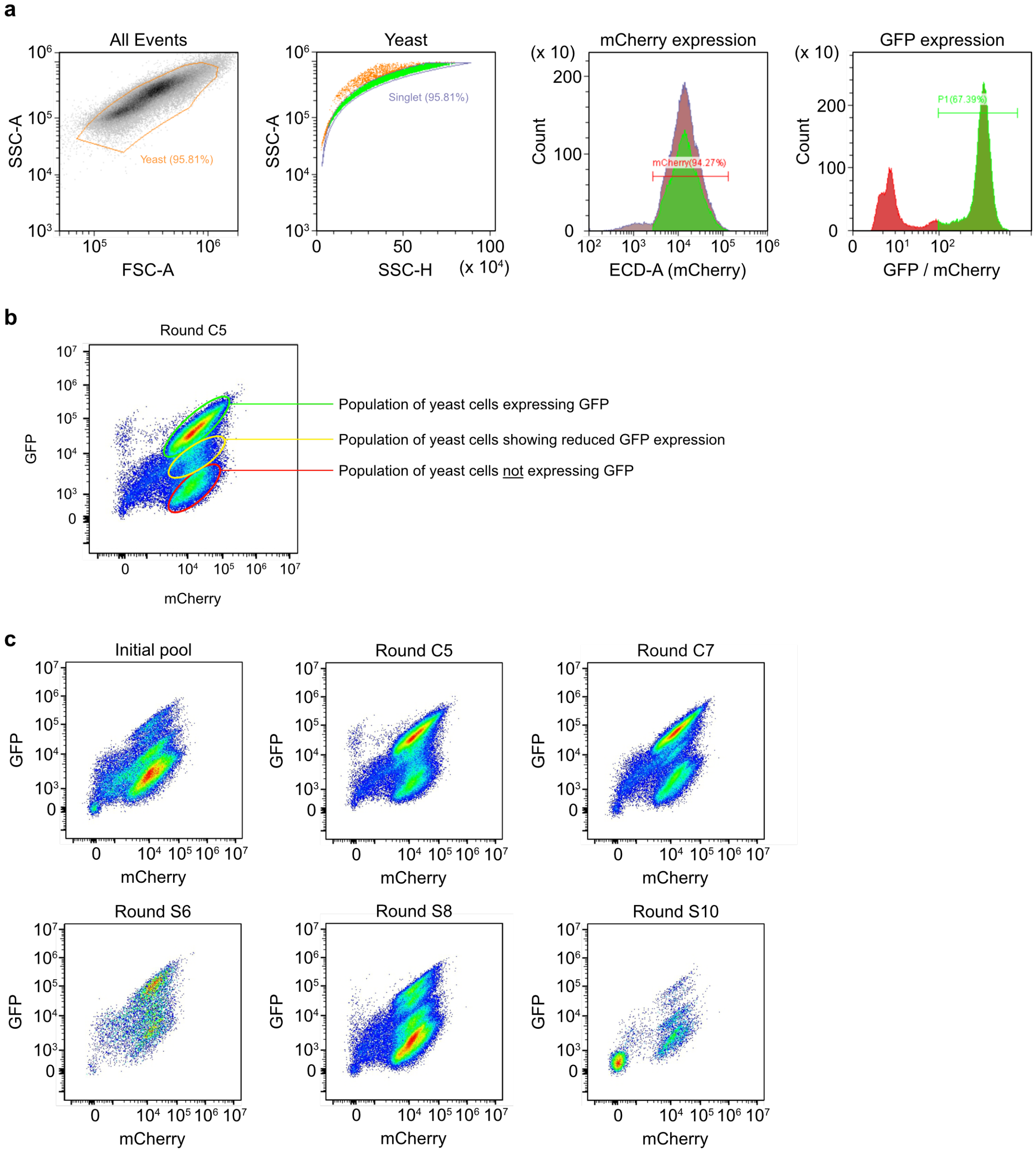

Figure S2: Cytometry data of the pre-sorting prior to *in vivo* screening. a) General gating strategy shown on the example of round C7. The first gate was set to contain yeast cells. The second gate was set to only contain yeast singlets. The third gate was set to include yeast showing sufficient mCherry expression, indicating healthy protein expression. The last gate was only used for the pre-sorting before the *in vivo* screening, set to contain cells that showed a sufficient level of GFP expression normalised by the constitutive mCherry expression. **b**) Example of the shown cytometer data of GFP and mCherry expression in yeast. Reporter expressions are plotted with mCherry fluorescence shown on the x-axis and GFP expression shown on the y-axis. Reduced expression of GFP (under control of the aptamer) upon doxycycline addition manifests in signal decrease on the x-axis, whereas mCherry (as the constitutive expressed control) is not expected to change in fluorescence. Different levels of GFP expression are indicated (high, reduced, no expression). **c**) Data from the initial pre-sorting of the five rounds obtained from the two SELEX methods before *in vivo* screening in yeast. As a reference, the initial (unselected) pool was also inserted into pCBB06 and sorted. The pools were sorted for cells expressing GFP in the absence of doxycycline, to subsequently only test clones that are able to express GFP.

Figure S3

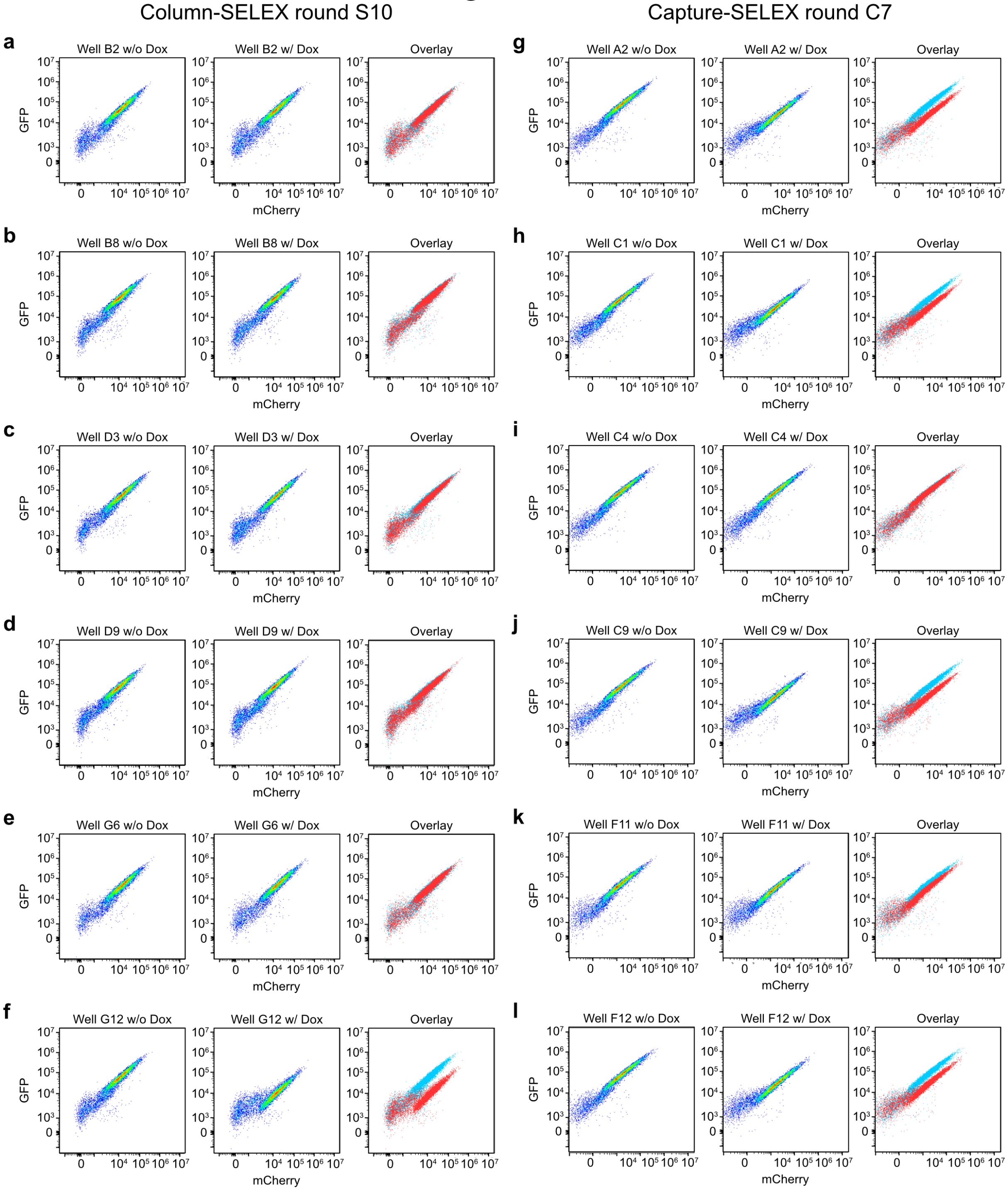

Figure S3: Representative cytometry data of the *in vivo* screening in yeast. Shown are the cytometer data of representative candidates (named after their wells) obtained from round S10 from Column-SELEX **a**) B2 **b**) B8 **c**) D3 **d**) D9 **e**) G6 **f**) G12 and from round C7 from Capture-SELEX **g**) A2 **h**) C1 **i**) C4 **j**) C9 **k**) F11 **l**) F12. The first plot in each panel shows the mCherry and GFP expression in the absence of doxycycline, the second shows the expression in the presence of doxycycline and the third shows the overlay of the first two. Note that the only candidate from Column-SELEX showing notable regulatory response to addition of doxycycline is G12, shown in panel f).

Figure S4

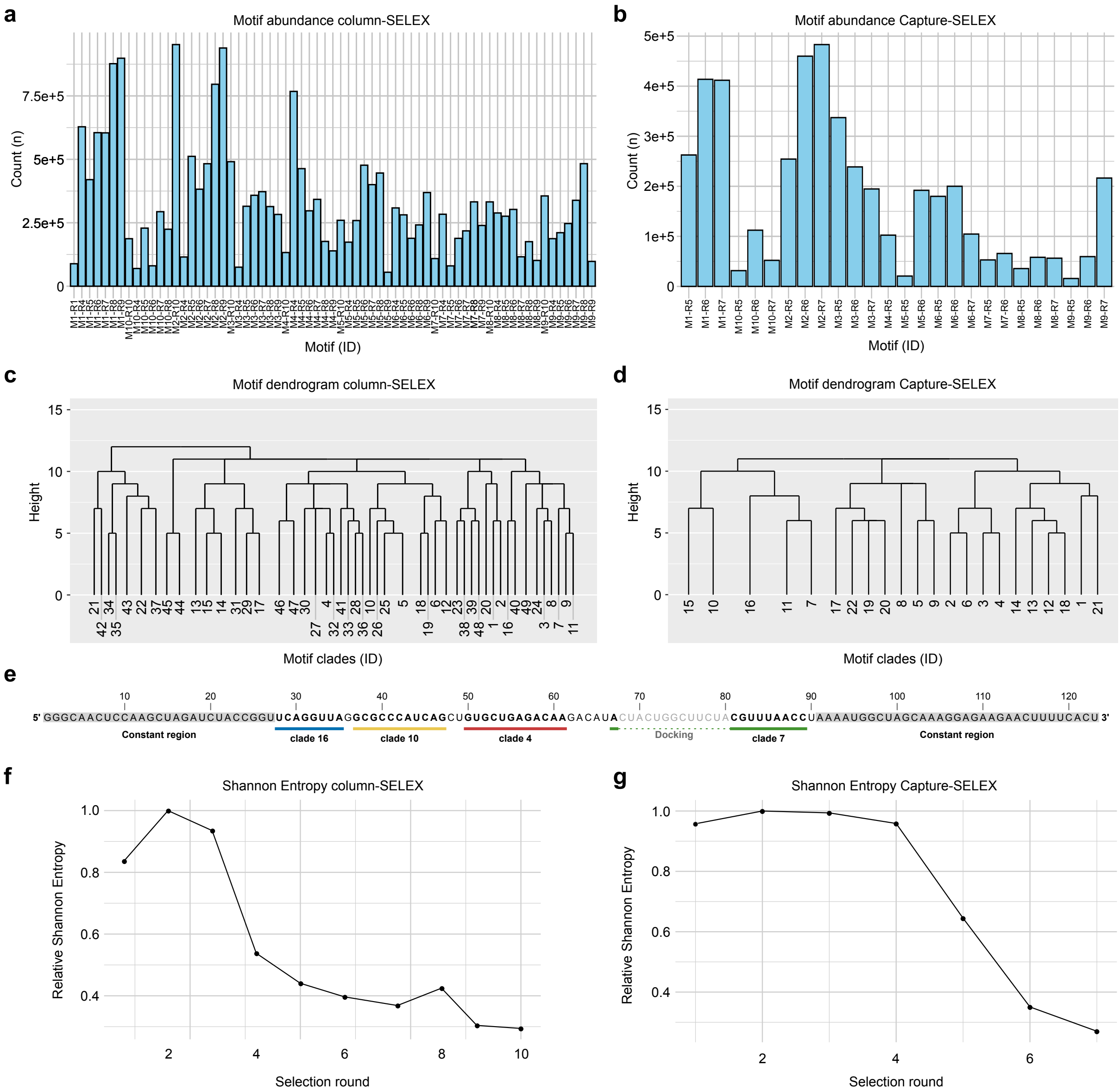

Figure S4: Reads attributed to identified sequence motifs and their clustering to clades. a) Read counts identified for every identified motif in the column-SELEX. **b**) Read counts identified for every identified motif in the Capture-SELEX. **c**) Motif dendrogram of the motif clustering based on FIMO resulting in the motif clades 1 to 49 from the column-SELEX. **d**) Motif dendrogram of the motif clustering based on FIMO resulting in the motif clades 1 to 22 from the column-SELEX. **e**) Mapping of motif clades found in round C7 to the sequence of C01. Letters with grey background indicate the constant regions of the SELEX pool and letters in grey indicate the docking sequence. **f**) Relative Shannon entropy of the individual selection rounds of the column-SELEX. **g**) Relative Shannon entropy of the individual selection rounds of the Capture-SELEX.

Figure S5

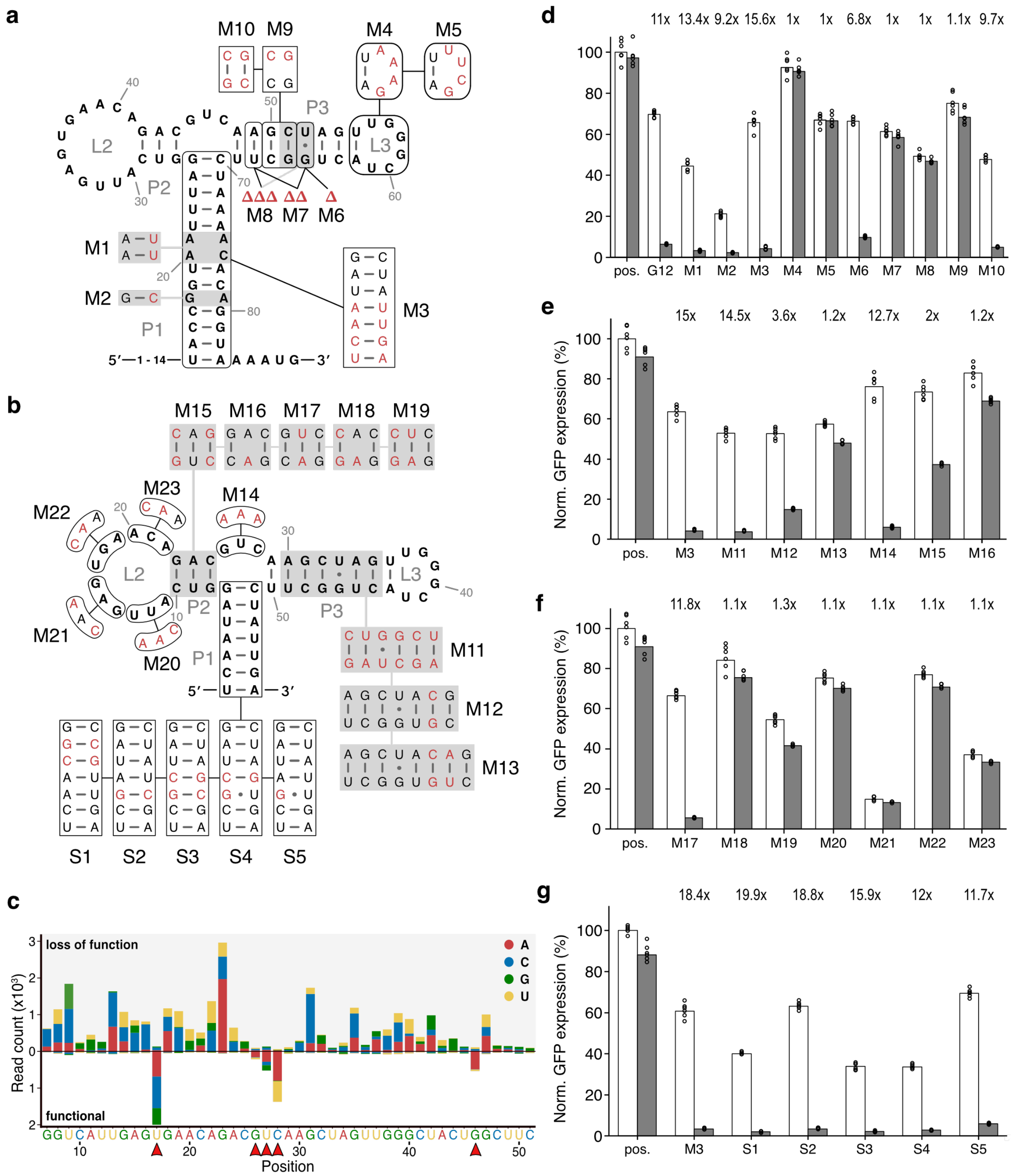

Figure S5: Mutational studies on the aptamer G12. a) Depiction of the predicted 2D structure (RNAfold) of G12 with the rational designed mutations tested on the aptamer. Highlighted in red are the changes introduced by the mutations. **b**) Depiction of the predicted 2D structure (RNAfold) of M3 with the rational designed mutations tested on the aptamer. Highlighted in red are the changes introduced by the mutations. **c**) Single mutant reads found in the randomized mutations screening mapped to their effect on the regulatory function and the original sequence of M3. Colours of the stacked bars indicate the changed base found at the respective position. Mutations leading to loss of regulatory behaviour are mapped as “loss of function” and mutants remaining regulatory function similar to M3 are mapped as “functional”. Positions allowing nucleotide variability without loss of regulation are highlighted by red arrow heads. **d**) GFP expression levels of G12 and its mutants M1-M10 normalized to mCherry and relative to the positive control pCBB05 without doxycycline (white bars) and with 100 μM doxycycline

(grey bars). Error bars indicate standard deviation calculated from two independent measurements with biological triplicates. **e**) GFP expression levels of G3 and its mutants M11-M16 normalized to mCherry and relative to the positive control pCBB05 without doxycycline (white bars) and with 100 μM doxycycline (grey bars). Error bars indicate standard deviation calculated from two independent measurements with biological triplicates. **f**) GFP expression levels of the mutants M17-M23 normalized to mCherry and relative to the positive control pCBB05 without doxycycline (white bars) and with 100 μM doxycycline (grey bars). Error bars indicate standard deviation calculated from two independent measurements with biological triplicates. **g**) GFP expression levels of stem variants of G12 S1-S5 normalized to mCherry and relative to the positive control pCBB05 without doxycycline (white bars) and with 100 μM doxycycline (grey bars) in comparison to M3. Error bars indicate standard deviation calculated from two independent measurements with biological triplicates. All experiments in yest were performed in two independent measurements in triplicates (n = 6). Representative cytometric raw data of rational designed mutations are shown in Figure S6.

Figure S6

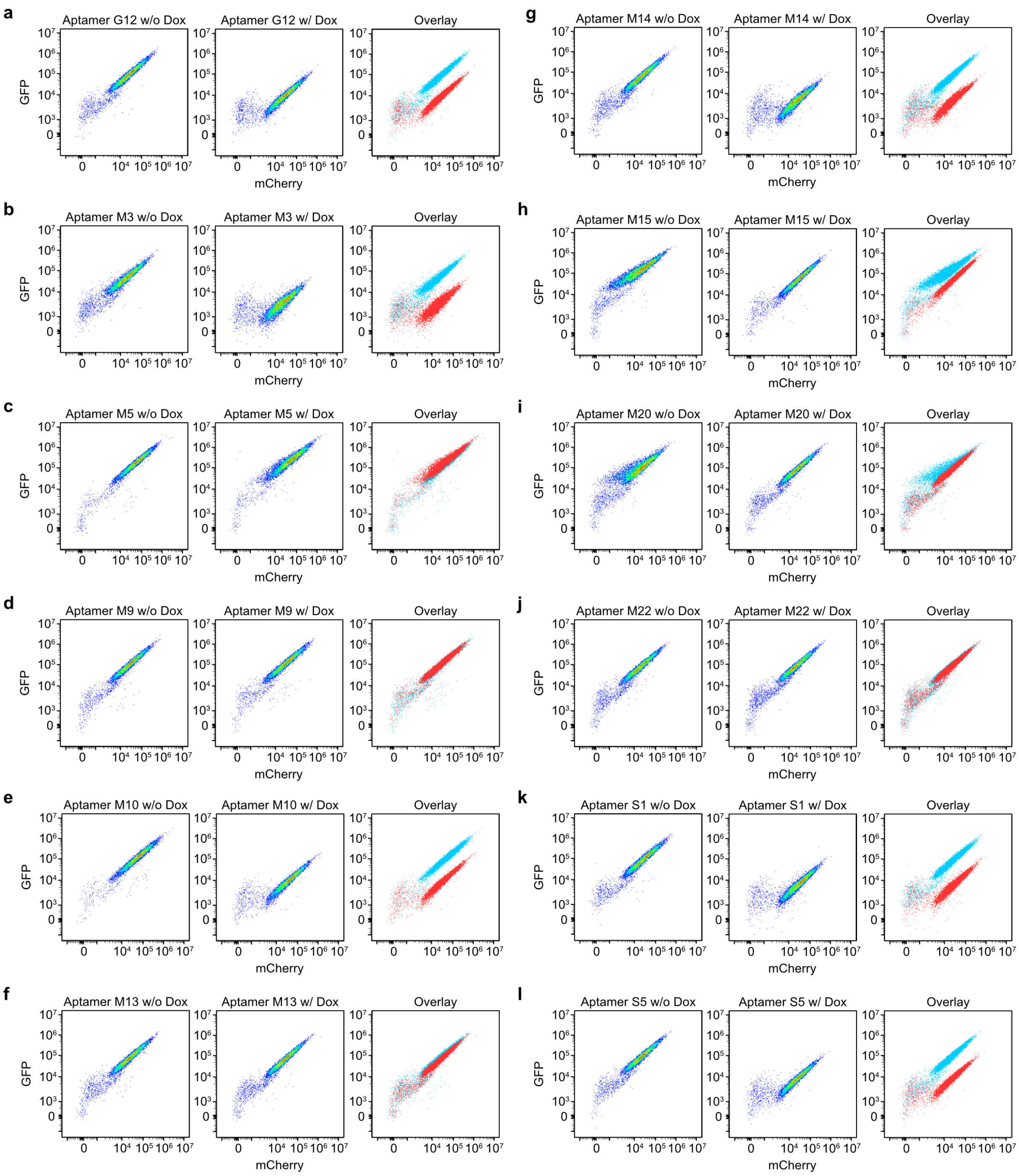

Figure S6: Representative cytometry data of rational mutations of G12. Shown are representative cytometer data of variants of the doxycycline aptamer G12 in yeast: **a**) The unaltered aptamer G12 as obtained from the *in vivo* screening, **b**) mutation M3, **c**) mutation M5, **d**) mutation M9, **e**) mutation M10, **f**) mutation M13, **g**) mutation M14, h) mutation M15, i) mutation M20, j) mutation M22, k) P1 stem variant S1 and **l**) P1 stem variant S5. The first plot in each panel shows the mCherry and GFP expression in the absence of doxycycline, the second shows the expression in the presence of doxycycline and the third shows the overlay of the first two. The response in reduced GFP expression upon addition of doxycycline is clearly visible for G12, M3, M10, M14, M15, S1 and S5, whereas M5, M9, M13, M20 and M22 show no response to the addition of the ligand.

Figure S7

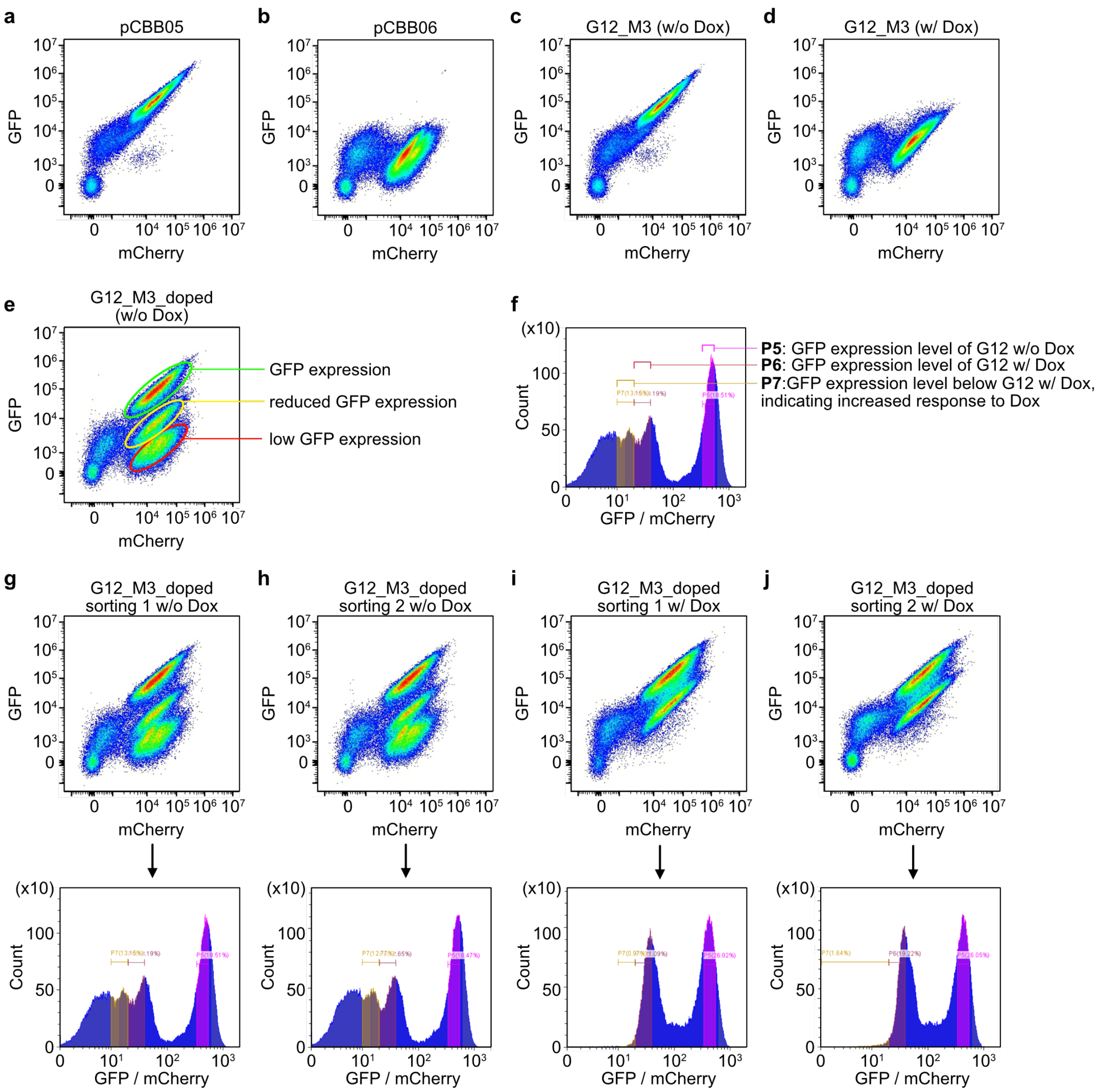

Figure S7: Cytometry data of the randomised mutagenesis screening of G12_M3. a) Positive control pCBB05 showing high and constitutive GFP expression. **b**) Negative control pCBB06 showing very low GFP fluorescence, due to the lack of the start codon in the GFP coding sequence. **c**) The unaltered aptamer G12_M3 shows high GFP expression in the absence of doxycycline and **d**) a strong reduction of GFP upon addition of doxycycline. **e**) Expression of the pool of randomly mutated G12_M3 in yeast. The pool displays a range of high, reduced and low to no GFP expression in the absence of doxycycline. **f**) Schema of the gates set to select the mutations from the randomly mutated pool for subsequent sequence and classification of the mutations to the categories of functional or non-functional. Gate P5 was used for the sorting in the absence of doxycycline to select for the mutations allowing GFP expression. Gates P6 and P7 were used in the following sorting step to select the variants that showed reduced GFP expression in the presence of doxycycline, while gate P5 was used to select variants that were unable to reduce GFP expression upon doxycycline addition. First **g**) and second **h**) sorting in the absence of doxycycline. First **i**) and second **j**) sorting in the presence of doxycycline.

Figure S8

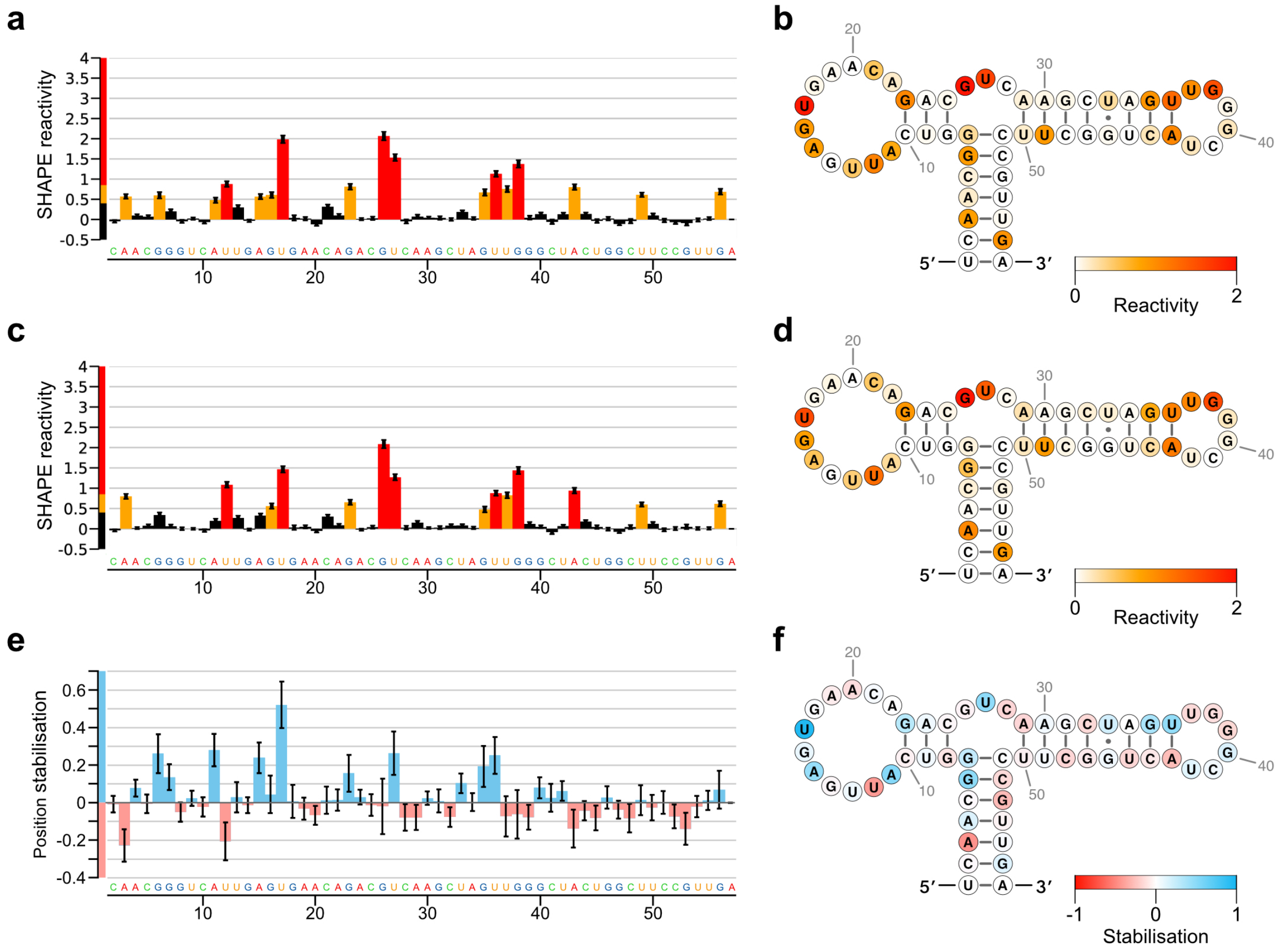

Figure S8: Structural insights by SHAPE-MaP. a) SHAPE reactivity nucleotide positions of G12_S1 in the absence of doxycycline. The normalized reactivity of individual nucleotide positions (x-axis) is shown on the y-axis and indicated by colour as defined by the standard settings of ShapeMapper 2 (orange 0.4 – 0.85, red >0.85). **b**) Normalized reactivity in the absence of doxycycline mapped to the secondary structure of G12_S1. Reactivity is indicated by colour, with the highest reactivity represented by dark red. **c**) SHAPE reactivity nucleotide positions of G12_S1 in the presence of doxycycline. The normalized reactivity of individual nucleotide positions (x-axis) is shown on the y-axis and indicated by colour. **d**) Normalized reactivity in the presence of doxycycline mapped to the secondary structure of G12_S1. Reactivity is indicated by colour, with the highest reactivity represented by dark red. **e**) Changes in reactivity of nucleotide positions of G12_S1 calculated from the position reactivity with and without doxycycline. Stabilisation was defined as reduced reactivity in the presence of doxycycline compared to the reactivity in the absence of doxycycline. The calculated stabilisation or destabilisation effects of individual nucleotide positions (x-axis) is shown on the y-axis and indicated by colour (red = destabilisation, blue = stabilisation). **f**) Calculated position stabilisation in the presence of doxycycline mapped to the secondary structure of G12_S1. Reactivity is indicated by colour, with stabilisation in blue, destabilisation in red and no change in nucleotide reactivity in white.

Figure S9

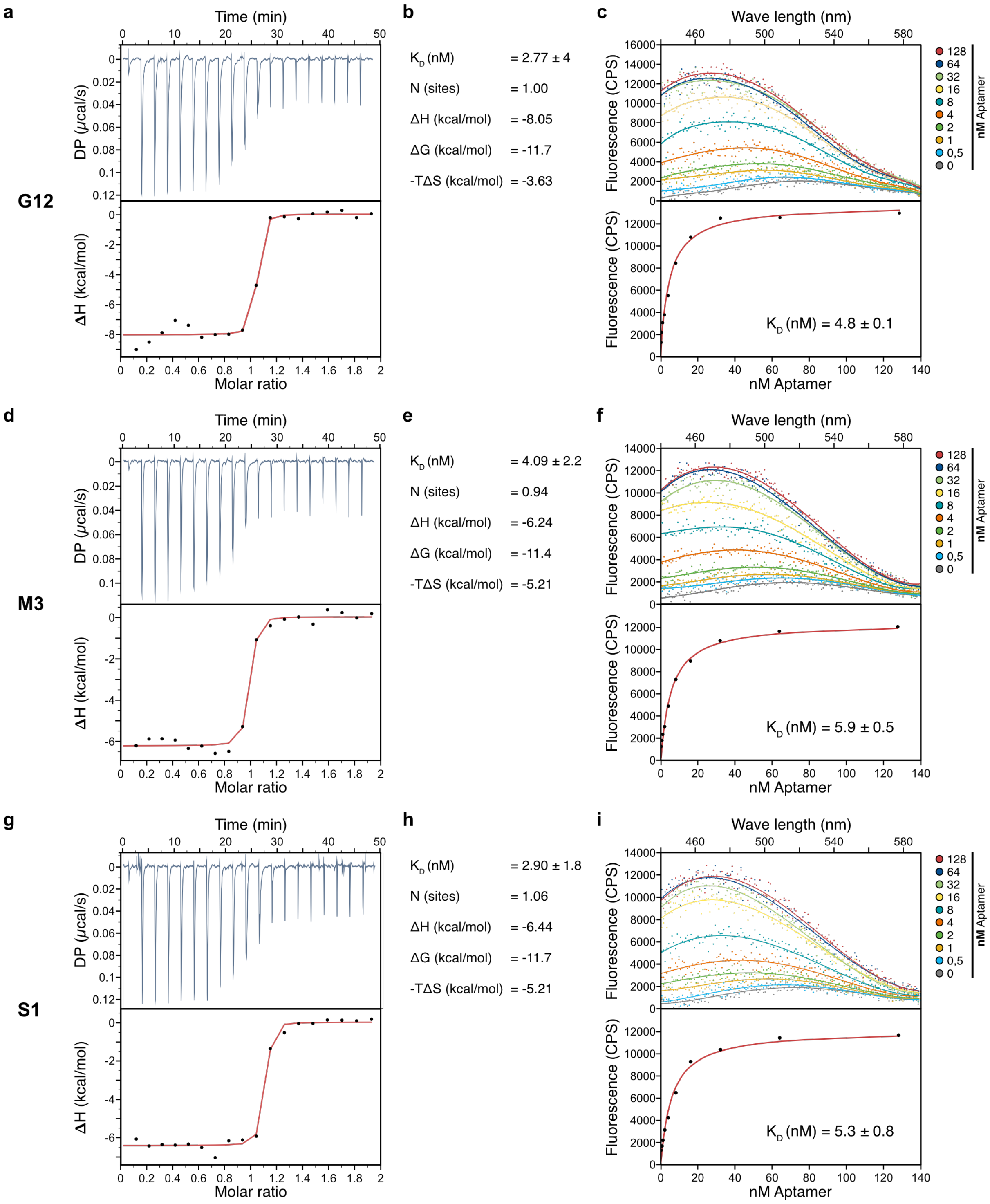

Figure S9: K$_D$ measurements of the aptamer G12 and its variants M3 and S1. a) Titration curve and thermogram of a representative ITC experiment measuring binding of G12 to doxycycline. Black dots depict the experimental data while the red line represents the fitted binding curve. **b**) Results of the ITC experiment measuring binding of G12 to doxycycline. **c**) Results of FTS measuring fluorescence of doxycycline when binding to G12. Shown are the measured fluorescence at different aptamer concentrations and the fluorescence levels at 490 nm wavelength used for calculation of the K$_D$. The calculated K$_D$ of the respective measurement is given below the curve. Black dots depict the experimental data while the red line represents the fitted Hill equation. **d**) Titration curve and thermogram of a representative ITC experiment measuring binding of the G12 variant M3 to doxycycline. **e**) Results

of the ITC experiment measuring binding of M3 to doxycycline. **f**) Results of FTS measuring fluorescence of doxycycline when binding to M3. **g**) Titration curve and thermogram of a representative ITC experiment measuring binding of the G12 variant S1 to doxycycline. **h**) Results of the ITC experiment measuring binding of S1 to doxycycline. **i**) Results of FTS measuring fluorescence of doxycycline when binding to S1.

Figure S10

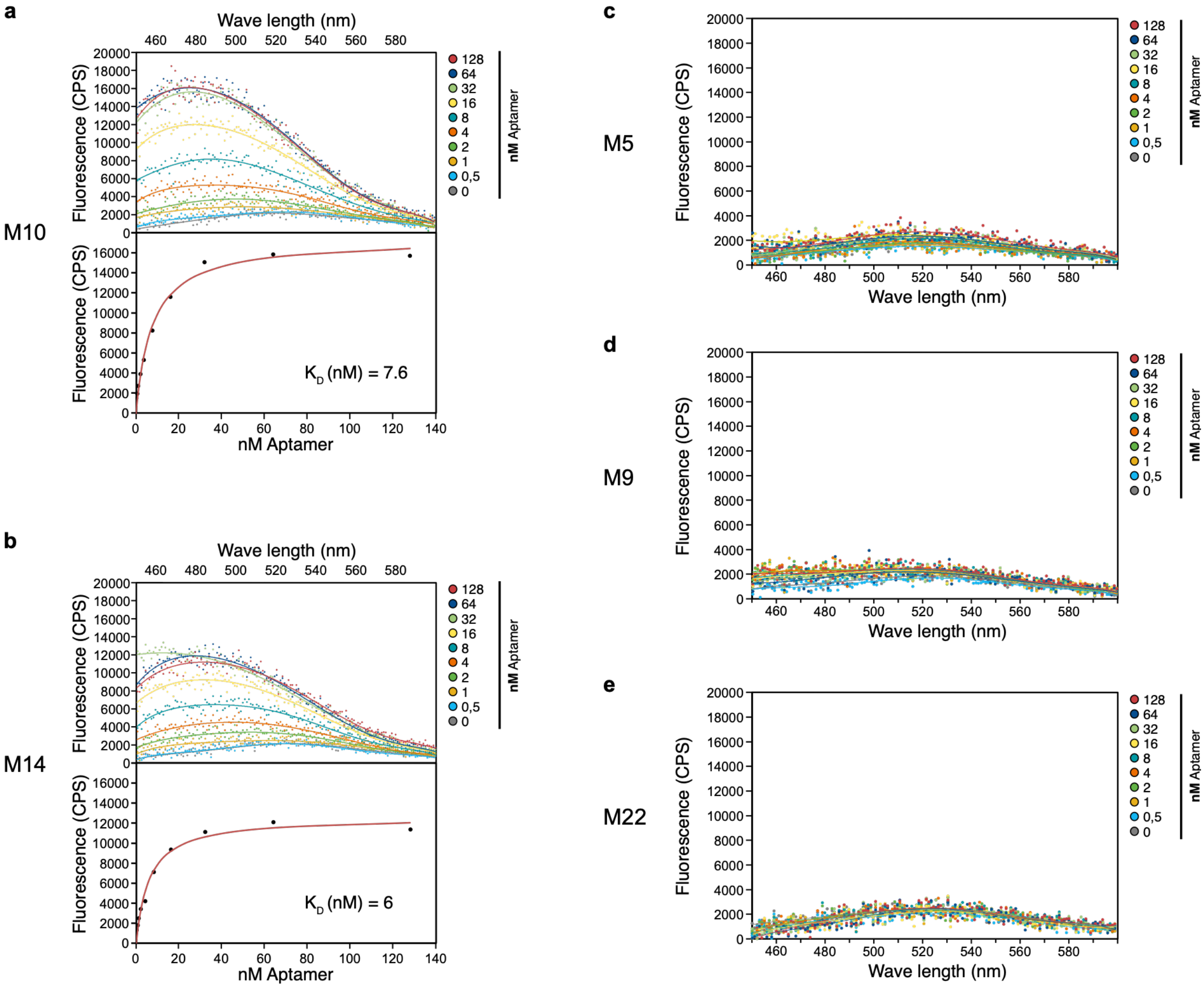

Figure S10: Fluorescence titration of rational mutations of G12. **a**) Results of FTS measuring fluorescence of doxycycline when binding to G12_M10. Shown are the measured fluorescence at different aptamer concentrations and the fluorescence levels at 490 nm wavelength used for calculation of the K_D. The calculated K_D of the respective measurement is given below the curve. Black dots depict the experimental data while the red line represents the fitted Hill equation. The calculated K_D of this mutant indicates no change in binding behaviour compared to G12. **b**) Results of FTS measuring fluorescence of doxycycline when binding to G12_M14. The calculated K_D of this mutant indicates no change in binding behaviour compared to G12. **c**) Results of FTS measuring fluorescence of doxycycline adding G12_M5. No increase in doxycycline fluorescence was detected. **d**) Results of FTS measuring fluorescence of doxycycline adding G12_M9. No increase in doxycycline fluorescence was detected. **e**) Results of FTS measuring fluorescence of doxycycline adding G12_M22. No increase in doxycycline fluorescence was detected.

Figure S11

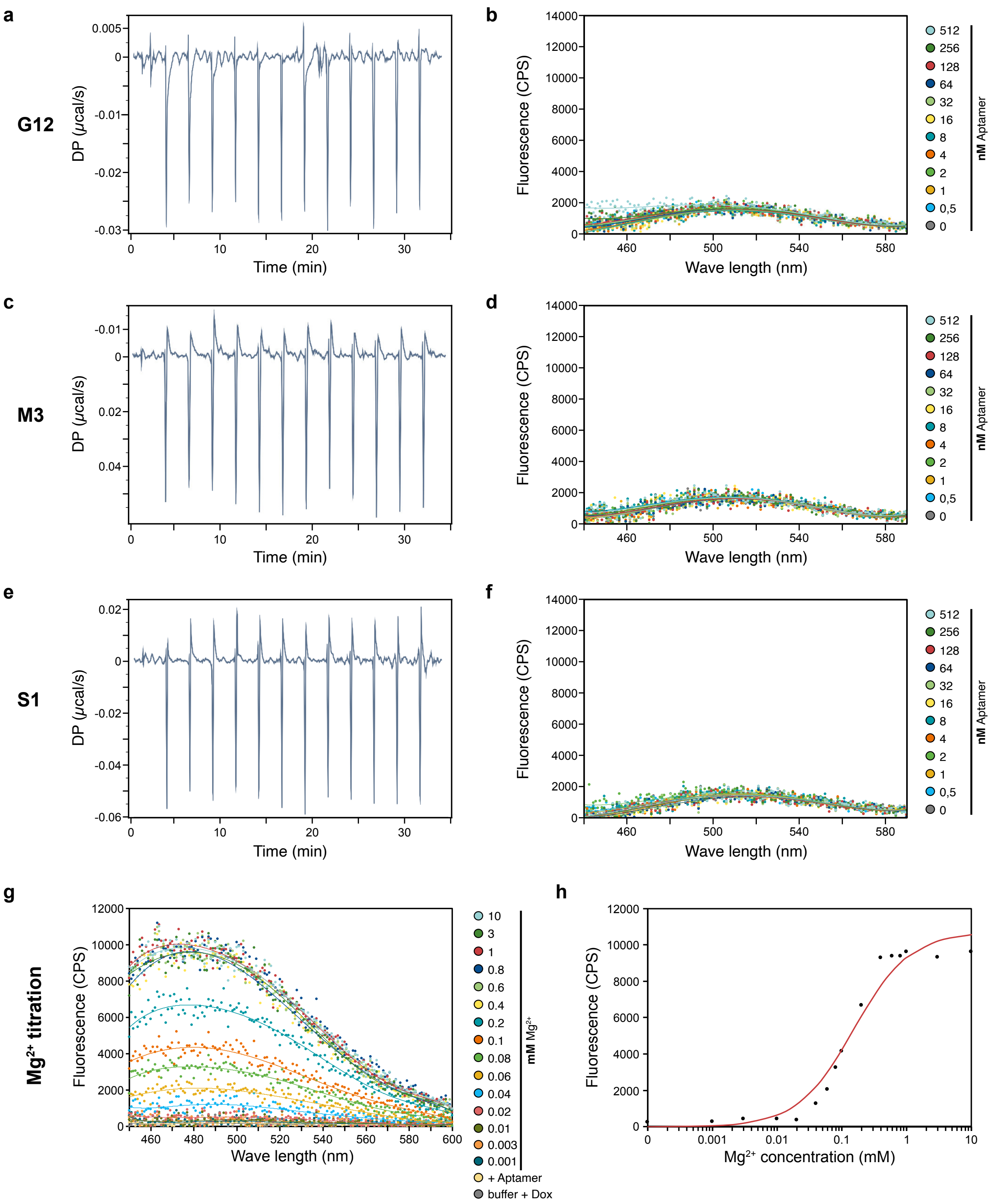

Figure S11: Testing binding of the aptamer G12 and its variants M3 and S1 to tetracycline and testing magnesium dependence of G12 for the binding to doxycycline. a) Titration curve of ITC titrating tetracycline to G12. No signs of binding were observed. **b**) Fluorescence signal when adding G12 to tetracycline in an FTS experiment. No increase of fluorescence was observed up to an aptamer concentration of 512 nM. **c**) Titration curve of ITC titrating tetracycline to M3. No signs of binding were observed. **d**) Fluorescence signal when adding M3 to tetracycline in an FTS experiment. No increase of fluorescence was observed up to an aptamer concentration of 512 nM. **e)** Titration curve of ITC titrating tetracycline to S1. No signs of binding were observed. **f**) Fluorescence signal when adding S1 to tetracycline in an FTS experiment. No increase of fluorescence was observed up to an aptamer

concentration of 512 nM. **g**) Test of magnesium-dependency of G12 for binding to doxycycline. The fluorescence on the y-axis was observed for a range of magnesium concentrations from 1 μM to 10 mM. First signs of fluorescence appeared with a magnesium concentration of 40 μM. **h**) Based on the experimental data of the FTS experiment with varying magnesium concentrations, a Hill curve (red) was modelled based on the measured fluorescence (black dots) reassembling binding of doxycycline to G12 at varying magnesium concentrations. Calculation of the binding revealed a K_D of 210 μM.

Figure S12

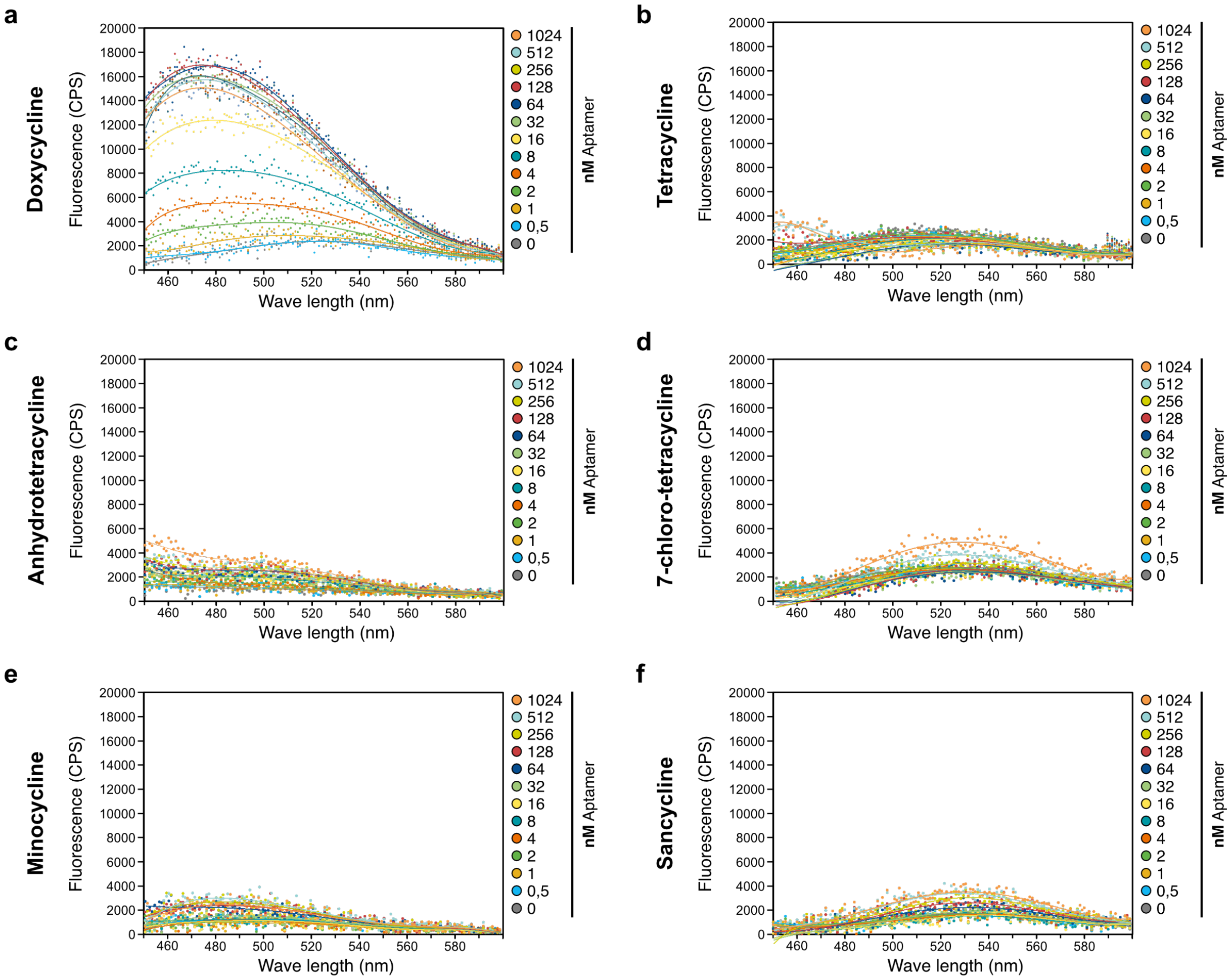

Figure S12: Fluorescence titration spectroscopy of G12 with doxycycline derivatives. a) Fluorescence signal when adding G12 to doxycycline in an FTS experiment. First signs of increased fluorescence were detected at the addition of 1 nM G12 and no further increase in fluorescence was observed from 32 nM and higher. **b**) Fluorescence signal when adding G12 to tetracycline in an FTS experiment. No signs of increased fluorescence were detected up to an aptamer concentration of 1024 nM. **c**) Fluorescence signal when adding G12 to anhydrotetracycline in an FTS experiment. No signs of increased fluorescence were detected up to an aptamer concentration of 1024 nM. **d**) Fluorescence signal when adding G12 to 7-chloro-tetracycline in an FTS experiment. A minimal increase in fluorescence was observed upon addition of 1024 nM aptamer. **e**) Fluorescence signal when adding G12 to minocycline in an FTS experiment. No signs of increased fluorescence were detected up to an aptamer concentration of 1024 nM. **f**) Fluorescence signal when adding G12 to sancycline in an FTS experiment. No signs of increased fluorescence were detected up to an aptamer concentration of 1024 nM.

Figure S13

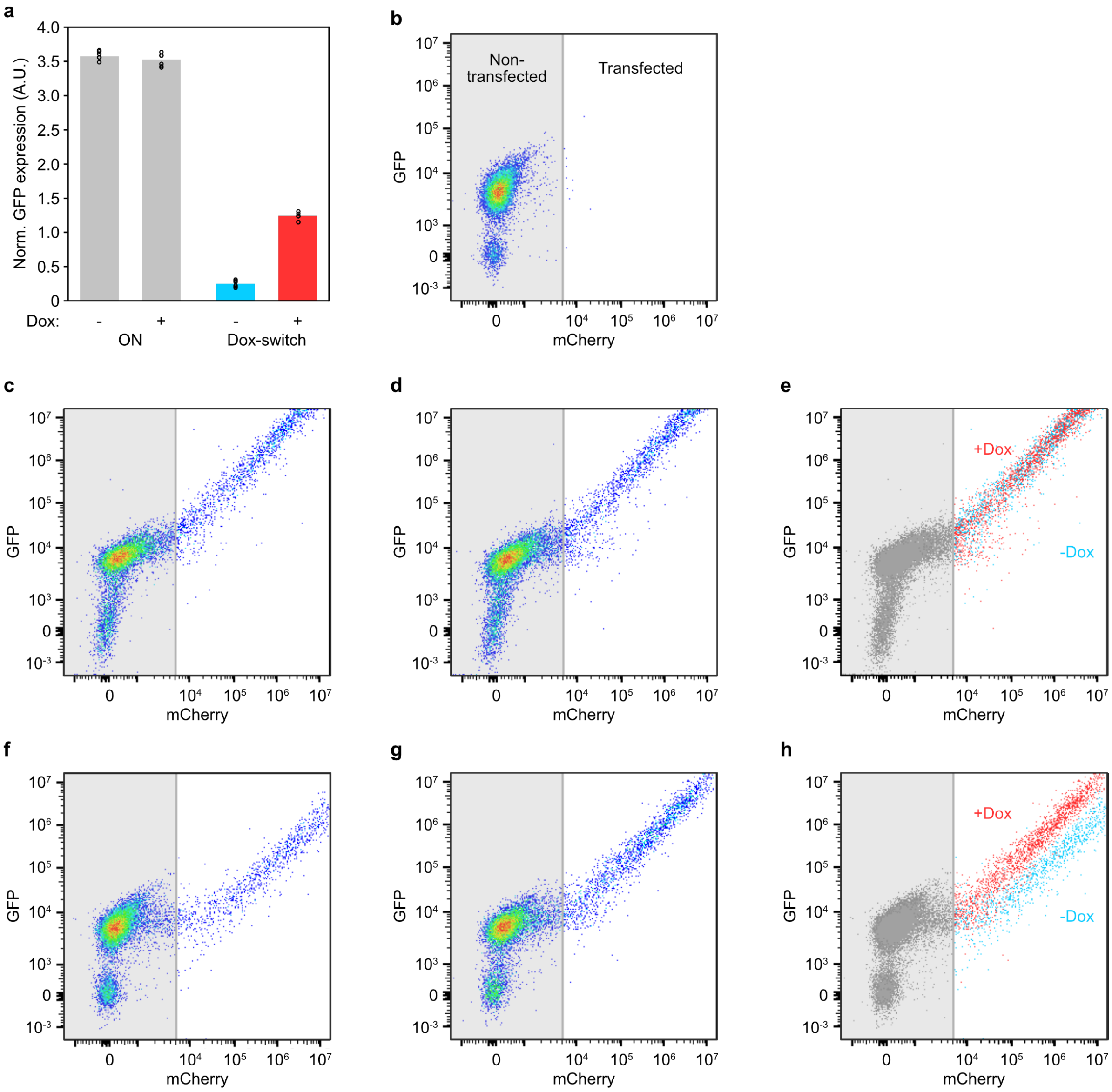

Figure S13: Test of the G12-based splice-regulator controlling GFP expression in human cells. **a**) Bar plot of the mean fluorescence values from two independent measurements with triplicates (n = 6) depicting normalised GFP expression levels (GFP/mCherry) of the constitutive GFP expressing control (ON) and the G12-based splice riboswitch (Dox-switch) with or without addition of 50 μM doxycycline in human HEK293 cells. The cells were transfected with a plasmid containing GFP (with or without the splice-regulator) and a plasmid constitutively expressing mCherry for normalisation. **b**) Cytometry data of the untransfected control with indication of the set gates for distinguishing between transfected and transfected cells. **c**) Cytometry data of the constitutive GFP expressing control (ON) in the absence of doxycycline, **d**) in the presence of 50 μM doxycycline and **e**) overlay of both measurements. **f**) Cytometery data of the G12-based splice riboswitch (Dox-switch) in the absence of doxycycline, **g**) presence of 50 μM doxycycline and **h**) overlay of both measurements. Statistical data of the shown measurements are given in Table S8 (p.38).

Figure S14

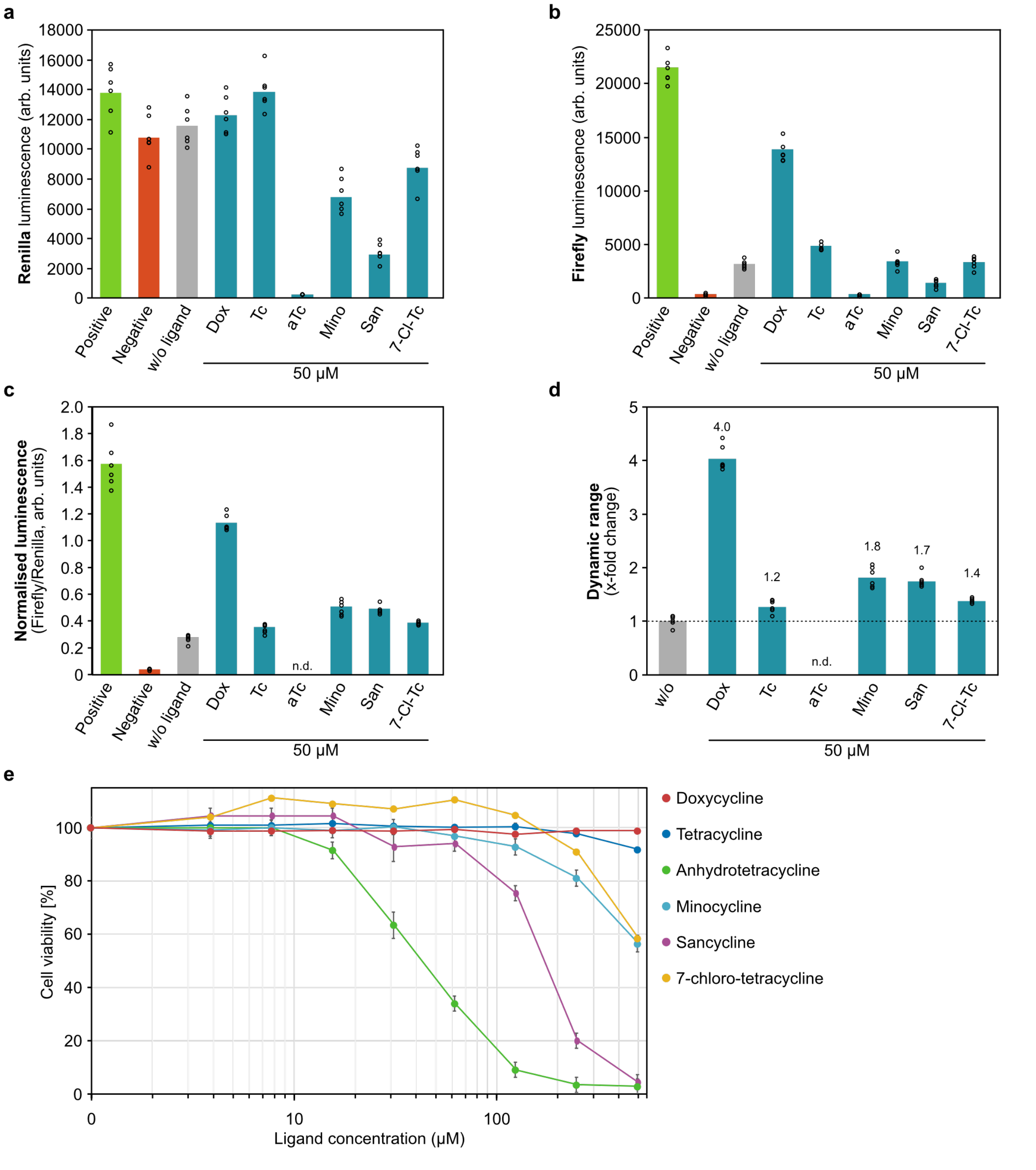

Figure S14: Test of ligand specificity of G12 in human cells. a) *Renilla* luminescence and **b**) Firefly luminescence measured after 24 h with or without ligand. Shown are positive and negative control, the splice-controlling riboswitch without addition of a ligand (w/o) and with addition of 50 μM of one of the six different ligands. The luminescence values in samples with anhydrotetracycline (aTc) were almost not detectable. Also cells with addition of minocycline (Mino), sancycline (San) and 7-chloro-tetracycline (7-Cl-Tc) showed lower Firefly and *Renilla* luciferase luminescence, indicating unspecific effects. (n = 6) **c**) Normalised luminescence values (Firefly / *Renilla* luminescence). Note that it was impossible to calculate reliable values for anhydrotetracycline, sue to the very low *Renilla* and Firefly luciferase luminescence. **d**) Calculated dynamic range (norm. luminescence $_{\text{w/o ligand}}$ / norm. luminescence $_{\text{w/ ligand}}$) in response to 50 μM of respective ligand. The expression level in the absence of any ligand (w/o) is indicated by a dashed line. Note that the dynamic ranges calculated for minocycline, sancycline and 7-chloro-tetracycline are not caused by an increase in Firefly luciferase luminescence but a decreased *Renilla* luciferase luminescence. Doxycycline (Dox) shows the expected 4-fold increase in Firefly

luciferase luminescence whereas addition of tetracycline (Tc) has no or minimal impact. Shown are the mean and standard deviation of two independent measurements performed in triplicates. **e**) Cell viability assay with the six used ligands using alamarBlue. Eight different concentrations of each ligand were tested for their impact on human HeLa cell viability over 24 h. The high toxicity effect of anhydrotetracycline explains the low luminescence values. Sancycline, minocycline and 7-chlorotetracycline also show toxic effects on human cells in higher concentrations (> 50 μM). Shown are mean values and standard deviation of one measurement performed in triplicates (n = 3).

Figure S15

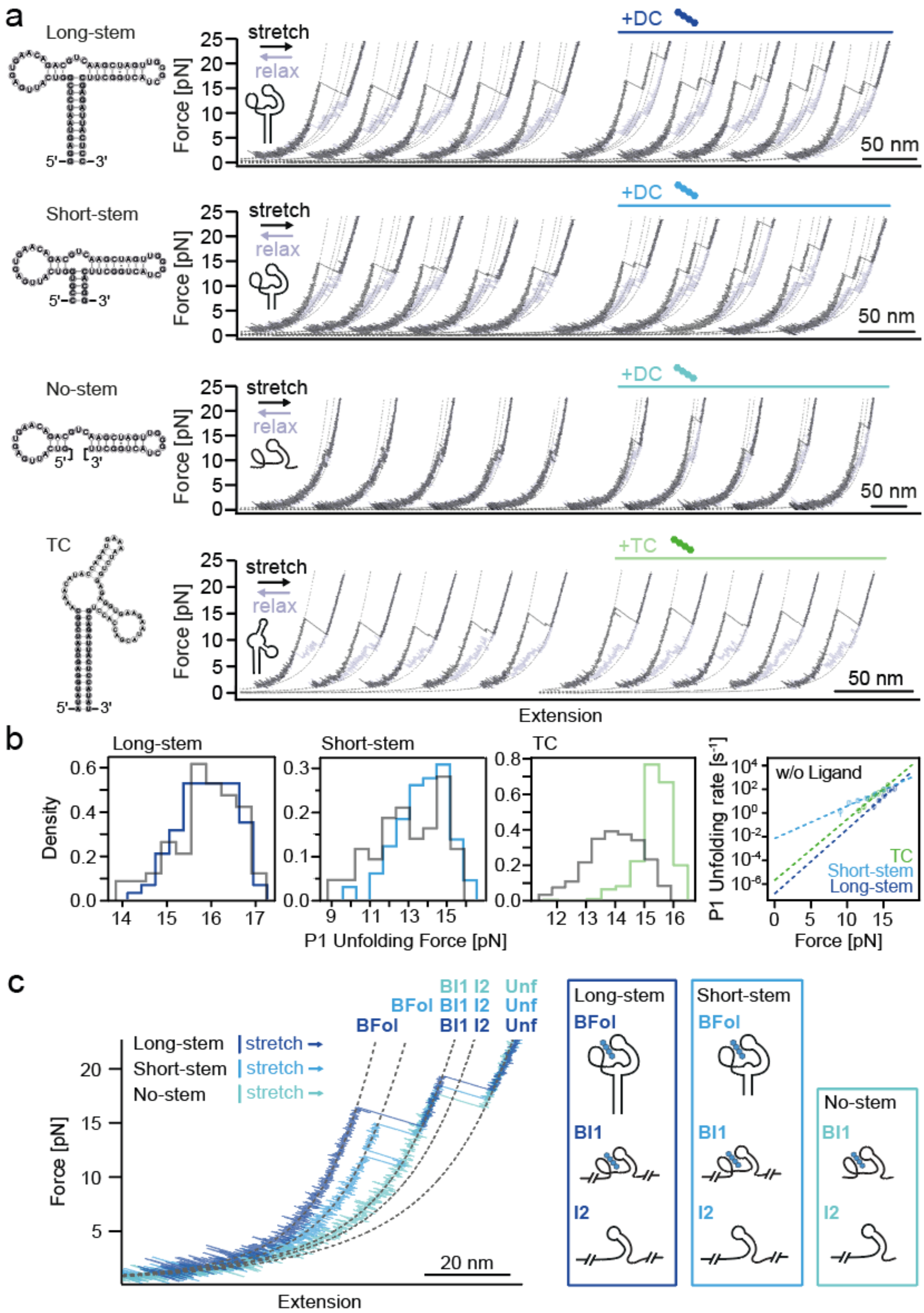

a
Long-stem
Short-stem
No-stem
TC
stretch
relax
+DC
+TC
Force [pN]
50 nm
Extension
b
Long-stem
Short-stem
TC
Density
P1 Unfolding Force [pN]
P1 Unfolding rate [s-1]
w/o Ligand
Force [pN]
c
BFol
BI1
I2
Unf
stretch
20 nm

Figure S15: Comparison of G12 variants and the tetracycline aptamer in force-ramp experiments. **a**) Representative force-ramp traces for the "long-stem," "short-stem," and "no-stem" doxycycline G12 aptamer constructs, as well as the tetracycline (TC) aptamer containing a 16-bp stem. For each construct, five traces recorded without ligand (left) and five traces recorded with ligand (right) are shown. Predicted secondary structures with sequence information are displayed on the left. **b**) Histograms of P1 unfolding forces for the long-stem, short-stem, and TC constructs in the presence and absence of ligand. In contrast to the doxycycline constructs, the P1 stem of the TC aptamer unfolds at higher forces upon ligand binding. Long-stem (bound) n = 90, (unbound) n = 167; Short-stem (bound) n = 93, (unbound) n = 60; TC (bound) n = 444, (unbound) n = 265. On the right, unfolding rates of the P1 stem in the absence of ligand are plotted as a function of force and extrapolated using a Bell model. Fit parameters are provided in Table S11. The P1 stem of the short-stem construct is kinetically more labile than those of the long-stem construct and the TC aptamer, as indicated by its higher zero-force unfolding rate. Data are presented as mean ± s.e.m. **c**) Overlay of representative force-ramp traces for the three G12 constructs in the presence of doxycycline. All three variants stabilise the same intermediate I1 (BI1), independent of the P1 stem.

Figure S16

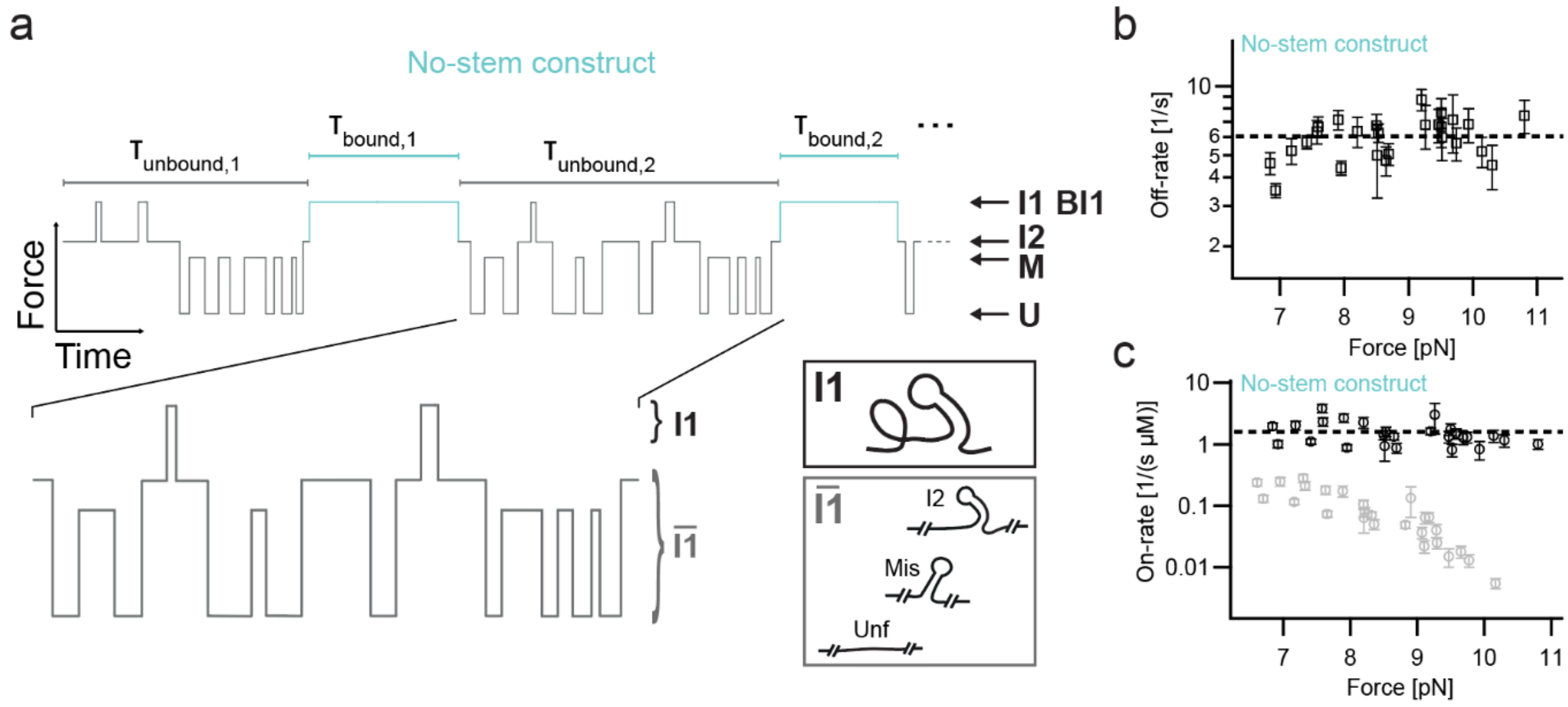

Figure S16: Determination of doxycycline ON and OFF rates measured by SMFS. a) Schematic passive-mode trace of the G12 no-stem construct, with force levels marking different intermediates on the right. Doxycycline binding stabilizes the I1 state (bound I1 = BI1), resulting in longer I1 lifetimes and reduced I1→I2 unfolding rates. Bound intervals are shown in light blue and unbound intervals in grey. Off-rates are calculated as the inverse of the mean bound-state lifetime. A straightforward estimate of the on-rate (apparent on-rate) would use the inverse of the mean unbound-state lifetime divided by the doxycycline concentration. However, because unbound intervals contain several force-dependent states, and doxycycline may bind differently to U versus I1, this apparent on-rate becomes strongly force-dependent. To correct for this, an effective on-rate is derived by weighting by the relative population probability of I1. **b**) Off-rates for doxycycline dissociation from the no-stem aptamer. No significant force dependence is observed, and the data are well described by a constant rate (n = 26). **c**) On-rates for doxycycline binding to the no-stem construct. On-rates obtained with the non-effective approach are shown in grey (n = 26), and those using the effective method in black (n = 26). The non-effective on-rates decrease strongly with force, implying faster binding to I1 than to other states. In contrast, the effective on-rate is nearly force-independent, consistent with doxycycline binding exclusively to the I1 conformation via a conformational-capture mechanism. Data points in b) and c) are presented as mean ± s.e.m.

Figure S17

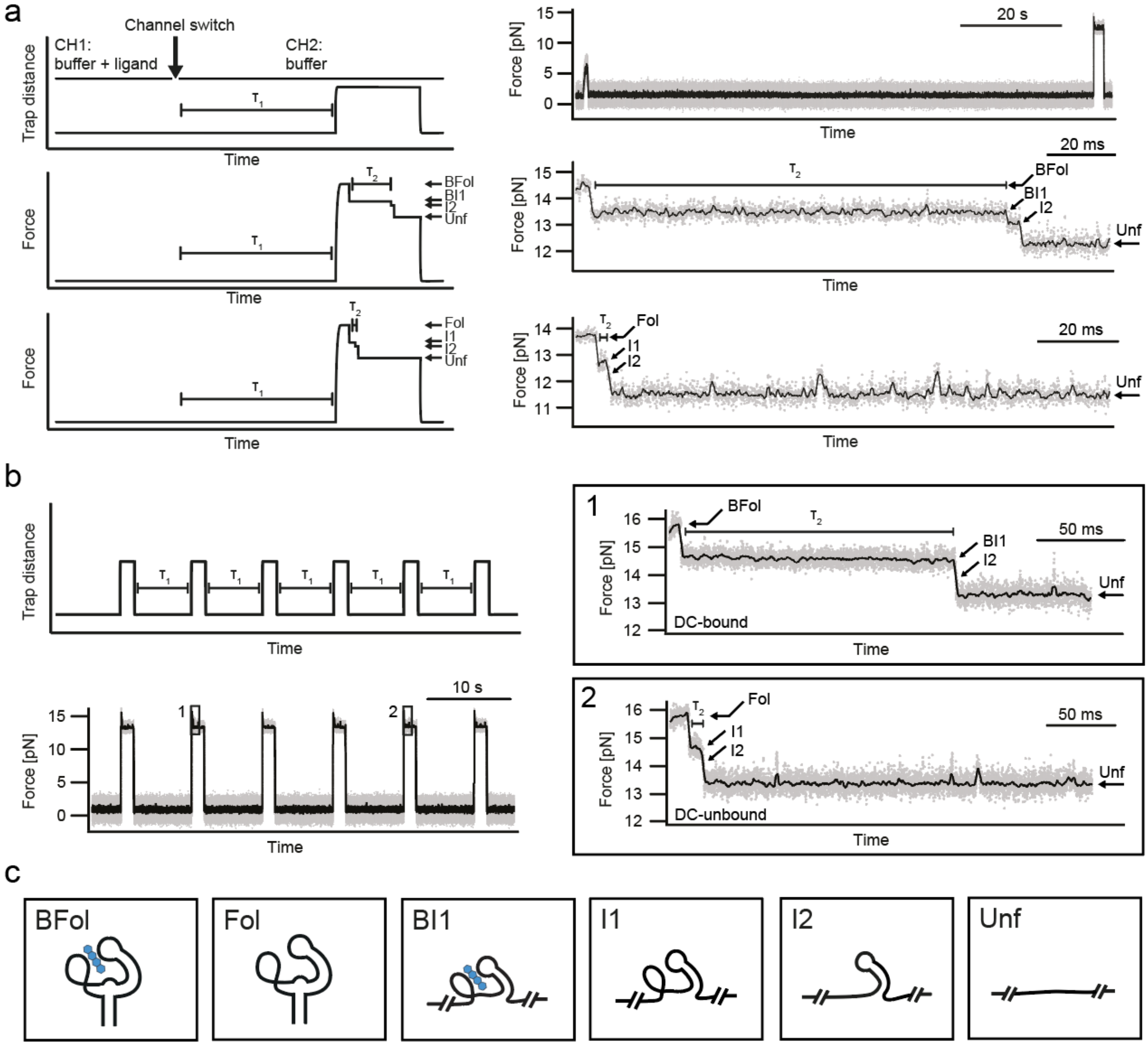

Figure S17: Determination of doxycycline ON and OFF rates for the short-stem G12 construct using single-molecule force-jump experiments. a) OFF-rate measurement. Left: Schematic of the force-jump protocol. The aptamer is first allowed to bind doxycycline in microfluidic channel 1 (CH1) at high ligand concentration. The doxycycline-bound aptamer is then transferred to channel 2 (CH2), which contains no ligand. After a defined waiting time (τ_1), a force jump is applied. From the unfolding response, specifically, the dwell time in I1, it can be determined whether doxycycline remained bound until the jump. Repeating this measurement for different τ_1 values and calculating the fraction of bound versus unbound molecules allows determining the average off-rate (see Materials and Methods). Right: Representative traces of doxycycline-bound (DC-bound) and unbound (DC-unbound) force-jump unfolding fingerprints. **b**) ON-rate measurement. Upper left: Schematic of the force-jump strategy for determining on-rates. Successive force jumps with a fixed waiting time allow quantification of the fraction of aptamers that rebind doxycycline. A representative trace is shown (lower left), along with zoom-ins (upper and lower right). As in the off-rate experiment, the dwell time in I1 indicates whether binding occurred during the waiting interval τ_1. Notably, after any prior unfolding and unbinding event, the aptamer always begins the new waiting period in the unbound state. Performing this protocol across different doxycycline concentrations and waiting times enables extraction of on-rates to the folded aptamer construct (see Materials and Methods). **c**) Schematics of the intermediates populated during the OFF-rate (a) and ON-rate (b) measurements.

Figure S18

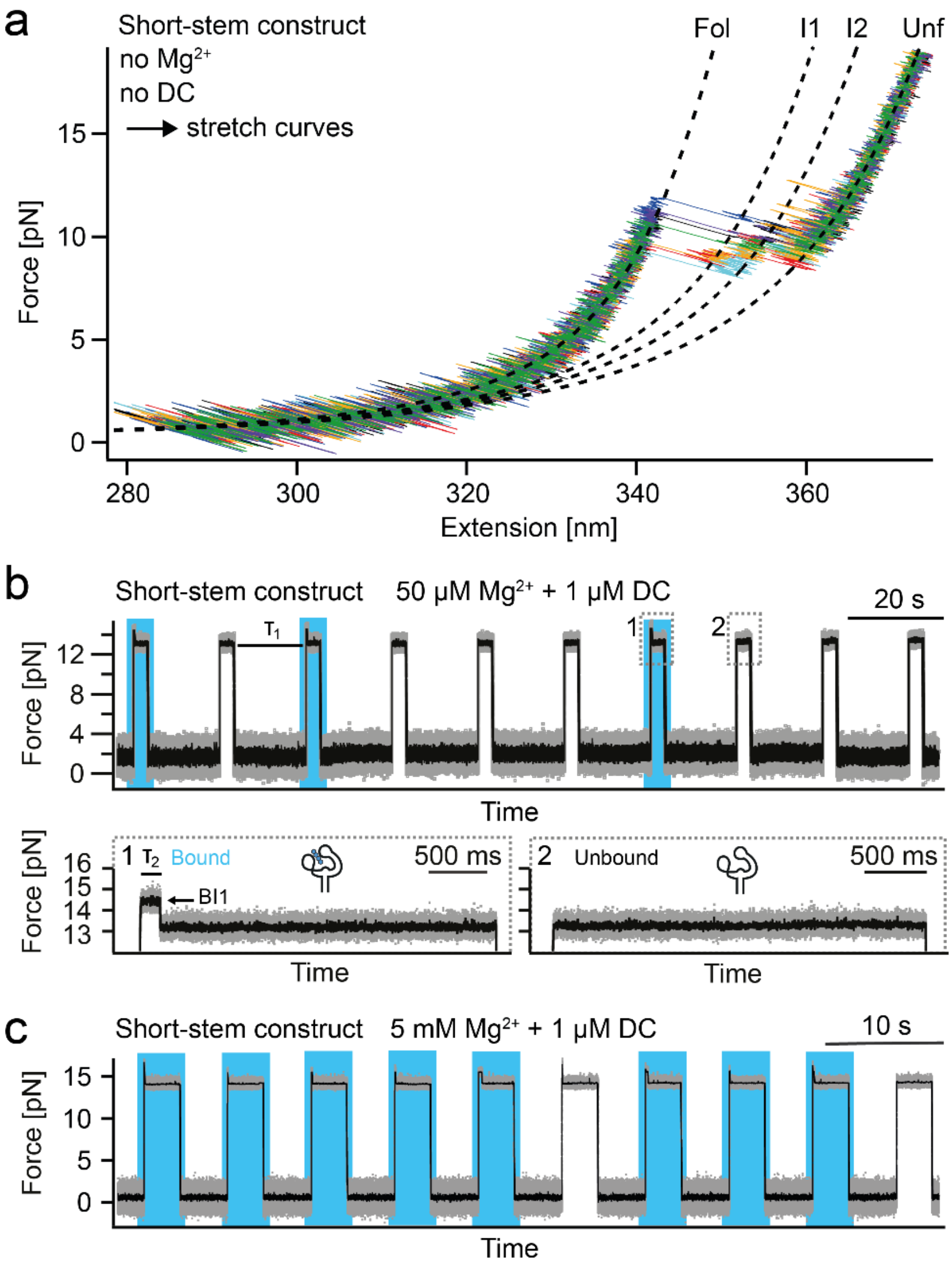

Figure S18: Effect of Mg^{2+} on aptamer folding and doxycycline binding. a) Representative force-ramp traces of the G12 short-stem construct in the absence of magnesium and doxycycline. All on-pathway intermediates (I1, I2) are still observed, indicating that the folding pathway remains largely unchanged. Slightly lower unfolding forces likely result from the reduced ionic strength without magnesium. **b**) Force-jump measurements at 50 μM magnesium and 1 μM doxycycline (DC). Representative force-jump traces show the fraction of aptamers bound or unbound after a waiting time of 15 s. Zoom-ins (1 and 2) illustrate unfolding patterns where DC was bound (1) or unbound (2), determined from I1 dwell times, analogous to the on- and off-rate analysis in Fig. S9. Doxycycline binding is limited under these low magnesium conditions. **c**) Representative force-jump traces at 5 mM Mg^{2+} and 1 μM DC. Doxycycline binding is recovered at this higher magnesium concentration. Instances where DC did not bind can be entirely attributed to insufficient equilibration time at low force.

Figure S19

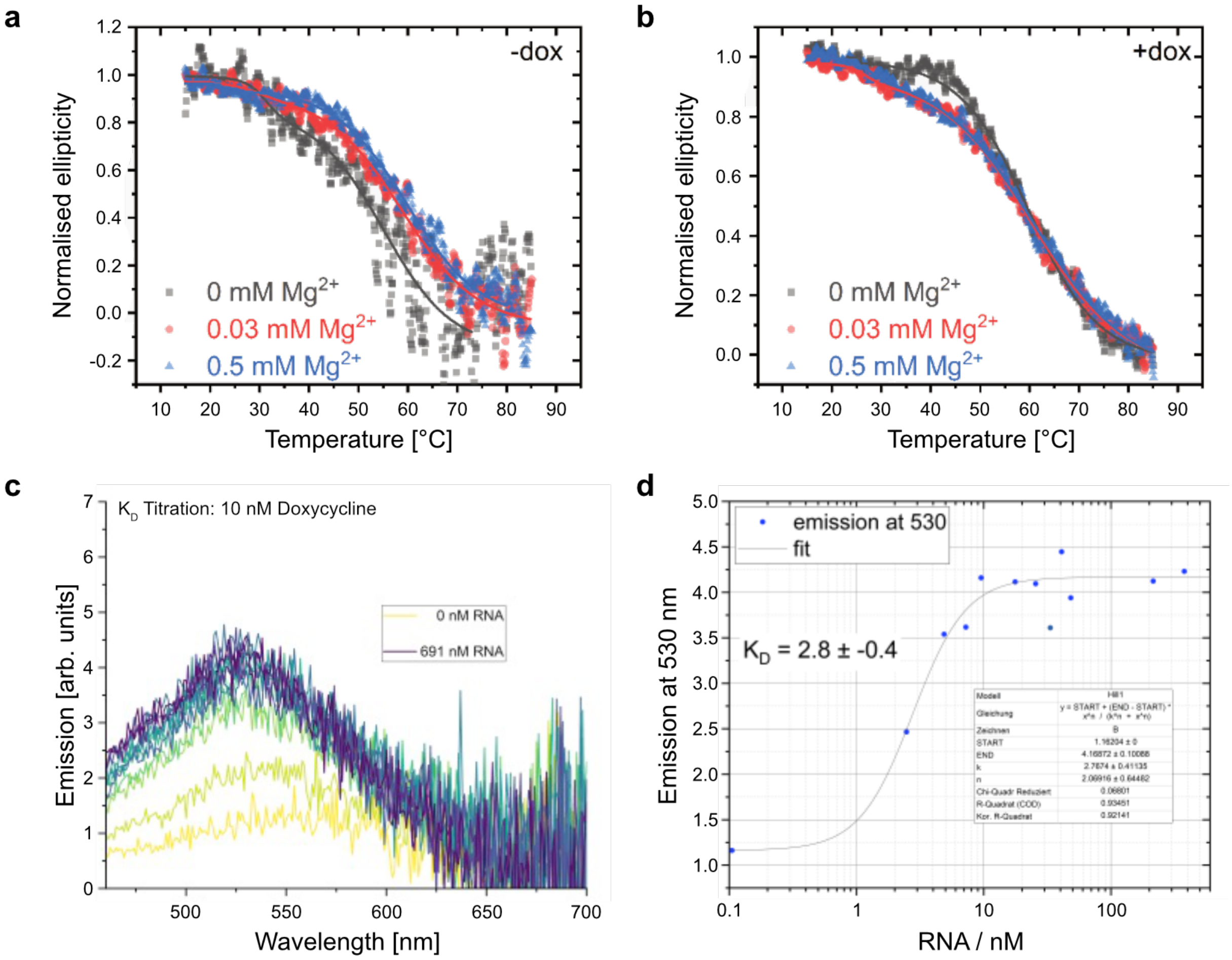

Figure S19: Circular dichroism thermal melting assay and initial fluorescence titration of G12
All spectra were measured using an FP-8500 fluorescence spectrometer (Jasco) in a 2x10 mm quartz cuvette, with background correction, solvent excitation correction, and reabsorption correction applied. **a**) Circular dichroism thermal melting assay of G12 in presence of 0/0.03/0.5 mM (black/red/blue) magnesium without doxycycline and **b**) with doxycycline. The dots represent the experimental data, while the lines show the obtained fit according to the model (see Table S14). **c**) A concentration of 10 nM doxycycline was used, and spectra were acquired at varying amounts of added G12 until saturation was reached at a constant temperature of 20 °C, with an excitation wavelength of 375 nm. The emission at 530 nm was utilized to determine the K_D value using the Hill equation. A K_D value of 2.8 nM was obtained. The corrected fluorescence spectra of doxycycline (yellow) and increasing amounts of G12 (color gradient towards blue). **d**) The emission at 530 nm is plotted against the added RNA concentration. The black line represents the fit via a Hill equation and yields a K_D-value of 2.8 nM.

Supplementary Methods

Kinetic modelling analysis: The dataset was fitted with the software DynaFit4 (Kuzmič, P. (1996). “Program DYNAFIT for the analysis of enzyme kinetic data: application to HIV proteinase.” *Analytical biochemistry*, *237*(2), 260-273.). DynaFit4 translates symbolic reaction schemes into matrices and solves multiple equilibrium problems. The solutions of these problems can be stated as:

$$S(t) = S_0 + \sum_{i=0}^{n} r_i c_i(t) \quad (1)$$

Here, $S(t)$ is the measured time-dependent fluorescence signal with a fixed starting offset S_0. n is the number of distinct species involved in the reaction scheme, each characterized by a corresponding fluorescence contribution change value (response coefficient) r and the concentration c, which varies during the binding reaction. The response coefficients are derived from the fluorescence contribution of each species. As the analyzed time traces represent the difference between the fluorescence at time t and the initial fluorescence, the response coefficient signifies the magnitude of fluorescence change associated with each species.

In this study, we employed a model selection approach incorporating the root mean square error (RMSE) as well as the Akaike Information Criterion (AIC, Burnham, K. B., and Anderson, D. R. (2002). *Model Selection and Multimodel Inference: A Practical Information-Theoretic Approach*. Springer New York). The AIC penalizes models with a large number of parameters, preventing overfitting. The AIC is calculated as:

$$\mathrm{AIC} = \left[-\log\left(\mathrm{L_p}\right) + 2\ \mathrm{p}\right] \quad (2)$$

where p is the number of parameters in the model and *L* is the maximum likelihood. A lower AIC value indicates a better model fit. We use the ΔAIC-value, where the lowest AIC value is subtracted from each AIC value. Thus 0 is the smallest AIC value and the most likely model. The considered models (Table 1) represent various conformational states of the protein: R_1 and R_2 (ligand-free G12 conformations), R_1^* and R_2^* (ligand-associated intermediate G12 conformations), and B (ligand-bound complex).

A single binding step model proved Insufficient to accurately represent the experimental data. Therefore, we explored two-state models with starting conformations R_1 and R_2. Subsequently, an intermediate conformation was introduced to account for ligand contact.

Several binding scenarios were considered: (1) only one conformation is binding competent, binding occurs via an intermediate state, (2) both conformations are binding competent, binding occurs via the same intermediate state, and (3) both conformations are binding competent, binding occurs via separate intermediate states. Additionally, various back reactions were investigated to refine the model.

It is Important to note that models with multiple Initial conformations can be sensitive to the initial concentration of each component. To assess the impact of initial concentrations, we explored different ratios of R_1 and R_2: 1:0, 0:1, and 1:1.

The calculated rate constants for each model are provided in the attached ZIP folder as an HTML file. The AIC and RMSE values, as determined by DynaFit4, are listed in Table S9. The models are ranked according to their ΔAIC values, with the lowest ΔAIC indicating the best fit. For each model and each individual SF time trace, we received the percentage of each population for each timepoint. In Figure 4h we show the population variation over the measuring window of all eq. of the best fitting model to illustrate the ligand binding process.

Supplementary Tables

Table S1: Selection parameters and elution fraction of the column-SELEX

**Selection round**	Washing steps	Counter-selection w/ kanamycin A (*μ*M) *	Concentration doxycycline for elution (*μ*M)	Eluted RNA (% relative to input)
**S1**	12	0	500	-
**S2**	12	0	500	0.01
**S3**	12	0	500	0.01
**S4**	12	0	500	0.22
**S5**	12	0	500	5.61
**S6**	18	0	500	3.98
**S7**	18	500	500	0.09
**S8**	18	500	500	1.42
**S9**	18	500	500	11.28
**S10**	18	0	50	35.03

* Counter-selection with kanamycin was introduced from round S7 to S9 after observing inefficient removal of weak bound RNAs even after 18 washing steps in round S6.

Table S2: Selection parameters and eluted fraction of the Capture-SELEX.

Selection round	Washing steps	Eluted background (% relative to input)	Concentration doxycycline for elution (μM)	Eluted RNA (% relative to input)
**C1**	3	-	100	-
**C2**	3	3.07	100	2.38
**C3**	3	2.89	100	2.54
**C4**	3	3.66	100	3.05
**C5**	3	2.83	100	3.78
**C6**	3	5.35	100	18.65
**C7**	3	6.63	100	37.24

Table S3: Cytometer data from the cell-sorting prior to *in vivo* screening in yeast.

			Events	% Total	% Parental
Initial pool		All Events	56755	100%	100%
		Yeast	52654	92.77%	92.77%
		Singlet	50877	89.64%	96.63%
		mCherry	46047	81.13%	90.51%
		GFP	4561	8.04%	9.91%
Capture-SELEX	Round C5	All Events	100000	100%	100%
		Yeast	94762	94.76%	94.76%
		Singlet	88053	88.05%	92.92%
		mCherry	83374	83.37%	94.69%
		GFP	57701	57.70%	69.21%
	Round C7	All Events	100000	100%	100%
		Yeast	95812	95.81%	95.81%
		Singlet	91742	91.74%	95.75%
		mCherry	86486	86.49%	94.27%
		GFP	58280	58.28%	67.39%
Column-SELEX	Round S6	All Events	10000	100%	100%
		Yeast	8996	89.96%	89.96%
		Singlet	8793	87.93%	97.74%
		mCherry	8125	81.25%	92.40%
		GFP	3748	37.48%	46.13%
	Round S8	All Events	100000	100%	100%
		Yeast	95146	95.15%	95.15%
		Singlet	88611	88.61%	93.13%
		mCherry	83506	83.51%	94.24%
		GFP	19485	19.49%	23.33%
	Round S10	All Events	10000	100%	100%
		Yeast	4601	46.01%	46.01%
		Singlet	4187	41.87%	91.00%
		mCherry	3830	38.30%	91.47%
		GFP	510	5.1%	13.32%

Table S4: Regulatory performance of G12 mutants tested in yeast.

Aptamer	Expression w/o doxycycline (%)	Expression w/ 100 μM doxycycline (%)	Dynamic range (x-fold change)
none (pCBB05)	100	97.3	1.0
G12	69.8	6.4	11.0
M1	44.4	3.2	13.8
M2	21.2	2.3	9.2
M3	65.7	4.2	15.6
M4	92.6	90.6	1.0
M5	66.8	66.6	1.0
M6	66.4	9.7	6.8
M7	61.3	58.5	1.0
M8	49.3	46.8	1.1
M9	75.0	68.4	1.1
M10	47.7	4.9	9.7
M11	52.8	3.6	14.5
M12	52.6	14.8	3.6
M13	57.4	48.0	1.2
M14	76.0	6.0	12.7
M15	73.5	37.3	2.0
M16	82.9	68.9	1.2
M17	66.4	5.6	11.8
M18	84.1	75.4	1.1
M19	54.5	41.6	1.3
M20	75.3	70.2	1.1
M21	14.8	13.2	1.1
M22	76.9	70.8	1.1
M23	37.1	33.4	1.1
S1	40.1	2.0	19.9
S2	63.1	3.4	18.8
S3	33.8	2.1	15.9
S4	33.6	2.8	12.0
S5	69.4	5.9	11.7

Table S5: Cytometer data of the randomised mutation screening in yeast.

			Events	% Total	% Parent
w/o Dox	Sort 1	All Events	100000	100%	100%
		Yeast	95355	95.36%	95.36%
		Singlet	92030	92.03%	92.03%
		P5	15002	15.00%	16.30%
		P6	10686	10.69%	71.23%
		P7	10656	10.66%	99.72%
	Sort 2	All Events	100000	100%	100%
		Yeast	94864	94.86%	94.86
		Singlet	92090	92.09%	97.08%
		P5	14924	14.92%	16.21%
		P6	10227	10.23%	68.53%
		P7	10318	10.32%	100.01%
w/ Dox	Sort 1	All Events	100000	100%	100%
		Yeast	96800	96.80%	96.80%
		Singlet	73417	73.42%	75.84%
		P5	16104	16.10%	21.93%
		P6	13057	13.06%	81.08%
		P7	602	0.60%	0.05%
	Sort 2	All Events	100000	100%	100%
		Yeast	94171	94.17%	94.17%
		Singlet	87912	87.91%	93.35%
		P5	19469	19.47%	22.15%
		P6	14362	14.36%	73.77%
		P7	1227	1.23%	8.54%

Table S6: Dose dependency of the regulatory activity of G12 and its variants in yeast.

	G12		M3		S1	
µM doxycycline	Expression (%)	Dynamic range	Expression (%)	Dynamic range	Expression (%)	Dynamic range
0	67.7	1.0	64.6	1.0	40.9	1.0
0.01	69.1	1.0	62.9	1.0	39.2	1.0
0.1	63.5	1.1	59.6	1.1	38.0	1.1
0.5	55.8	1.2	53.4	1.2	33.3	1.3
1	48.8	1.4	43.9	1.5	29.0	1.4
5	27.4	2.5	25.0	2.6	14.7	2.6
10	19.0	3.6	16.1	4.0	9.5	4.3
25	11.7	5.8	9.4	6.8	5.3	7.7
100	6.2	10.9	4.1	15.9	2.7	15.5
250	4.2	16.0	2.7	23.8	2.1	23.7
**EC_{50}**	**2.7 ± 0.7 µM**		**2.6 ± 0.6 µM**		**2.6 ± 0.4 µM**	

Table S7: Regulatory potential of different synthetic riboswitches in yeast.

Aptamer	Expression w/o ligand (%)	Expression w/ 100 μM ligand (%)	Expression w/ 250 μM ligand (%)	Fold-change 100 μM ligand	Fold-change 250 μM ligand
Tetracycline cb32[1]	30.7	8.9	5.5	3.4	5.6
Tetracycline G6[2]	31.2	5.1	4.0	6.1	7.7
Tobramycin T1[3]	38.8	4.4	4.0	8.8	9.6
Tobramycin N1-G6[3]	36.2	4.4	3.9	8.2	9.3
Neomycin M4[4]	27.6	4.8	3.9	5.7	7.2
Doxycycline G12	64.3	6.3	4.6	10.3	14.1
Doxycycline M3	61.3	4.4	3.1	14.1	20.0
Doxycycline S1	38.2	2.1	1.5	18.0	25.0

[1] Suess, B., Hanson, S., Berens, C., Fink, B., Schroeder, R., & Hillen, W. (2003). Conditional gene expression by controlling translation with tetracycline-binding aptamers. *Nucleic acids research*, *31*(7), 1853-1858.

[2] Groher, A. C., Jager, S., Schneider, C., Groher, F., Hamacher, K., & Suess, B. (2018). Tuning the performance of synthetic riboswitches using machine learning. *ACS synthetic biology*, *8*(1), 34-44.

[3] Kraus, L., Duchardt-Ferner, E., Bräuchle, E., Fürbacher, S., Kelvin, D., Marx, H., ... & Suess, B. (2023). Development of a novel tobramycin dependent riboswitch. *Nucleic acids research*, *51*(20), 11375-11385.

[4] Weigand, J. E., Sanchez, M., Gunnesch, E. B., Zeiher, S., Schroeder, R., & Suess, B. (2008). Screening for engineered neomycin riboswitches that control translation initiation. *Rna*, *14*(1), 89-97.

Table S8: Cytometer data of GFP/mCherry fluorescence measurements in HEK293 cells.

Construct	Doxycycline	Population	% Parent	Events	Mean GFP	Mean mCherry
None	-	All Events	100.00%	11642	5157	216
		Cells	85.90%	10000	5601	215
		Singlets	80.95%	8095	4774	180
		Transfected	0.95%	77	16844	1575
ON	-	All Events	100%	10334	1271576	454883
		Cells	75.27%	7778	1617271	579647
		Singlets	89.95%	6996	1553402	560197
		Transfected	33.03%	2311	3199278	879561
	50 μM	All Events	100%	13026	1028676	350530
		Cells	76.77%	10000	1302322	446274
		Singlets	90.14%	9014	1224933	425528
		Transfected	29.47%	2656	2936123	815658
Dox-switch	-	All Events	100%	12860	39787	183648
		Cells	77.76%	10000	49521	227223
		Singlets	84.12%	8412	47142	213217
		Transfected	13.98%	1176	161815	761853
	50 μM	All Events	100%	11208	287446	238034
		Cells	74.94%	8399	366124	302410
		Singlets	89.02%	7477	341176	279336
		Transfected	28.09%	2100	926769	738395

Table S9: Contour-length differences between intermediate states of the long-stem, short-stem, and no-stem G12 aptamer constructs as determined by SMFS.

Construct	Transition	Contour length (s.e.m.) [nm]
Long-stem	Fol - I1	23.91 (0.14)
	BFol - BI1	23.68 (0.07)
	I1 - I2	6.6 (0.3)
	BI1 - I2	6.75 (0.21)
	I2 - Unf	10.66 (0.18)
Short-stem	Fol - I1	14.6 (0.7)
	BFol - BI1	14.52 (0.13)
	I1 - I2	6.5 (0.3)
	BI1 - I2	6.3 (0.3)
	I2 - Unf	10.82 (0.17)
No-stem	I1 - I2	6.2 (0.4)
	BI1 - I2	6.8 (0.4)
	I2 - Unf	10.8 (0.3)
	Mis - Unf	6.44 (0.13)

Using a conversion factor of 0.6 nm per nucleotide[1] and accounting for fraying[2] as well as the diameter of the RNA duplex,[3] the most plausible structures associated with the Unf-I2 transition correspond to formation of the P3 stem, while the I2-I1 transition reflects formation of the P2 stem together with tertiary contacts. For all measurements the standard error of the mean (s.e.m.) is indicated behind the values.

[1] Walbrun, A., Wang, T., Matthies, M., Šulc, P., Simmel, F. C., & Rief, M. (2024). Single-molecule force spectroscopy of toehold-mediated strand displacement. *Nature communications*, *15*(1), 7564.
[2] Woodside, M. T., Behnke-Parks, W. M., Larizadeh, K., Travers, K., Herschlag, D., & Block, S. M. (2006). Nanomechanical measurements of the sequence-dependent folding landscapes of single nucleic acid hairpins. *Proceedings of the National Academy of Sciences*, *103*(16), 6190-6195.
[3] Greenleaf, W. J., Frieda, K. L., Foster, D. A., Woodside, M. T., & Block, S. M. (2008). Direct observation of hierarchical folding in single riboswitch aptamers. *Science*, *319*(5863), 630-633.

Table S10: Affinities and rate constants for doxycycline binding to the G12 aptamer constructs, measured by SMFS.

Construct	On-rates (s.e.m.) [1/(s μM)]	Off-rates (s.e.m.) [1/s]	K_D (s.e.m.) [μM]
No-stem G12	1.59 (0.15)	6.04 (0.24)	3.8 (0.4)
Short-stem G12	0.42 (0.03)	0.0055 (0.0003)	0.0131 (0.0013)

For all measurements the standard error of the mean (s.e.m.) is indicated behind the values.

Table S11: Unfolding rate analysis via single-molecule force-ramp measurements.

Construct	$-\log_{10}(k_0)$ (s.d.) [1/s]	Δx (s.d.) [nm]
Long-stem G12	6.8 (6.1, 6.9)	5.2 (0.5)
Short-stem G12	2.2 (2.1, 2.4))	2.6 (0.3)
Tetracycline aptamer	5.7 (5.4, 5.9)	4.9 (0.3)

To extract unfolding rates, we applied a model that divides force-ramp traces into short segments of nearly constant force which, under the assumption of Markovian dynamics, can be combined across multiple cycles into a virtual quasi-equilibrium dataset.[1] The resulting force-dependent rates were fitted using a Bell model. Fit parameters k_0 (unfolding rate at zero-force condition) and Δx (distance to the transition state) are reported with one-sigma standard deviations in parentheses. Since the fit is performed in $\ln k_0$, the uncertainty in k_0 becomes asymmetric when converted back to linear space, resulting in non-symmetric upper and lower errors (hence two values in parentheses), whereas Δx is fitted directly in linear space and retains a single symmetric standard deviation.

[1] Oberbarnscheidt, L., Janissen, R., & Oesterhelt, F. (2009). Direct and model free calculation of force-dependent dissociation rates from force spectroscopic data. *Biophysical journal*, *97*(9), L19-L21.

Table S12: List of all 24 tested models during the comparative analysis of the ligand-binding kinetics of G12 and doxycycline.

Model number	model scheme	C_0 [R_1]	C_0 [R_2]
1	$R_1 + D \rightarrow B$	4	-
2	$R_1 \rightleftharpoons R_2 + D \rightarrow B$	2	2
3	$R_2 \rightleftharpoons R_1 + D \rightarrow B$	2	2
4	$R_1 \rightleftharpoons R_2 + D \rightarrow B$<br>$R_1 + D \rightarrow B$	2	2
5	$R_1 + D \rightarrow R_1^* \rightarrow B$	4	-
6	$R_1 + D \rightleftharpoons R_1^* \rightarrow B$	4	-
7	$R_2 \rightleftharpoons R_1 + D \rightarrow R_1^* \rightarrow B$	4	0
8	$R_2 \rightleftharpoons R_1 + D \rightarrow R_1^* \rightarrow B$	0	4
9	$R_2 \rightleftharpoons R_1 + D \rightarrow R_1^* \rightarrow B$	2	2
10	$R_1 \rightleftharpoons R_2 + D \rightarrow R_2^* \rightarrow B$	4	0
11	$R_1 \rightleftharpoons R_2 + D \rightarrow R_2^* \rightarrow B$	0	4
12	$R_1 \rightleftharpoons R_2 + D \rightarrow R_2^* \rightarrow B$	2	2
13	$R_2 \rightleftharpoons R_1 + D \rightarrow R^* \rightarrow B$<br>$R_2 + D \rightarrow R^* \rightarrow B$	4	0
14	$R_2 \rightleftharpoons R_1 + D \rightarrow R^* \rightarrow B$<br>$R_2 + D \rightarrow R^* \rightarrow B$	0	4
15	$R_2 \rightleftharpoons R_1 + D \rightarrow R^* \rightarrow B$<br>$R_2 + D \rightarrow R^* \rightarrow B$	2	2
16	$R_2 \rightleftharpoons R_1 + D \rightarrow R_1^* \rightarrow B$<br>$R_2 + D \rightarrow R_2^* \rightarrow B$	4	0
17	$R_2 \rightleftharpoons R_1 + D \rightarrow R_1^* \rightarrow B$<br>$R_2 + D \rightarrow R_2^* \rightarrow B$	0	4
18	$R_2 \rightleftharpoons R_1 + D \rightarrow R_1^* \rightarrow B$<br>$R_2 + D \rightarrow R_2^* \rightarrow B$	2	2
19	$R_2 \rightleftharpoons R_1 + D \rightleftharpoons R_1^* \rightarrow B$<br>$R_2 + D \rightleftharpoons R_2^* \rightarrow B$	4	0
20	$R_2 \rightleftharpoons R_1 + D \rightleftharpoons R_1^* \rightarrow B$<br>$R_2 + D \rightleftharpoons R_2^* \rightarrow B$	0	4
21	$R_2 \rightleftharpoons R_1 + D \rightleftharpoons R_1^* \rightarrow B$<br>$R_2 + D \rightleftharpoons R_2^* \rightarrow B$	2	2
22	$R_2 \rightleftharpoons R_1 + D \rightleftharpoons R_1^* \rightleftharpoons B$<br>$R_2 + D \rightleftharpoons R_2^* \rightleftharpoons B$	4	0
23	$R_2 \rightleftharpoons R_1 + D \rightleftharpoons R_1^* \rightleftharpoons B$<br>$R_2 + D \rightleftharpoons R_2^* \rightleftharpoons B$	0	4
24	$R_2 \rightleftharpoons R_1 + D \rightleftharpoons R_1^* \rightleftharpoons B$<br>$R_2 + D \rightleftharpoons R_2^* \rightleftharpoons B$	2	2

A symbolic reaction scheme as well as the starting concentration of R_1 and R_2 for each model are given.

Table S13: Summary of the comparative analyses of the ligand binding kinetics of G12.

Model number	SSQ_R	ΔAIC	RMSE
18	1.000	0.0	0.0333886
22	1.011	458.7	0.0335772
17	1.031	1202.0	0.0338940
19	1.031	1207.5	0.0338947
21	1.040	1564.8	0.0340464
16	1.043	1674.3	0.0340947
14	1.047	1825.0	0.0341607
13	1.047	1826.3	0.0341613
20	1.047	1858.9	0.0341718
15	1.060	2321.8	0.0343735
12	1.068	2641.7	0.0345121
24	1.083	3185.3	0.0347413
9	1.089	3396.0	0.0348390
11	1.106	4013.3	0.0351089
23	1.107	4091.7	0.0351372
10	1.108	4150.5	0.0351494
8	1.108	4107.7	0.0351504
6	1.108	4111.1	0.0351528
4	1.113	4257.9	0.0352173
2	1.122	4584.6	0.0353623
3	1.122	4584.6	0.0353623
7	1.154	5709.8	0.0358614
5	1.156	5797.5	0.0359026
1	1.232	8341.1	0.0370643

The obtained RMSE of the fit as well as the relative comparison via SSQ_R (relative sum of squared deviations between experimental data and the theoretical fit) are given. The relative probabilities of the models are reflected by the lowest value of the likelihood criteria ΔAIC.

Table S14: List of determined melting points of the CD thermal melting assay.

Mg^{2+} concentration	CD melting points			
	- Dox		+ Dox	
0 mM	31.3	55.1	25.8	59.8
0.03 mM	29.6	58.8	26.8	60.4
0.5 mM	22.1	59.8	27.8	60.7

Table S15: Oligonucleotide sequences

Name	Sequence (5' – 3')	Manufacturer
Pool_fw	CCAAGTAATACGACTCACTATAGGGCAACTCCAAGCTAGATCTACCGGT	Sigma-Aldrich
Pool_rev	AGTGAAAAGTTCTTCTCCTTTGCTAGCCATTTT	Sigma-Aldrich
Pool_oligo_rev	AGTGAAAAGTTCTTCTCCTTTGCTAGCCATTTTNNNNNNNNNNTAGAAGCCA GTAGNNNNNNNNNNNNNNNNNNNNNNNNNNNNNNNNNNNNNNNNNACCGG TAGATCTAGCTTGGAGTTGCCC	Microsynth
Yeast-HR_fw	CTCGTCATTGTTCTCGTTCCCTTTCTTCCTTGTTTCTTTTTCTGCACAATATTT CAAGCTATACCAAGCATACAATCAACTCCAAGCTAGATCTACCGGT	Sigma-Aldrich
Yeast-HR_rev	CACCCTCTCCACTGACAGAAAATTTGTGCCCATTAACATCACCATCTAATTC AACAAGAATTGGGACAACTCCAGTGAAAAGTTCTTCTCCTTTGCTAGC	Sigma-Aldrich
NGS-index_S1	CCTT**CGTAGCTT**GGGCAACTCCAAGCTAGATCTACCGGT	Sigma-Aldrich
NGS-index_S2	CCTT**CGTGCCAA**GGGCAACTCCAAGCTAGATCTACCGGT	Sigma-Aldrich
NGS-index_S3	CCTT**CTAGTGCG**GGGCAACTCCAAGCTAGATCTACCGGT	Sigma-Aldrich
NGS-index_S4	CCTT**CTCAGCCT**GGGCAACTCCAAGCTAGATCTACCGGT	Sigma-Aldrich
NGS-index_S5	CCTT**CTCTGACA**GGGCAACTCCAAGCTAGATCTACCGGT	Sigma-Aldrich
NGS-index_S6	CCTT**CTCTTCTA**GGGCAACTCCAAGCTAGATCTACCGGT	Sigma-Aldrich
NGS-index_S7	CCTT**CTGATGAA**GGGCAACTCCAAGCTAGATCTACCGGT	Sigma-Aldrich
NGS-index_S8	CCTT**CTGTATTC**GGGCAACTCCAAGCTAGATCTACCGGT	Sigma-Aldrich
NGS-index_S9	CCTT**CTTCGCTC**GGGCAACTCCAAGCTAGATCTACCGGT	Sigma-Aldrich
NGS-index_S10	CCTT**GAACTCAA**GGGCAACTCCAAGCTAGATCTACCGGT	Sigma-Aldrich
NGS-index_C1	CCTT**CGAGAGCA**GGGCAACTCCAAGCTAGATCTACCGGT	Sigma-Aldrich
NGS-index_C2	CCTT**CGCCAGTA**GGGCAACTCCAAGCTAGATCTACCGGT	Sigma-Aldrich
NGS-index_C3	CCTT**CGCCTCAA**GGGCAACTCCAAGCTAGATCTACCGGT	Sigma-Aldrich
NGS-index_C4	CCTT**CGCGGAAT**GGGCAACTCCAAGCTAGATCTACCGGT	Sigma-Aldrich
NGS-index_C5	CCTT**CGCGTTAC**GGGCAACTCCAAGCTAGATCTACCGGT	Sigma-Aldrich
NGS-index_C6	CCTT**CGGAAGGA**GGGCAACTCCAAGCTAGATCTACCGGT	Sigma-Aldrich
NGS-index_C7	CCTT**CGGTGAGA**GGGCAACTCCAAGCTAGATCTACCGGT	Sigma-Aldrich
G12_fw	CCGGTAATTTAGGTCATTGAGTGAACAGACGTCAAGCTAGTTGGGCTACTG GCTTCTAAAACACAGGTAAAATGG	Sigma-Aldrich
G12_rev	CTAGCCATTTTACCTGTGTTTTAGAAGCCAGTAGCCCAACTAGCTTGACGTC TGTTCACTCAATGACCTAAATTA	Sigma-Aldrich
G12_M1_fw	CCGGTAATTTAGGTCATTGAGTGAACAGACGTCAAGCTAGTTGGGCTACTG GCTTCTAAATTACAGGTAAAATGG	Sigma-Aldrich
G12_M1_rev	CTAGCCATTTTACCTGTAATTTAGAAGCCAGTAGCCCAACTAGCTTGACGTC TGTTCACTCAATGACCTAAATTA	Sigma-Aldrich
G12_M2_fw	CCGGTAATTTAGGTCATTGAGTGAACAGACGTCAAGCTAGTTGGGCTACTG GCTTCTAAAACACCGGTAAAATGG	Sigma-Aldrich
G12_M2_rev	CTAGCCATTTTACCGGTGTTTTAGAAGCCAGTAGCCCAACTAGCTTGACGTC TGTTCACTCAATGACCTAAATTA	Sigma-Aldrich
G12_M3_fw	CCGGTCAATAGGTCATTGAGTGAACAGACGTCAAGCTAGTTGGGCTACTGG CTTCTATTGAAAAATGG	Sigma-Aldrich
G12_M3_rev	CTAGCCATTTTTCAATAGAAGCCAGTAGCCCAACTAGCTTGACGTCTGTTCA CTCAATGACCTATTGA	Sigma-Aldrich
G12_M4_fw	CCGGTAATTTAGGTCATTGAGTGAACAGACGTCAAGCTAGTAAAGACTGGC TTCTAAAACACAGGTAAAATGG	Sigma-Aldrich
G12_M4_rev	CTAGCCATTTTACCTGTGTTTTAGAAGCCAGTCTTTACTAGCTTGACGTCTG TTCACTCAATGACCTAAATTA	Sigma-Aldrich
G12_M5_fw	CCGGTAATTTAGGTCATTGAGTGAACAGACGTCAAGCTAGTTTCGACTGGC TTCTAAAACACAGGTAAAATGG	Sigma-Aldrich
G12_M5_rev	CTAGCCATTTTACCTGTGTTTTAGAAGCCAGTCGAAACTAGCTTGACGTCTG TTCACTCAATGACCTAAATTA	Sigma-Aldrich
G12_M6_fw	CCGGTAATTTAGGTCATTGAGTGAACAGACGTCAAGCAGTTGGGCTACTGC TTCTAAAACACAGGTAAAATGG	Sigma-Aldrich
G12_M6_rev	CTAGCCATTTTACCTGTGTTTTAGAAGCAGTAGCCCAACTGCTTGACGTCTG TTCACTCAATGACCTAAATTA	Sigma-Aldrich
G12_M7_fw	CCGGTAATTTAGGTCATTGAGTGAACAGACGTCAGCAGTTGGGCTACTGCT CTAAAACACAGGTAAAATGG	Sigma-Aldrich
G12_M7_rev	CTAGCCATTTTACCTGTGTTTTAGAGCAGTAGCCCAACTGCTGACGTCTGTT CACTCAATGACCTAAATTA	Sigma-Aldrich
G12_M8_fw	CCGGTAATTTAGGTCATTGAGTGAACAGACGTCAGCGTTGGGCTACGCTCT AAAACACAGGTAAAATGG	Sigma-Aldrich

G12_M8_rev	CTAGCCATTTTACCTGTGTTTTAGAGCGTAGCCCAACGCTGACGTCTGTTCACTCAATGACCTAAATTA	Sigma-Aldrich
G12_M9_fw	CCGGTAATTTAGGTCATTGAGTGAACAGACGTCAACGTAGTTGGGCTACTGGCTTCTAAAACACAGGTAAAATGG	Sigma-Aldrich
G12_M9_rev	CTAGCCATTTTACCTGTGTTTTAGAAGCCAGTAGCCCAACTACGTTGACGTCTGTTCACTCAATGACCTAAATTA	Sigma-Aldrich
G12_M10_fw	CCGGTAATTTAGGTCATTGAGTGAACAGACGTCAACGTAGTTGGGCTACTGCGTTCTAAAACACAGGTAAAATGG	Sigma-Aldrich
G12_M10_rev	CTAGCCATTTTACCTGTGTTTTAGAACGCAGTAGCCCAACTACGTTGACGTCTGTTCACTCAATGACCTAAATTA	Sigma-Aldrich
G12_M11_fw	CCGGTCAATAGGTCATTGAGTGAACAGACGTCAGACCGATTGGGCTATCGGTCTCTATTGAAAAATGG	Sigma-Aldrich
G12_M11_rev	CTAGCCATTTTTCAATAGAGACCGATAGCCCAATCGGTCTGACGTCTGTTCACTCAATGACCTATTGA	Sigma-Aldrich
G12_M12_fw	CCGGTCAATAGGTCATTGAGTGAACAGACGTCAAGCTACGTTGGGCTACGTGGCTTCTATTGAAAAATGG	Sigma-Aldrich
G12_M12_rev	CTAGCCATTTTTCAATAGAAGCCACGTAGCCCAACGTAGCTTGACGTCTGTTCACTCAATGACCTATTGA	Sigma-Aldrich
G12_M13_fw	CCGGTCAATAGGTCATTGAGTGAACAGACGTCAAGCTACAGTTGGGCTACTGTGGCTTCTATTGAAAAATGG	Sigma-Aldrich
G12_M13_rev	CTAGCCATTTTTCAATAGAAGCCACAGTAGCCCAACTGTAGCTTGACGTCTGTTCACTCAATGACCTATTGA	Sigma-Aldrich
G12_M14_fw	CCGGTCAATAGGTCATTGAGTGAACAGACAAAAAGCTAGTTGGGCTACTGGCTTCTATTGAAAAATGG	Sigma-Aldrich
G12_M14_rev	CTAGCCATTTTTCAATAGAAGCCAGTAGCCCAACTAGCTTTTTGTCTGTTCACTCAATGACCTATTGA	Sigma-Aldrich
G12_M15_fw	CCGGTCAATAGCTGATTGAGTGAACACAGGTCAAGCTAGTTGGGCTACTGGCTTCTATTGAAAAATGG	Sigma-Aldrich
G12_M15_rev	CTAGCCATTTTTCAATAGAAGCCAGTAGCCCAACTAGCTTGACCTGTGTTCACTCAATCAGCTATTGA	Sigma-Aldrich
G12_M16_fw	CCGGTCAATAGGACATTGAGTGAACAGACGTCAAGCTAGTTGGGCTACTGGCTTCTATTGAAAAATGG	Sigma-Aldrich
G12_M16_rev	CTAGCCATTTTTCAATAGAAGCCAGTAGCCCAACTAGCTTGACGTCTGTTCACTCAATGTCCTATTGA	Sigma-Aldrich
G12_M17_fw	CCGGTCAATAGGACATTGAGTGAACAGTCGTCAAGCTAGTTGGGCTACTGGCTTCTATTGAAAAATGG	Sigma-Aldrich
G12_M17_rev	CTAGCCATTTTTCAATAGAAGCCAGTAGCCCAACTAGCTTGACGACTGTTCACTCAATGTCCTATTGA	Sigma-Aldrich
G12_M18_fw	CCGGTCAATAGGAGATTGAGTGAACACACGTCAAGCTAGTTGGGCTACTGGCTTCTATTGAAAAATGG	Sigma-Aldrich
G12_M18_rev	CTAGCCATTTTTCAATAGAAGCCAGTAGCCCAACTAGCTTGACGTGTGTTCACTCAATCTCCTATTGA	Sigma-Aldrich
G12_M19_fw	CCGGTCAATAGGAGATTGAGTGAACACTCGTCAAGCTAGTTGGGCTACTGGCTTCTATTGAAAAATGG	Sigma-Aldrich
G12_M19_rev	CTAGCCATTTTTCAATAGAAGCCAGTAGCCCAACTAGCTTGACGAGTGTTCACTCAATCTCCTATTGA	Sigma-Aldrich
G12_M20_fw	CCGGTCAATAGGTCCAAGAGTGAACAGACGTCAAGCTAGTTGGGCTACTGGCTTCTATTGAAAAATGG	Sigma-Aldrich
G12_M20_rev	CTAGCCATTTTTCAATAGAAGCCAGTAGCCCAACTAGCTTGACGTCTGTTCACTCTTGGACCTATTGA	Sigma-Aldrich
G12_M21_fw	CCGGTCAATAGGTCATTCAATGAACAGACGTCAAGCTAGTTGGGCTACTGGCTTCTATTGAAAAATGG	Sigma-Aldrich
G12_M21_rev	CTAGCCATTTTTCAATAGAAGCCAGTAGCCCAACTAGCTTGACGTCTGTTCATTGAATGACCTATTGA	Sigma-Aldrich
G12_M22_fw	CCGGTCAATAGGTCATTGAGCAAACAGACGTCAAGCTAGTTGGGCTACTGGCTTCTATTGAAAAATGG	Sigma-Aldrich
G12_M22_rev	CTAGCCATTTTTCAATAGAAGCCAGTAGCCCAACTAGCTTGACGTCTGTTTGCTCAATGACCTATTGA	Sigma-Aldrich
G12_M23_fw	CCGGTCAATAGGTCATTGAGTGACAAGACGTCAAGCTAGTTGGGCTACTGGCTTCTATTGAAAAATGG	Sigma-Aldrich
G12_M23_rev	CTAGCCATTTTTCAATAGAAGCCAGTAGCCCAACTAGCTTGACGTCTTGTCACTCAATGACCTATTGA	Sigma-Aldrich
G12_S1_fw	CCGGTCAACGGGTCATTGAGTGAACAGACGTCAAGCTAGTTGGGCTACTGGCTTCCGTTGAAAAATGG	Sigma-Aldrich
G12_S1_rev	CTAGCCATTTTTCAACGGAAGCCAGTAGCCCAACTAGCTTGACGTCTGTTCACTCAATGACCCGTTGA	Sigma-Aldrich
G12_S2_fw	CCGGTCGATAGGTCATTGAGTGAACAGACGTCAAGCTAGTTGGGCTACTGGCTTCTATCGAAAAATGG	Sigma-Aldrich
G12_S2_rev	CTAGCCATTTTTCGATAGAAGCCAGTAGCCCAACTAGCTTGACGTCTGTTCACTCAATGACCTATCGA	Sigma-Aldrich
G12_S3_fw	CCGGTCGCTAGGTCATTGAGTGAACAGACGTCAAGCTAGTTGGGCTACTGGCTTCTAGCGAAAAATGG	Sigma-Aldrich

G12_S3_rev	CTAGCCATTTTTCGCTAGAAGCCAGTAGCCCAACTAGCTTGACGTCTGTTCA CTCAATGACCTAGCGA	Sigma-Aldrich
G12_S4_fw	CCGGTCGCTAGGTCATTGAGTGAACAGACGTCAAGCTAGTTGGGCTACTGG CTTCTAGTGAAAAATGG	Sigma-Aldrich
G12_S4_rev	CTAGCCATTTTTCACTAGAAGCCAGTAGCCCAACTAGCTTGACGTCTGTTCA CTCAATGACCTAGCGA	Sigma-Aldrich
G12_S5_fw	CCGGTCGATAGGTCATTGAGTGAACAGACGTCAAGCTAGTTGGGCTACTGG CTTCTATTGAAAAATGG	Sigma-Aldrich
G12_S5_rev	CTAGCCATTTTTCAATAGAAGCCAGTAGCCCAACTAGCTTGACGTCTGTTCA CTCAATGACCTATCGA	Sigma-Aldrich
G12_M3_random-template	CAGTGAAAAGTTCTTCTCCTTTGCTAGCCATTTTTCAATAGAAGCCAGTAGC CCAACTAGCTTGACGTCTGTTCACTCAATGACCTATTGACCGGTAGATCTAG CTTGGAGTT	Microsynth
Tetracyc_fw	CCGGTCGGCCTAAAACATACCAGATCGCCACCCGCGCTTTAATCTGGAGAG GTGAAGAATACGACCACCTAGGCCAAAATGG	Sigma-Aldrich
Tetracyc_rev	CTAGCCATTTTGGCCTAGGTGGTCGTATTCTTCACCTCTCCAGATTAAAGCG CGGGTGGCGATCTGGTATGTTTTAGGCCGA	Sigma-Aldrich
Tc-G6_fw	CCGGTCAGTAATCTGCAAACATACCAGATCGCCACCCGCGCTTTAATCTGG AGAGGTGAAGAATACGACCACCGCAGATTACTAAAATGG	Sigma-Aldrich
Tc-G6_rev	CTAGCCATTTTAGTAATCTGCGGTGGTCGTATTCTTCACCTCTCCAGATTAA AGCGCGGGTGGCGATCTGGTATGTTTGCAGATTACTGA	Sigma-Aldrich
Neo_M4_fw	CCGGTCCGGCATAGCTTGTCCTTTAATGGTCCTATGTCGAAAATGG	Sigma-Aldrich
Neo_M4_rev	CTAGCCATTTTCGACATAGGACCATTAAAGGACAAGCTATGCCGGA	Sigma-Aldrich
Tobra_fw	CCGGTGTTTCGGAAAGTTGGACCTACTGGCTTCTACCGAGCACTTAAAATG G	Sigma-Aldrich
Tobra_rev	CTAGCCATTTTAAGTGCTCGGTAGAAGCCAGTAGGTCCAACTTTCCGAAAC A	Sigma-Aldrich
Tobra_N6-G5_fw	CCGGTGTTTCGGAAACTGCGGTTCTACCGAGCACTAAAATGG	Sigma-Aldrich
Tobra_N6-G5_rev	CTAGCCATTTTAGTGCTCGGTAGAACCGCAGTTTCCGAAACA	Sigma-Aldrich
T7_fw	CGAATTCCAAGTAATACGACTCACTATAGG	Sigma-Aldrich
G12_IVT	CATTTTACCTGTGTTTTAGAAGCCAGTAGCCCAACTAGCTTGACGTCTGTTC ACTCAATGACCTAAATTACCGGTAGATCTAGCTTGGAGTTGCCCTATAGTGA GTCGTATTACTTGGAATTCG	Sigma-Aldrich
G12_M3_IVT	TCAATAGAAGCCAGTAGCCCAACTAGCTTGACGTCTGTTCACTCAATGACCT ATTGACCCTATAGTGAGTCGTATTACTTGGAATTCG	Sigma-Aldrich
G12_S1_IVT	TCAACGGAAGCCAGTAGCCCAACTAGCTTGACGTCTGTTCACTCAATGACC CGTTGACCCTATAGTGAGTCGTATTACTTGGAATTCG	Sigma-Aldrich
C01_IVT	CATTTTAGGTTAAACGTAGAAGCCAGTAGTATGTCTTGTCTCAGCACAGCTG ATGGGCGCCTAACCTGAACCGGTAGATCTAGCTTGGAGTTGCCCTATAGTG AGTCGTATTACTTGGAATTCG	Sigma-Aldrich
Dox1_splice_fw	CGGAAGGTCTCCAGTGTTTCTCTTTTTTTCAGGAACTGGTCATTGAGTGAAC AGACGTCAAGCTAGTTGG	Sigma-Aldrich
Dox1_splice_rev	GCCTTGGTCTCTTGGTTGTTTGGTTGTTTCAGGAACTGAAGCCAGTAGCCC AACTAGCTTGACGTCTG	Sigma-Aldrich
ON-control_fw	AGCTCGGTCTCGTTCATCAACAACCATTGACGTATTAGCATAGGCGCTCTA GCTCTGAGGAGACCTGACTG	Sigma-Aldrich
ON-control_rev	CAGTCAGGTCTCCTCAGAGCTAGAGCGCCTATGCTAATACGTCAATGGTTG TTGATGAACGAGACCGAGCT	Sigma-Aldrich
Dox3_GFP-splice_fw	AGCTCGGTCTCGTTCAGGCGCCGGTCATTGAGTGAACAGACGTCAAGCTA GTTGGGCTACTGGCTTCGGCGCCTGAGGAGACCTGACTG	Sigma-Aldrich
Dox3_GFP-splice_rev	CAGTCAGGTCTCCTCAGGCGCCGAAGCCAGTAGCCCAACTAGCTTGACGT CTGTTCACTCAATGACCGGCGCCTGAACGAGACCGAGCT	Sigma-Aldrich
DNA-handle_ss-overhang	CCTCCCGCACCACTCCAACTACGTCAGCCCTGCC	Sigma-Aldrich
T7-SMFS_fw	CGAATTCCAAGTAATACGACTCACTATAGGCAGGGCUGACGUAGUUGGAG UG	Sigma-Aldrich
Tc aptamer	CACCTGAATTCTGCCCTCACTATATAGTAATCTGCAGGTGGTCGTATTCTTC ACCTCTCCAGATTTTCATCTGGTATGTTTTGCAGATTACTATATCCTCCCGCA CCACTCCAACTACGTCAGCCCTGCC	Sigma-Aldrich
G12_long-stem	CACCTGAATTCTGCCCTCACTGGAGTAATCTCGAAGCCAGTAGCCCAACTA GCTTGACGTCTGTTCACTCAATGACCGAGATTACTCCCCTCCCGCACCACT CCAACTACGTCAGCCCTGCC	Sigma-Aldrich
G12_short-stem	CACCTGAATTCTGCCCTCACTCCGTGAAGCCAGTAGCCCAACTAGCTTGAC GTCTGTTCACTCAATGACCACGGCCTCCCGCACCACTCCAACTACGTCAGC CCTGCC	Sigma-Aldrich
G12_no-stem	CACCTGAATTCTGCCCTCACTAAGCCAGTAGCCCAACTAGCTTGACGTCTG TTCACTCAATGACCCTCCCGCACCACTCCAACTACGTCAGCCCTGCC	Sigma-Aldrich

Positions labelled as “N” indicate a random distribution between all four nucleobases (A, T, G, C). Barcodes used for NGS multiplexing are indicated in **bold**. For the “G12_M3_random-template” used for the randomized mutation screening, the partly randomized section (4,5% mutation rate/nucleotide) is indicated as underlined.

Table S16: Plasmid sequences

**Plasmid**, description	**Sequence**
**pCBB05**,<br>positive control for the expression of GFP in yeast	tcgcgcgtttcggtgatgacggtgaaaacctctgacacatgcagctcccggagacggtcacagcttgtctgtaagcggatgccggg<br>agcagacaagcccgtcagggcgcgtcagcgggtgttggcgggtgtcggggctggcttaactatgcggcatcagagcagattgtact<br>gagagtgcaccataccacagcttttcaattcaattcatcatttttttttattcttttttttgatttcggtttctttgaaatttttttgattcggtaatctcc<br>gaacagaaggaagaacgaaggaaggagcacagacttagattggtatatatacgcatatgtagtgttgaagaaacatgaaattgcc<br>cagtattcttaacccaactgcacagaacaaaaacctgcaggaaacgaagataaatcatgtcgaaagctacatataaggaacgtg<br>ctgctactcatcctagtcctgttgctgccaagctatttaatatcatgcacgaaaagcaaacaaacttgtgtgcttcattggatgttcgtacc<br>accaaggaattactggagttagttgaagcattaggtcccaaaatttgtttactaaaaacacatgtggatatcttgactgatttttccatgg<br>agggcacagttaagccgctaaaggcattatccgccaagtacaattttttactcttcgaagacagaaaatttgctgacattggtaataca<br>gtcaaattgcagtactctgcgggtgtatacagaatagcagaatgggcagacattacgaatgcacacggtgtggtgggcccaggtatt<br>gttagcggtttgaagcaggcggcagaagaagtaacaaaggaacctagaggccttttgatgttagcagaattgtcatgcaagggctc<br>cctatctactggagaatatactaagggtactgttgacattgcgaagagcgacaaagattttgttatcggctttattgctcaaagagacat<br>gggtggaagagatgaaggttacgattggttgattatgacacccggtgtgggtttagatgacaagggagacgcattgggtcaacagta<br>tagaaccgtggatgatgtggtctctacaggatctgacattattattgttggaagaggactatttgcaaagggaagggatgctaaggtag<br>agggtgaacgttacagaaaagcaggctgggaagcatatttgagaagatgcggccagcaaaactaaaaaactgtattataagtaa<br>atgcatgtatactaaactcacaaattagagcttcaatttaattatatcagttattaccctatgcggtgtgaaataccgcacagatgcgtaa<br>ggagaaaataccgcatcaggaaattgtaaacgttaatattttgttaaaattcgcgttaaatttttgttaaatcagctcattttttaaccaata<br>ggccgaaatcggcaaaatcccttataaatcaaaagaatagaccgagatagggttgagtgttgttccagtttggaacaagagtccact<br>attaaagaacgtggactccaacgtcaaagggcgaaaaaccgtctatcagggcgatggcccactacgtgaaccatcacc<br>aagcaacctgacctacaggaaagagttactcaagaataagaattttcgttttaaaacctaagagtcactttaaaatttgtatacacttatt<br>ttttttataacttatttaataataaaaatcataaatcataagaaattcgcttatttagaagtgtcaacaacgctacttgtacagctcgtccatg<br>ccgccggtggagtggcggccctcggcgcgttcgtactgttccacgatggtgtagtcctcgttgtgggaggtgatgtccaacttgatgttg<br>acgttgtaggcgccgggcagctgcacgggcttcttggccttgtaggtggtcttgacctcagcgtcgtagtggccgccgtccttcagcttc<br>agcctctgcttgatctcgcccttcagggcgccgtcctcggggtacatccgctcggaggaggcctcccagcccatggtcttcttctgcatt<br>acggggccgtcggaggggaagttggtgccgcgcagcttcaccttgtagatgaactcgccgtcctgcagggaggagtcctgggtca<br>cggtcaccacgccgccgtcctcgaagttcatcacgcgctcccacttgaagccctcggggaaggacagcttcaagtagtcggggat<br>gtcggcggggtgcttcacgtaggccttggagccgtacatgaactgaggggacaggatgtcccaggcgaagggcagggggccac<br>ccttggtcaccttcagcttggcggtctgggtgccctcgtaggggcggccctcgccctcgccctcgatctcgaactcgtggccgttcacg<br>gagccctccatgtgcaccttgaagcgcatgaactccttgatgatggccatgttatcctcctcgcccttgctcaccatttttctagaaaactt<br>agattagattgctatgctttctttctaatgagcaagaggtaaaaaaagttgtagtagagcaagaaaagtgggactgaaacttgggaa<br>attgaaggccgtttattaacttaaatatcgatgggaggtcatcgaaagagaaaaaaatcaaaaaaagaatttcaagaaaaagaa<br>acgtgataaaaattttttattgccttttcgacgaagaaaaagaaacgaggcggtctcttttttcttttccaaacctttagtacgggtaattaa<br>cgacaccctagaggaagaaagaggggaaatttagtatgctgtgcttgggtgttttgaagtggtacggcgatgcgcggagtccgaga<br>agatctggaagagtaaaagaggagtagaaacgtttcgaagctatgcataggccactagtcgcatgcaacttcttttcttttttttttctttct<br>ctctcccccgttgttgtctcaccatatccgcaatgacaaaaaaaatgatggaagacactaaaggaaaaaattaacgacaaagacag<br>caccaacagatgtcgttgttccagagctgatgaggggtatctcgaagcacacgaaactttttccttccttcattcacgcacactactctct<br>aatgagcaacggtatacggccttccttccagttacttgaatttgaaataaaaaaaagtttgctgtcttgctatcaagtataaatagacctg<br>caattattaatcttttgtttcctcgtcattgttctcgttccctttcttccttgtttctttttctgcacaatatttcaagctataccaagcatacaatcaa<br>ctccaagctagatctcttaagatggctagcaaaggagaagaacttttcactggagttgtcccaattcttgttgaattagatggtgatgtta<br>atgggcacaaattttctgtcagtggagagggtgaaggtgatgctacatacggaaagcttacccttaaatttatttgcactactggaaaa<br>ctacctgttccatggccaacacttgtcactactttgacctatggtgttcaatgcttttcccgttatccggatcatatgaaacggcatgactttttt<br>caagagtgccatgcccgaaggttatgtacaggaacgcactatatctttcaaagatgacgggaactacaagacgcgtgctgaagtc<br>aagtttgaaggtgatacccttgttaatcgtatcgagttaaaaggtattgattttaaagaagatggaaacattctcggacacaaactcga<br>gtacaactataactcacacaatgtatacatcacggcagacaaacaaaagaatggaatcaaagctaacttcaaaattcgccacaac<br>attgaagatggatccgttcaactagcagaccattatcaacaaaatactccaattggcgatggccctgtccttttaccagacaaccatta<br>cctgtcgacmcaatctgccctttcgaaagatcccaacgaaaagcgtgaccacatggtccttcttgagtttgtaactgctgctgggatta<br>cacatggcatggatgagctctacaaataatgaattcgagcatttaaatctagacgagctcctcgagcctgcagcagctgaagctttgg<br>acttcttcgccagaggtttggtcaagtctccaatcaaggttgtcggcttgtctaccttgccagaaatttacgaaaagatggaaaagggt<br>caaatcgttggtagatacgttgttgacacttctaaataagcgaatttcttatgatttatgatttttattattaaataagttataaaaaaaaataa<br>gtgtatacaaattttaaagtgactcttaggttttaaaacgaaaattcttattcttgagtaactctttcctgtaggtcaggttgctttctcaggtat<br>agcatgaggtcgctcttattgaccacacctctaccggcatgcagctgtttcctgtgtgaaattgttatccgctcacaattccacacaacat<br>acgagccggaagcataaagtgtaaagcctggggtgcctaatgagtgagctaactcacattaattgcgttgcgctcactgcccgctttc<br>cagtcgggaaacctgtcgtgccagctgcattaatgaatcggccaacgcgcggggagaggcggtttgcgtattgggcgctcttccgct<br>tcctcgctcactgactcgctgcgctcggtcgttcggctgcggcgagcggtatcagctcactcaaaggcggtaatacggttatccacag<br>aatcaggggataacgcaggaaagaacatgtgagcaaaaggccagcaaaaggccaggaaccgtaaaaaggccgcgttgctgg<br>cgtttttccataggctccgcccccctgacgagcatcacaaaaatcgacgctcaagtcagaggtggcgaaacccgacaggactata<br>aagataccaggcgtttccccctggaagctccctcgtgcgctctcctgttccgaccctgccgcttaccggatacctgtccgcctttctccct<br>tcgggaagcgtggcgctttctcatagctcacgctgtaggtatctcagttcggtgtaggtcgttcgctccaagctgggctgtgtgcacgaa<br>ccccccgttcagcccgaccgctgcgccttatccggtaactatcgtcttgagtccaacccggtaagacacgacttatcgccactggca<br>gcagccactggtaacaggattagcagagcgaggtatgtaggcggtgctacagagttcttgaagtggtggcctaactacggctacac<br>tagaaggacagtatttggtatctgcgctctgctgaagccagttaccttcggaaaaagagttggtagctcttgatccggcaaacaaacc<br>accgctggtagcggtggtttttttgtttgcaagcagcagattacgcgcagaaaaaaaggatctcaagaagatcctttgatcttttctacg<br>gggtctgacgctcagtggaacgaaaactcacgttaagggattttggtcatgagattatcaaaaaggatcttcacctagatccttttaaat<br>taaaaatgaagttttaaatcaatctaaagtatatatgagtaaacttggtctgacagttaccaatgcttaatcagtgaggcacctatctca<br>gcgatctgtctatttcgttcatccatagttgcctgactccccgtcgtgtagataactacgatacgggagggcttaccatctggccccagtg<br>ctgcaatgataccgcgagacccacgctcaccggctccagatttatcagcaataaaccagccagccggaagggccgagcgcaga<br>agtggtcctgcaactttatccgcctccatccagtctattaattgttgccgggaagctagagtaagtagttcgccagttaatagtttgcgca<br>acgttgttgccattgctacaggcatcgtggtgtcacgctcgtcgtttggtatggcttcattcagctccggttcccaacgatcaaggcgagt<br>tacatgatcccccatgttgtgcaaaaaagcggttagctccttcggtcctccgatcgttgtcagaagtaagttggccgcagtgttatcact<br>catggttatggcagcactgcataattctcttactgtcatgccatccgtaagatgcttttctgtgactggtgagtactcaaccaagtcattctg<br>agaatagtgtatgcggcgaccgagttgctcttgcccggcgtcaatacgggataataccgcgccacatagcagaactttaaaagtgct<br>catcattggaaaacgttcttcggggcgaaaactctcaaggatcttaccgctgttgagatccagttcgatgtaacccactcgtgcaccca

	actgatcttcagcatcttttactttcaccagcgtttctgggtgagcaaaaacaggaaggcaaaatgccgcaaaaaagggaataagg<br>gcgacacggaaatgttgaatactcatactcttcctttttcaatattattgaagcatttatcagggttattgtctcatgagcggatacatatttg<br>aatgtatttagaaaaataaacaaataggggttccgcgcacatttccccgaaaagtgccacctgggtcctttcatcacgtgctataaaa<br>ataattataatttaaattttttaatataaatatataaattaaaaatagaaagtaaaaaaagaaattaaagaaaaaatagtttttgttttccg<br>aagatgtaaaagactctagggggatcgccaacaaatactaccttttatcttgctcttcctgctctcaggtattaatgccgaattgtttcatct<br>tgtctgtgtagaagaccacacacgaaaatcctgtgattttacattttacttatcgttaatcgaatgtatatctatttaatctgcttttcttgtctaa<br>taaatatatatgtaaagtacgctttttgttgaaattttttaaacctttgtttatttttttttcttcattccgtaactcttctaccttctttatttactttctaaa<br>atccaaatacaaaacataaaaataaataaacacagagtaaattcccaaattattccatcattaaaagatacgaggcgcgtgtaagtt<br>acaggcaagcgatccgtcctaagaaaccattattatcatgacattaacctataaaaataggcgtatcacgaggccctttcgtc
**pCBB06**,<br>plasmid used as negative control for the GFP expression in yeast and for testing the regulatory potential of aptamers in yeast	tcgcgcgtttcggtgatgacggtgaaaacctctgacacatgcagctcccggagacggtcacagcttgtctgtaagcggatgccggg<br>agcagacaagcccgtcagggcgcgtcagcgggtgttggcgggtgtcggggctggcttaactatgcggcatcagagcagattgtact<br>gagagtgcaccataccacagcttttcaattcaattcatcattttttttttattcttttttttgatttcggtttctttgaaatttttttgattcggtaatctcc<br>gaacagaaggaagaacgaaggaaggagcacagacttagattggtatatatacgcatatgtagtgttgaagaaacatgaaattgcc<br>cagtattcttaacccaactgcacagaacaaaaacctgcaggaaacgaagataaatcatgtcgaaagctacatataaggaacgtg<br>ctgctactcatcctagtcctgttgctgccaagctatttaatatcatgcacgaaaagcaaacaaacttgtgtgcttcattggatgttcgtacc<br>accaaggaattactggagttagttgaagcattaggtcccaaaatttgtttactaaaaacacatgtggatatcttgactgatttttccatgg<br>agggcacagttaagccgctaaaggcattatccgccaagtacaattttttactcttcgaagacagaaaatttgctgacattggtaataca<br>gtcaaattgcagtactctgcgggtgtatacagaatagcagaatgggcagacattacgaatgcacacggtgtggtgggcccaggtatt<br>gttagcggtttgaagcaggcggcagaagaagtaacaaaggaacctagaggccttttgatgttagcagaattgtcatgcaagggctc<br>cctatctactggagaatatactaagggtactgttgacattgcgaagagcgacaaagattttgttatcggctttattgctcaaagagacat<br>gggtggaagagatgaaggttacgattggttgattatgacacccggtgtgggtttagatgacaagggagacgcattgggtcaacagta<br>tagaaccgtggatgatgtggtctctacaggatctgacattattattgttggaagaggactatttgcaaagggaagggatgctaaggtag<br>agggtgaacgttacagaaaagcaggctgggaagcatatttgagaagatgcggccagcaaaactaaaaaactgtattataagtaa<br>atgcatgtatactaaactcacaaattagagcttcaatttaattatatcagttattaccctatgcggtgtgaaataccgcacagatgcgtaa<br>ggagaaaataccgcatcaggaaattgtaaacgttaatattttgttaaaattcgcgttaaatttttgttaaatcagctcattttttaaccaata<br>ggccgaaatcggcaaaatcccttataaatcaaaagaatagaccgagatagggttgagtgttgttccagtttggaacaagagtccact<br>attaaagaacgtggactccaacgtcaaagggcgaaaaaccgtctatcaggctcagcgtacgagcgacctcatgctatacctgaga<br>aagcaacctgacctacaggaaagagttactcaagaataagaatttcgttttaaaacctaagagtcactttaaaatttgtatacacttatt<br>ttttttataacttatttaataataaaaatcataaatcataagaaattcgcttatttagaagtgtcaacaacgctacttgtacagctcgtccatg<br>ccgccggtggagtggcggccctcggcgcgttcgtactgttccacgatggtgtagtcctcgttgtgggaggtgatgtccaacttgatgttg<br>acgttgtaggcgccgggcagctgcacgggcttcttggccttgtaggtggtcttgacctcagcgtcgtagtggccgccgtccttcagcttc<br>agcctctgcttgatctcgcccttcagggcgccgtcctcggggtacatccgctcggaggaggcctcccagcccatggtcttcttctgcatt<br>acggggccgtcggaggggaagttggtgccgcgcagcttcaccttgtagatgaactcgccgtcctgcagggaggagtcctgggtca<br>cggtcaccacgccgccgtcctcgaagttcatcacgcgctcccacttgaagccctcggggaaggacagcttcaagtagtcggggat<br>gtcggcggggtgcttcacgtaggccttggagccgtacatgaactgaggggacaggatgtcccaggcgaagggcagggggccac<br>ccttggtcaccttcagcttggcggtctgggtgccctcgtaggggcggccctcgccctcgccctcgatctcgaactcgtggccgttcacg<br>gagccctccatgtgcaccttgaagcgcatgaactccttgatgatggccatgttatcctcctcgcccttgctcaccatttttctagaaaactt<br>agattagattgctatgctttctttctaatgagcaagaggtaaaaaaagttgtagtagagcaagaaaagtgggactgaaacttgggaa<br>attgaaggccgtttattaacttaaatatcgatgggaggtcatcgaaagagaaaaaaatcaaaaaaagaattttcaagaaaaagaa<br>acgtgataaaaatttttattgcctttttcgacgaagaaaaagaaacgaggcggtctctttttttcttttccaaacctttagtacgggtaattaa<br>cgacaccctagaggaagaaagaggggaaatttagtatgctgtgcttgggtgttttgaagtggtacggcgatgcgcggagtccgaga<br>agatctggaagagtaaaagaggagtagaaacgtttcgaagctatgcataggccactagtcgcatgcaacttcttttcttttttttttctttttct<br>ctctcccccgttgttgtctcaccatatccgcaatgacaaaaaaatgatggaagacactaaaggaaaaaattaacgacaaagacag<br>caccaacagatgtcgttgttccagagctgatgaggggtatctcgaagcacacgaaactttttccttccttcattcacgcacactactctct<br>aatgagcaacggtatacggccttccttccagttacttgaatttgaaataaaaaaaagtttgctgtcttgctatcaagtataaatagacctg<br>caattattaatcttttgtttcctcgtcattgttctcgttccctttcttccttgtttctttttctgcacaatatttcaagctataccaagcatacaatcaa<br>ctccaagctagatctaccggtctcttcgctagcaaaggagaagaacttttcactggagttgtcccaattcttgttgaattagatggtgatg<br>ttaatgggcacaaattttctgtcagtggagagggtgaaggtgatgctacatacggaaagcttacccttaaatttatttgcactactggaa<br>aactacctgttccatggccaacacttgtcactactttgacctatggtgttcaatgcttttcccgttatccggatcatatgaaacggcatgact<br>ttttcaagagtgccatgcccgaaggttatgtacaggaacgcactatatctttcaaagatgacgggaactacaagacgcgtgctgaag<br>tcaagtttgaaggtgatacccttgttaatcgtatcgagttaaaaggtattgattttaaagaagatggaaacattctcggacacaaactcg<br>agtacaactataactcacacaatgtatacatcacggcagacaaacaaaagaatggaatcaaagctaacttcaaaattcgccacaa<br>cattgaagatggatccgttcaactagcagaccattatcaacaaaatactccaattggcgatggccctgtccttttaccagacaaccatt<br>acctgtcgacacaatctgccctttcgaaagatcccaacgaaaagcgtgaccacatggtccttcttgagtttgtaactgctgctgggatt<br>acacatggcatggatgagctctacaaataatgaattcgagcatttaaatctagacgagctcctcgagcctgcagcagctgaagctttg<br>gacttcttcgccagaggtttggtcaagtctccaatcaaggttgtcggcttgtctaccttgccagaaatttacgaaaagatggaaaaggg<br>tcaaatcgttggtagatacgttgttgacacttctaaataagcgaatttcttatgatttatgatttttattattaaataagttataaaaaaaataa<br>gtgtatacaaattttaaagtgactcttaggttttaaaacgaaaattcttattcttgagtaactctttcctgtaggtcaggttgctttctcaggtat<br>agcatgaggtcgctcttattgaccacacctctaccggcatgcagctgttccctgtcttattgaccacacctctaccggcatgcagctgttt<br>cctgtgtgaaattgttatccgctcacaattccacacaacatacgagccggaagcataaagtgtaaagcctggggtgcctaatgagtg<br>agctaactcacattaattgcgttgcgctcactgcccgctttccagtcgggaaacctgtcgtgccagctgcattaatgaatcggccaacg<br>cgcggggagaggcggtttgcgtattgggcgctcttccgcttcctcgctcactgactcgctgcgctcggtcgttcggctgcggcgagcg<br>gtatcagctcactcaaaggcggtaatacggttatccacagaatcaggggataacgcaggaaagaacatgtgagcaaaaggcca<br>gcaaaaggccaggaaccgtaaaaaggccgcgttgctggcgtttttccataggctccgcccccctgacgagcatcacaaaaatcga<br>cgctcaagtcagaggtggcgaaacccgacaggactataaagataccaggcgtttccccctggaagctccctcgtgcgctctcctgtt<br>ccgaccctgccgcttaccggatacctgtccgcctttctcccttcgggaagcgtggcgctttctcatagctcacgctgtaggtatctcagtt<br>cggtgtaggtcgttcgctccaagctgggctgtgtgcacgaaccccccgttcagcccgaccgctgcgccttatccggtaactatcgtctt<br>gagtccaacccggtaagacacgacttatcgccactggcagcagccactggtaacaggattagcagagcgaggtatgtaggcggt<br>gctacagagttcttgaagtggtggcctaactacggctacactagaaggacagtatttggtatctgcgctctgctgaagccagttaccttc<br>ggaaaaagagttggtagctcttgatccggcaaacaaaccaccgctggtagcggtggtttttttgtttgcaagcagcagattacgcgca<br>gaaaaaaaggatctcaagaagatcctttgatcttttctacggggtctgacgctcagtggaacgaaaactcacgttaagggattttggtc<br>atgagattatcaaaaaggatcttcacctagatccttttaaattaaaaatgaagttttaaatcaatctaaagtatatatgagtaaacttggtc<br>tgacagttaccaatgcttaatcagtgaggcacctatctcagcgatctgtctatttcgttcatccatagttgcctgactccccgtcgtgtagat<br>aactacgatacgggagggcttaccatctggccccagtgctgcaatgataccgcgagacccacgctcaccggctccagatttatcag

	caataaaccagccagccggaagggccgagcgcagaagtggtcctgcaactttatccgcctccatccagtctattaattgttgccggg<br>aagctagagtaagtagttcgccagttaatagtttgcgcaacgttgttgccattgctacaggcatcgtggtgtcacgctcgtcgtttggtat<br>ggcttcattcagctccggttcccaacgatcaaggcgagttacatgatcccccatgttgtgcaaaaaagcggttagctccttcggtcctc<br>cgatcgttgtcagaagtaagttggccgcagtgttatcactcatggttatggcagcactgcataattctcttactgtcatgccatccgtaag<br>atgcttttctgtgactggtgagtactcaaccaagtcattctgagaatagtgtatgcggcgaccgagttgctcttgcccggcgtcaatacg<br>ggataataccgcgccacatagcagaactttaaaagtgctcatcattggaaaacgttcttcggggcgaaaactctcaaggatcttacc<br>gctgttgagatccagttcgatgtaacccactcgtgcacccaactgatcttcagcatcttttactttcaccagcgtttctgggtgagcaaaa<br>acaggaaggcaaaatgccgcaaaaaagggaataagggcgacacggaaatgttgaatactcatactcttcctttttcaatattattga<br>agcatttatcagggttattgtctcatgagcggatacatatttgaatgtatttagaaaaataaacaaataggggttccgcgcacatttcccc<br>gaaaagtgccacctgggtccttttcatcacgtgctataaaaataattataatttaaattttttaatataaatatataaattaaaaatagaaa<br>gtaaaaaaagaaattaaagaaaaaatagtttttgttttccgaagatgtaaaagactctagggggatcgccaacaaatactaccttttat<br>cttgctcttcctgctctcaggtattaatgccgaattgtttcatcttgtctgtgtagaagaccacacacgaaaatcctgtgattttacattttactt<br>atcgttaatcgaatgtatatctatttaatctgcttttcttgtctaataaatatatatgtaaagtacgctttttgttgaaattttttaaacctttgtttattt<br>ttttttcttcattccgtaactcttctaccttctttatttactttctaaaatccaaatacaaaacataaaaataaataaacacagagtaaattccc<br>aaattattccatcattaaaagatacgaggcgcgtgtaagttacaggcaagcgatccgtcctaagaaaccattattatcatgacattaa<br>cctataaaaataggcgtatcacgaggccctttcgtc
**pRL-SV40,**<br>used as transfection control, constitutively expressing Renilla luciferase	agatctgcgcagcaccatggcctgaaataacctctgaaagaggaacttggttaggtaccttctgaggcggaaagaaccagctgtg<br>gaatgtgtgtcagttagggtgtggaaagtccccaggctccccagcaggcagaagtatgcaaagcatgcatctcaattagtcagcaa<br>ccaggtgtggaaagtccccaggctccccagcaggcagaagtatgcaaagcatgcatctcaattagtcagcaaccatagtcccgcc<br>cctaactccgcccatcccgcccctaactccgcccagttccgcccattctccgccccatggctgactaattttttttatttatgcagaggcc<br>gaggccgcctcggcctctgagctattccagaagtagtgaggaggcttttttggaggcctaggcttttgcaaaaagcttgattcttctgac<br>acaacagtctcgaacttaagctgcagaagttggtcgtgaggcactgggcaggtaagtatcaaggttacaagacaggtttaaggag<br>accaatagaaactgggcttgtcgagacagagaagactcttgcgtttctgataggcacctattggtcttactgacatccactttgcctttct<br>ctccacaggtgtccactcccagttcaattacagctcttaaggctagagtacttaatacgactcactataggctagccaccatgacttcg<br>aaagtttatgatccagaacaaaggaaacggatgataactggtccgcagtggtgggccagatgtaaacaaatgaatgttcttgattca<br>tttattaattattatgattcagaaaaacatgcagaaaatgctgttattttttacatggtaacgcggcctcttcttatttatggcgacatgttgtg<br>ccacatattgagccagtagcgcggtgtattataccagaccttattggtatgggcaaatcaggcaaatctggtaatggttcttataggtta<br>cttgatcattacaaatatcttactgcatggtttgaacttcttaatttaccaaagaagatcatttttgtcggccatgattggggtgcttgtttggc<br>atttcattatagctatgagcatcaagataagatcaaagcaatagttcacgctgaaagtgtagtagatgtgattgaatcatgggatgaat<br>ggcctgatattgaagaagatattgcgttgatcaaatctgaagaaggagaaaaaatggttttggagaataacttcttcgtggaaaccat<br>gttgccatcaaaaatcatgagaaagttagaaccagaagaatttgcagcatatcttgaaccattcaaagagaaaggtgaagttcgtc<br>gtccaacattatcatggcctcgtgaaatcccgttagtaaaaggtggtaaacctgacgttgtacaaattgttaggaattataatgcttatct<br>acgtgcaagtgatgatttaccaaaaatgtttattgaatcggacccaggattcttttccaatgctattgttgaaggtgccaagaagtttccta<br>atactgaatttgtcaaagtaaaaggtcttcatttttcgcaagaagatgcacctgatgaaatgggaaaatatatcaaatcgttcgttgagc<br>gagttctcaaaaatgaacaataattctagagcggccgcttcgagcagacatgataagatacattgatgagtttggacaaaccacaa<br>ctagaatgcagtgaaaaaaatgctttatttgtgaaatttgtgatgctattgctttatttgtaaccattataagctgcaataaacaagttaaca<br>acaacaattgcattcattttatgtttcaggttcagggggaggtgtgggaggttttttaaagcaagtaaaacctctacaaatgtggtaaaat<br>cgataaggatccaggtggcacttttcggggaaatgtgcgcggaacccctatttgtttatttttctaaatacattcaaatatgtatccgctca<br>tgagacaataaccctgataaatgcttcaataatattgaaaaaggaagagtatgagtattcaacatttccgtgtcgcccttattcccttttttt<br>gcggcattttgccttcctgtttttgctcacccagaaacgctggtgaaagtaaaagatgctgaagatcagttgggtgcacgagtgggtta<br>catcgaactggatctcaacagcggtaagatccttgagagttttcgccccgaagaacgttttccaatgatgagcacttttaaagttctgct<br>atgtggcgcggtattatcccgtattgacgccgggcaagagcaactcggtcgccgcatacactattctcagaatgacttggttgagtact<br>caccagtcacagaaaagcatcttacggatggcatgacagtaagagaattatgcagtgctgccataaccatgagtgataacactgc<br>ggccaacttacttctgacaacgatcggaggaccgaaggagctaaccgcttttttgcacaacatgggggatcatgtaactcgccttgat<br>cgttgggaaccggagctgaatgaagccataccaaacgacgagcgtgacaccacgatgcctgtagcaatggcaacaacgttgcg<br>caaactattaactggcgaactacttactctagcttcccggcaacaattaatagactggatggaggcggataaagttgcaggaccactt<br>ctgcgctcggcccttccggctggctggtttattgctgataaatctggagccggtgagcgtgggtctcgcggtatcattgcagcactggg<br>gccagatggtaagccctcccgtatcgtagttatctacacgacggggagtcaggcaactatggatgaacgaaatagacagatcgctg<br>agataggtgcctcactgattaagcattggtaactgtcagaccaagtttactcatatatactttagattgatttaaaacttcatttttaatttaa<br>aaggatctaggtgaagatcctttttgataatctcatgaccaaaatcccttaacgtgagttttcgttccactgagcgtcagaccccgtaga<br>aaagatcaaaggatcttcttgagatcctttttttctgcgcgtaatctgctgcttgcaaacaaaaaaaccaccgctaccagcggtggtttgt<br>ttgccggatcaagagctaccaactctttttccgaaggtaactggcttcagcagagcgcagataccaaatactgttcttctagtgtagcc<br>gtagttaggccaccacttcaagaactctgtagcaccgcctacatacctcgctctgctaatcctgttaccagtggctgctgccagtggcg<br>ataagtcgtgtcttaccgggttggactcaagacgatagttaccggataaggcgcagcggtcgggctgaacggggggttcgtgcaca<br>cagcccagcttggagcgaacgacctacaccgaactgagatacctacagcgtgagctatgagaaagcgccacgcttcccgaagg<br>gagaaaggcggacaggtatccggtaagcggcagggtcggaacaggagagcgcacgagggagcttccagggggaaacgcct<br>ggtatctttatagtcctgtcgggtttcgccacctctgacttgagcgtcgatttttgtgatgctcgtcaggggggcggagcctatggaaaaa<br>cgccagcaacgcggcctttttacggttcctggccttttgctggccttttgctcacatggctcgac
**pWHE237_noTC,**<br>used as negative control, containing a suicide exon without a controllable splice site, leading to constant inhibition of Firefly luciferase	gccattcgccattcaggctgcgcaactgttgggaagggcgatcggtgcgggcctcttcgctattacgccagcccaagctaccatgat<br>aagtaagtaatattaaggtacgggaggtacttggagcggccctagcccgggctcgagatctgcgatcgtaggcgtgtacggtgga<br>ggtctatataagcagagctcgtttagtgaaccgtcagatcgcctggagacgccatccacgctgttttgacctccatagaagacaccg<br>ggaccgatccagcctccgcggccccgaattcaccatggaagacgccaaaaacataaagaaaggcgtgagtctatgggaccctttg<br>atgttttctttccccttcttttctatggttaagttcatgtcataggaaggggagaagtaacagggtacagtttagaatgggaaacagacga<br>atgattgcatcagtgtggaagtctcaggatcgttttagtttcttttatttgctgttcataacaattgttttcttttgtttaattcttgctttctttttttttcttc<br>tccgcaatttttactattatacttaatgccttaacattgtgtataacaaaaggaaatatctctgagatacattaagtaacttaaaaaaaaa<br>ctttacacagtctgcctagtacattactatttggaatatatgtgtgcttatttgcatattcataatctccctactttattttcttttatttttaattgata<br>cataatcattatacatatttatgggttaaagtgtaatgttttaatatgtgtacacatattgaccaaatcagggtaattttgcatttgtaattttaa<br>aaaatgctttcttcttttaatatactttttttgtttatcttatttctaatactttccctaatctctttctttcagggcaataatgatacaatgtatcatgcc<br>tctttgcaccattctaaagaataacagtgataatttctgggttaaggcaatagcaatatttctgcatataaatatttctgcatataaattgta<br>actgatgtaagaggtttcatattgctaatagcagctacaatccagctaccattctgcttttattttatggttgggataaggctggattattctg<br>agtccaagctaggcccttttgctaatcatgttcatacctcttatcttcctcccacagccggcgccattctatccgctggaagatggaaccg<br>ctggagagcaactgcataaggctatgaagagatacgccctggttcctggaacaattgcttttacagatgcacatatcgaggtggaca<br>tcacttacgctgagtacttcgaaatgtccgttcggttggcagaagctatgaaacgatatgggctgaatacaaatcacagaatcgtcgt<br>atgcagtgaaaactctcttcaattctttatgccggtgttgggcgcgttatttatcggagttgcagttgcgcccgcgaacgacatttataatg

	aacgtgaattgctcaacagtatgggcatttcgcagcctaccgtggtgttcgtttccaaaaaggggttgcaaaaaattttgaacgtgcaa<br>aaaaagctcccaatcatccaaaaaattattatcatggattctaaaacggattaccagggatttcagtcgatgtacacgttcgtcacatct<br>catctacctcccggttttaatgaatacgattttgtgccagagtccttcgatagggacaagacaattgcactgatcatgaactcctctggat<br>ctactggtctgcctaaaggtgtcgctctgcctcatagaactgcctgcgtgagattctcgcatgccagagatcctatttttggcaatcaaat<br>cattccggatactgcgattttaagtgttgttccattccatcacggttttggaatgtttactacactcggatatttgatatgtggatttcgagtcgt<br>cttaatgtatagatttgaagaagagctgtttctgaggagccttcaggattacaagattcaaagtgcgctgctggtgccaaccctattctc<br>cttcttcgccaaaagcactctgattgacaaatacgatttatctaatttacacgaaattgcttctggtggcgctcccctctctaaggaagtc<br>ggggaagcggttgccaagaggttccatctgccaggtatcaggcaaggatatgggctcactgagactacatcagctattctgattaca<br>cccgagggggatgataaaccgggcgcggtgagtcatatggggaacttctgttgggtgtttggtggttcgaatcccatcttaagcgtttg<br>tgcgaacggagtctcctgagcttcgctttgtctgtagttttgagctgaaggggagtgtgtgggcgactctcttccttttcagtagatgctgt<br>gccagcctttgcccagaggcgctctagctgggagcaaagtccggtcactgggcagcaccacccccggacttgcatgggtagccg<br>ctgagatggagcctgagcacacgcggccgctgttaacgcagtgtttctcttttttcagcaaccaaacaaccaaacaaccaaacaac<br>caaacaaccaaacaaccaaacaaccaaacaaccaaacaaccaaacaaccaaacaaccacaacacaggttggtgctagctg<br>gccagtgagatccaagaatcttcctgtctctgctgatccactgataggattacaagtacatgccaccaagcccagcttcctcttaccag<br>gtgctggggaccaaacttaggccctcattcctacacagtgaatactttgttaggacttttaatttttggagagcatgtttgccctgtttttttttt<br>ttcttttcttttgctgttgtaataggcccatcatttactttttaattttaagtcttgtgccttgaaactcacctttgttgttggacactttcttttttgaggt<br>cggtaaagttgttccatttttgaagcgaaggttgtggatctggataccgggaaaacgctgggcgttaatcaaagaggcgaactgtgt<br>gtgagaggtcctatgattatgtccggttatgtaaacaatccggaagcgaccaacgccttgattgacaaggatggatggctacattctg<br>gagacatagcttactgggacgaagacgaacacttcttcatcgttgaccgcctgaagtctctgattaagtacaaaggctatcaggtggc<br>tcccgctgaattggaatccatcttgctccaacaccccaacatcttcgacgcaggtgtcgcaggtcttcccgacgatgacgccggtgaa<br>cttcccgccgccgttgttgttttggagcacggaaagacgatgacggaaaaagagatcgtggattacgtcgccagtcaagtaacaac<br>cgcgaaaaagttgcgcggaggagttgtgtttgtggacgaagtaccgaaaggtcttaccggaaaactcgacgcaagaaaaatcag<br>agagatcctcataaaggccaagaagggcggaaagatcgccgtgtaattctagagtcggggcggccggccgcttcgagcagacat<br>gataagatacattgatgagtttggacaaaccacaactagaatgcagtgaaaaaaatgctttatttgtgaaatttgtgatgctattgcttat<br>ttgtaaccattataagctgcaataaacaagttaacaacaacaattgcattcattttatgtttcaggttcagggggaggtgtgggaggtttt<br>taaagcaagtaaaacctctacaaatgtggtaaaactgcagaaggatctgaactcgaggagcttggcccattgcatacgttgtatccat<br>atcataatatgtacatttatattggctcatgtccaacattaccgccatgttgacattgattattgactagttattaatagtaatcaattacggg<br>gtcattagttcatagcccatatatggagttccgcgttacataacttacggtaaatggcccgcctggctgaccgcccaacgacccccgc<br>ccattgacgtcaataatgacgtatgttcccatagtaacgccaatagggactttccattgacgtcaatgggtggagtatttacgctaaact<br>gcccacttggcagtacatcaagtgtatcatatgccaagtacgccccctattgacgtcaatgacggtaaatggcccgcctggcattatg<br>cccagtacatgaccttatgggactttcctacttggcagtacatctacgtattagtcatcgctattaccatggtgatgcggttttggcagtac<br>atcaatgggcgtggatagcggtttgactcacggggatttccaagtctccaccccattgacgtcaatgggagtttgttttggcaccaaaat<br>caacgggactttccaaaatgtcgtaacaactccgccccattgacgcaaatgggcgggatccgtcgaccgatgcccttgagagcctt<br>caacccagtcagctccttccggtgggcgcggggcatgactatcgtcgccgcacttatgactgtcttctttatcatgcaactcgtaggac<br>aggtgccggcagcgctcttccgcttcctcgctcactgactcgctgcgctcggtcgttcggctgcggcgagcggtatcagctcactcaa<br>aggcggtaatacggttatccacagaatcaggggataacgcaggaaagaacatgtgagcaaaaggccagcaaaaggccagga<br>accgtaaaaaggccgcgttgctggcgtttttccataggctccgcccccctgacgagcatcacaaaaatcgacgctcaagtcagagg<br>tggcgaaacccgacaggactataaagataccaggcgtttccccctggaagctccctcgtgcgctctcctgttccgaccctgccgctta<br>ccggatacctgtccgcctttctcccttcgggaagcgtggcgctttctcatagctcacgctgtaggtatctcagttcggtgtaggtcgttcgc<br>tccaagctgggctgtgtgcacgaaccccccgttcagcccgaccgctgcgccttatccggtaactatcgtcttgagtccaacccggtaa<br>gacacgacttatcgccactggcagcagccactggtaacaggattagcagagcgaggtatgtaggcggtgctacagagttcttgaa<br>gtggtggcctaactacggctacactagaagaacagtatttggtatctgcgctctgctgaagccagttaccttcggaaaaagagttggt<br>agctcttgatccggcaaacaaaccaccgctggtagcggtggtttttttgtttgcaagcagcagattacgcgcagaaaaaaaggatctc<br>aagaagatcctttgatcttttctacggggtctgacgctcagtggaacgaaaactcacgttaagggattttggtcatgagattatcaaaaa<br>ggatcttcacctagatccttttaaattaaaaatgaagttttaaatcaatctaaagtatatatgagtaaacttggtctgacagttaccaatgc<br>ttaatcagtgaggcacctatctcagcgatctgtctatttcgttcatccatagttgcctgactccccgtcgtgtagataactacgatacggg<br>agggcttaccatctggccccagtgctgcaatgataccgcgagagccacgctcaccggctccagatttatcagcaataaaccagcc<br>agccggaagggccgagcgcagaagtggtcctgcaactttatccgcctccatccagtctattaattgttgccgggaagctagagtaag<br>tagttcgccagttaatagtttgcgcaacgttgttgccattgctacaggcatcgtggtgtcacgctcgtcgtttggtatggcttcattcagctc<br>cggttcccaacgatcaaggcgagttacatgatcccccatgttgtgcaaaaaagcggttagctccttcggtcctccgatcgttgtcagaa<br>gtaagttggccgcagtgttatcactcatggttatggcagcactgcataattctcttactgtcatgccatccgtaagatgcttttctgtgactg<br>gtgagtactcaaccaagtcattctgagaatagtgtatgcggcgaccgagttgctcttgcccggcgtcaatacgggataataccgcgc<br>cacatagcagaactttaaaagtgctcatcattggaaaacgttcttcggggcgaaaactctcaaggatcttaccgctgttgagatccagt<br>tcgatgtaacccactcgtgcacccaactgatcttcagcatcttttactttcaccagcgtttctgggtgagcaaaaacaggaaggcaaa<br>atgccgcaaaaaagggaataagggcgacacggaaatgttgaatactcatactcttcctttttcaatattattgaagcatttatcagggtt<br>attgtctcatgagcggatacatatttgaatgtatttagaaaaataaacaaataggggttccgcgcacatttccccgaaaagtgccacct<br>gacgcgccctgtagcggcgcattaagcgcggcgggtgtggtggttacgcgcagcgtgaccgctacacttgccagcgccctagcgc<br>ccgctcctttcgctttcttcccttcctttctcgccacgttcgccggctttccccgtcaagctctaaatcgggggctccctttagggttccgattt<br>agtgctttacggcacctcgaccccaaaaaacttgattagggtgatggttcacgtagtgggccatcgccctgatagacggtttttcgccc<br>tttgacgttggagtccacgttctttaatagtggactcttgttccaaactggaacaacactcaaccctatctcggtctattcttttgatttataag<br>ggattttgccgatttcggcctattggttaaaaaatgagctgatttaacaaaaatttaacgcgaattttaacaaaatattaacgcttacaatt<br>t
**pWHE237_empty,** used as positive control, constitutively expressing Firefly luciferase	gccattcgccattcaggctgcgcaactgttgggaagggcgatcggtgcgggcctcttcgctattacgccagcccaagctaccatgat<br>aagtaagtaatattaaggtacgggaggtacttggagcggccctagcccgggctcgagatctgcgatcgtaggcgtgtacggtggga<br>ggtctatataagcagagctcgtttagtgaaccgtcagatcgcctggagacgccatccacgctgttttgacctccatagaagacaccg<br>ggaccgatccagcctccgcggccccgaattcaccatggaagacgccaaaaacataaagaaaggcgtgagtctatgggacccttg<br>atgttttctttccccttcttttctatggttaagttcatgtcataggaaggggagaagtaacagggtacagtttagaatgggaaacagacga<br>atgattgcatcagtgtggaagtctcaggatcgttttagtttcttttatttgctgttcataacaattgttttcttttgtttaattcttgctttcttttttttcttc<br>tccgcaatttttactattatacttaatgccttaacattgtgtataacaaaaggaaatatctctgagatacattaagtaacttaaaaaaaaa<br>ctttacacagtctgcctagtacattactatttggaatatatgtgtgcttatttgcatattcataatctccctactttattttcttttatttttaattgata<br>cataatcattatacatatttatgggttaaagtgtaatgttttaatatgtgtacacatattgaccaaatcagggtaattttgcatttgtaattttaa<br>aaaatgctttcttcttttaatatactttttttgtttatcttatttctaatactttccctaatctctttctttcagggcaataatgatacaatgtatcatgcc<br>tctttgcaccattctaaagaataacagtgataatttctgggttaaggcaatagcaatatttctgcatataaatatttctgcatataaattgta<br>actgatgtaagaggtttcatattgctaatagcagctacaatccagctaccattctgcttttattttatggttgggataaggctggattattctg

	agtccaagctaggcccttttgctaatcatgttcatacctcttatcttcctcccacagccggcgccattctatccgctggaagatggaaccg<br>ctggagagcaactgcataaggctatgaagagatacgccctggttcctggaacaattgcttttacagatgcacatatcgaggtggaca<br>tcacttacgctgagtacttcgaaatgtccgttcggttggcagaagctatgaaacgatatgggctgaatacaaatcacagaatcgtcgt<br>atgcagtgaaaactctcttcaattctttatgccggtgttgggcgcgttatttatcggagttgcagttgcgcccgcgaacgacatttataatg<br>aacgtgaattgctcaacagtatgggcatttcgcagcctaccgtggtgttcgtttccaaaaaggggttgcaaaaaattttgaacgtgcaa<br>aaaaagctcccaatcatccaaaaaattattatcatggattctaaaacggattaccagggatttcagtcgatgtacacgttcgtcacatct<br>catctacctcccggttttaatgaatacgattttgtgccagagtccttcgatagggacaagacaattgcactgatcatgaactcctctggat<br>ctactggtctgcctaaaggtgtcgctctgcctcatagaactgcctgcgtgagattctcgcatgccagagatcctatttttggcaatcaaat<br>cattccggatactgcgattttaagtgttgttccattccatcacggttttggaatgtttactacactcggatatttgatatgtggatttcgagtcgt<br>cttaatgtatagatttgaagaagagctgtttctgaggagccttcaggattacaagattcaaagtgcgctgctggtgccaaccctattctc<br>cttcttcgccaaaagcactctgattgacaaatacgatttatctaatttacacgaaattgcttctggtggcgctcccctctctaaggaagtc<br>ggggaagcggttgccaagaggttccatctgccaggtatcaggcaaggatatgggctcactgagactacatcagctattctgattaca<br>cccgagggggatgataaaccgggcgcggtgagtcatatggggaacttctgttgggtgtttggtggttcgaatcccatcttaagcgttttg<br>tgcgaacggagtctcctgagcttcgctttgtctgtagttttgagctgaaggggggagtgtgtgggcgactctcttccttttcagtagatgttag<br>gacttttaatttttggagagcatgtttgccctgttttttttttttcttttcttttgctgttgtaataggcccatcatttactttttaattttaagtcttgtgcct<br>tgaaactcacctttgttgttggacactttctttttgaggtcggtaaagttgttccatttttttgaagcgaaggttgtggatctggataccgggaa<br>aacgctgggcgttaatcaaagaggcgaactgtgtgtgagaggtcctatgattatgtccggttatgtaaacaatccggaagcgaccaa<br>cgccttgattgacaaggatggatggctacattctggagacatagcttactgggacgaagacgaacacttcttcatcgttgaccgcctg<br>aagtctctgattaagtacaaaggctatcaggtggctcccgctgaattggaatccatcttgctccaacaccccaacatcttcgacgcag<br>gtgtcgcaggtcttcccgacgatgacgccggtgaacttcccgccgccgttgttgttttggagcacggaaagacgatgacggaaaaa<br>gagatcgtggattacgtcgccagtcaagtaacaaccgcgaaaaagttgcgcggaggagttgtgtttgtggacgaagtaccgaaag<br>gtcttaccggaaaactcgacgcaagaaaaatcagagagatcctcataaaggccaagaagggcggaaagatcgccgtgtaattct<br>agagtcggggcggccggccgcttcgagcagacatgataagatacattgatgagtttggacaaaccacaactagaatgcagtgaa<br>aaaaatgctttatttgtgaaatttgtgatgctattgctttatttgtaaccattataagctgcaataaacaagttaacaacaacaattgcattc<br>attttatgtttcaggttcagggggaggtgtgggaggttttttaaagcaagtaaaacctctacaaatgtggtaaactgcagaaggatctga<br>actcgaggagcttggcccattgcatacgttgtatccatatcataatatgtacatttatattggctcatgtccaacattaccgccatgttgac<br>attgattattgactagttattaatagtaatcaattacggggtcattagttcatagcccatatatggagttccgcgttacataacttacggtaa<br>atggcccgcctggctgaccgcccaacgacccccgcccattgacgtcaataatgacgtatgttcccatagtaacgccaatagggactt<br>tccattgacgtcaatgggtggagtatttacgctaaactgcccacttggcagtacatcaagtgtatcatatgccaagtacgccccctattg<br>acgtcaatgacggtaaatggcccgcctggcattatgcccagtacatgaccttatgggactttcctacttggcagtacatctacgtattag<br>tcatcgctattaccatggtgatgcggttttggcagtacatcaatgggcgtggatagcggtttgactcacggggatttccaagtctccacc<br>ccattgacgtcaatgggagtttgttttggcaccaaaatcaacgggactttccaaaatgtcgtaacaactccgccccattgacgcaaat<br>gggcgggatccgtcgaccgatgcccttgagagccttcaacccagtcagctccttccggtgggcgcggggcatgactatcgtcgccg<br>cacttatgactgtcttctttatcatgcaactcgtaggacaggtgccggcagcgctcttccgcttcctcgctcactgactcgctgcgctcggt<br>cgttcggctgcggcgagcggtatcagctcactcaaaggcggtaatacggttatccacagaatcaggggataacgcaggaaagaa<br>catgtgagcaaaaggccagcaaaaggccaggaaccgtaaaaaggccgcgttgctggcgtttttccataggctccgcccccctgac<br>gagcatcacaaaaatcgacgctcaagtcagaggtggcgaaacccgacaggactataaagataccaggcgtttccccctggaag<br>ctccctcgtgcgctctcctgttccgaccctgccgcttaccggatacctgtccgcctttctcccttcgggaagcgtggcgctttctcatagct<br>cacgctgtaggtatctcagttcggtgtaggtcgttcgctccaagctgggctgtgtgcacgaaccccccgttcagcccgaccgctgcgc<br>cttatccggtaactatcgtcttgagtccaacccggtaagacacgacttatcgccactggcagcagccactggtaacaggattagcag<br>agcgaggtatgtaggcggtgctacagagttcttgaagtggtggcctaactacggctacactagaagaacagtatttggtatctgcgct<br>ctgctgaagccagttaccttcggaaaaagagttggtagctcttgatccggcaaacaaaccaccgctggtagcggtggtttttttgtttgc<br>aagcagcagattacgcgcagaaaaaaaggatctcaagaagatcctttgatcttttctacggggtctgacgctcagtggaacgaaaa<br>ctcacgttaagggattttggtcatgagattatcaaaaaggatcttcacctagatccttttaaattaaaaatgaagttttaaatcaatctaaa<br>gtatatatgagtaaacttggtctgacagttaccaatgcttaatcagtgaggcacctatctcagcgatctgtctatttcgttcatccatagttg<br>cctgactccccgtcgtgtagataactacgatacgggagggcttaccatctggccccagtgctgcaatgataccgcgagagccacgc<br>tcaccggctccagatttatcagcaataaaccagccagccggaagggccgagcgcagaagtggtcctgcaactttatccgcctccat<br>ccagtctattaattgttgccgggaagctagagtaagtagttcgccagttaatagtttgcgcaacgttgttgccattgctacaggcatcgtg<br>gtgtcacgctcgtcgtttggtatggcttcattcagctccggttcccaacgatcaaggcgagttacatgatcccccatgttgtgcaaaaaa<br>gcggttagctccttcggtcctccgatcgttgtcagaagtaagttggccgcagtgttatcactcatggttatggcagcactgcataattctct<br>tactgtcatgccatccgtaagatgcttttctgtgactggtgagtactcaaccaagtcattctgagaatagtgtatgcggcgaccgagttgc<br>tcttgcccggcgtcaatacgggataataccgcgccacatagcagaactttaaaagtgctcatcattggaaaacgttcttcggggcga<br>aaactctcaaggatcttaccgctgttgagatccagttcgatgtaacccactcgtgcacccaactgatcttcagcatcttttactttcacca<br>gcgtttctgggtgagcaaaaacaggaaggcaaaatgccgcaaaaaagggaataagggcgacacggaaatgttgaatactcata<br>ctcttcctttttcaatattattgaagcatttatcagggttattgtctcatgagcggatacatatttgaatgtatttagaaaaataaacaaatag<br>gggttccgcgcacatttccccgaaaagtgccacctgacgcgccctgtagcggcgcattaagcgcggcgggtgtggtggttacgcgc<br>agcgtgaccgctacacttgccagcgccctagcgcccgctcctttcgctttcttcccttcctttctcgccacgttcgccggctttccccgtca<br>agctctaaatcgggggctccctttagggttccgatttagtgctttacggcacctcgaccccaaaaaacttgattagggtgatggttcacg<br>tagtgggccatcgccctgatagacggtttttcgccctttgacgttggagtccacgttctttaatagtggactcttgttccaaactggaacaa<br>cactcaaccctatctcggtctattcttttgatttataagggattttgccgatttcggcctattggttaaaaaatgagctgatttaacaaaaattt<br>aacgcgaatttaacaaaatattaacgcttacaattt
**pWHE237_TC,** containing a splice-regualting construct under control of the tetracycline aptamer, enabling tetracycline-dependent Firefly expression	gccattcgccattcaggctgcgcaactgttgggaagggcgatcggtgcgggcctcttcgctattacgccagcccaagctaccatgat<br>aagtaagtaatattaaggtacgggaggtacttggagcggccctagcccgggctcgagatctgcgatcgtaggcgtgtacggtggga<br>ggtctatataagcagagctcgtttagtgaaccgtcagatcgcctggagacgccatccacgctgttttgacctccatagaagacaccg<br>ggaccgatccagcctccgcggccccgaattcaccatggaagacgccaaaaacataaagaaaggcgtgagtctatgggacccttg<br>atgttttctttccccttcttttctatggttaagttcatgtcataggaaggggagaagtaacagggtacagtttagaatgggaaacagacga<br>atgattgcatcagtgtggaagtctcaggatcgttttagtttcttttatttgctgttcataacaattgttttcttttgtttaattcttgctttctttttttttcttc<br>tccgcaatttttactattatacttaatgccttaacattgtgtataacaaaaggaaatatctctgagatacattaagtaacttaaaaaaaaa<br>ctttacacagtctgcctagtacattactatttggaatatatgtgtgcttatttgcatattcataatctccctactttattttcttttatttttaattgata<br>cataatcattatacatatttatgggttaaagtgtaatgttttaatatgtgtacacatattgaccaaatcagggtaattttgcatttgtaattttaa<br>aaaatgctttcttcttttaatatactttttgtttatcttatttctaatactttccctaatctctttctttcagggcaataatgatacaatgtatcatgcc<br>tctttgcaccattctaaagaataacagtgataatttctgggttaaggcaatagcaatatttctgcatataaatatttctgcatataaattgta<br>actgatgtaagaggtttcatattgctaatagcagctacaatccagctaccattctgcttttattttatggttgggataaggctggattattctg<br>agtccaagctaggcccttttgctaatcatgttcatacctcttatcttcctcccacagccggcgccattctatccgctggaagatggaaccg

	ctggagagcaactgcataaggctatgaagagatacgccctggttcctggaacaattgcttttacagatgcacatatcgaggtggaca<br>tcacttacgctgagtacttcgaaatgtccgttcggttggcagaagctatgaaacgatatgggctgaatacaaatcacagaatcgtcgt<br>atgcagtgaaaactctcttcaattctttatgccggtgttgggcgcgttatttatcggagttgcagttgcgcccgcgaacgacatttataatg<br>aacgtgaattgctcaacagtatgggcatttcgcagcctaccgtggtgttcgtttccaaaaaggggttgcaaaaaattttgaacgtgcaa<br>aaaaagctcccaatcatccaaaaaattattatcatggattctaaaacggattaccagggatttcagtcgatgtacacgttcgtcacatct<br>catctacctcccggttttaatgaatacgattttgtgccagagtccttcgatagggacaagacaattgcactgatcatgaactcctctggat<br>ctactggtctgcctaaaggtgtcgctctgcctcatagaactgcctgcgtgagattctcgcatgccagagatcctatttttggcaatcaaat<br>cattccggatactgcgattttaagtgttgttccattccatcacggttttggaatgtttactacactcggatatttgatatgtggatttcgagtcgt<br>cttaatgtatagatttgaagaagagctgtttctgaggagccttcaggattacaagattcaaagtgcgctgctggtgccaaccctattctc<br>cttcttcgccaaaagcactctgattgacaaatacgatttatctaatttacacgaaattgcttctggtggcgctcccctctctaaggaagtc<br>ggggaagcggttgccaagaggttccatctgccaggtatcaggcaaggatatgggctcactgagactacatcagctattctgattaca<br>cccgagggggatgataaaccgggcgcggtgagtcatatggggaacttctgttgggtgtttggtggttcgaatcccatcttaagcgttttg<br>tgcgaacggagtctcctgagcttcgctttgtctgtagttttgagctgaagggggagtgtgtgggcgactctcttccttttcagtagatgctgt<br>gccagcctttgcccagaggcgctctagctgggagcaaagtccggtcactgggcagcaccaccccccggacttgcatgggtagccg<br>ctgagatggagcctgagcacacgcggccgctgttaacgcagtgtttctctttttttcagcgcgcgcaaaacataccagatgaaagtct<br>ggagaggtgaagaatacgaccacctgcgcgcgctgaaacaaccaaacaaccaaacaaccaaacaaccaaacaaccaaaca<br>accaaacaaccaaacaaccaaacaaccaaacaaccaaacaaccacaacacaggttggtgctagctggccagtgagatccaa<br>gaatcttcctgtctctgctgatccactgataggattacaagtacatgccaccaagcccagcttcctcttaccaggtgctggggaccaaa<br>cttaggccctcattcctacacagtgaatactttgttaggacttttaatttttggagagcatgtttgccctgtttttttttttctttcttttgctgttgta<br>ataggcccatcatttactttttaattttaagtcttgtgccttgaaactcacctttgttgttggacactttcttttttgaggtcggtaaagttgttccatt<br>ttttgaagcgaaggttgtggatctggataccgggaaaacgctgggcgttaatcaaagaggcgaactgtgtgtgagaggtcctatgatt<br>atgtccggttatgtaaacaatccggaagcgaccaacgccttgattgacaaggatggatggctacattctggagacatagcttactgg<br>gacgaagacgaacacttcttcatcgttgaccgcctgaagtctctgattaagtacaaaggctatcaggtggctcccgctgaattggaat<br>ccatcttgctccaacaccccaacatcttcgacgcaggtgtcgcaggtcttcccgacgatgacgccggtgaacttcccgccgccgttgt<br>tgttttggagcacggaaagacgatgacggaaaaagagatcgtggattacgtcgccagtcaagtaacaaccgcgaaaaagttgcg<br>cggaggagttgtgtttgtggacgaagtaccgaaaggtcttaccggaaaactcgacgcaagaaaaatcagagagatcctcataaag<br>gccaagaagggcggaaagatcgccgtgtaattctagagtcggggcggccggccgcttcgagcagacatgataagatacattgat<br>gagtttggacaaaccacaactagaatgcagtgaaaaaaatgctttatttgtgaaatttgtgatgctattgctttatttgtaaccattataag<br>ctgcaataaacaagttaacaacaacaattgcattcattttatgtttcaggttcagggggaggtgtgggaggttttttaaagcaagtaaaa<br>cctctacaaatgtggtaaactgcagaaggatctgaactcgaggagcttggcccattgcatacgttgtatccatatcataatatgtacatt<br>tatattggctcatgtccaacattaccgccatgttgacattgattattgactagttattaatagtaatcaattacggggtcattagttcatagcc<br>catatatggagttccgcgttacataacttacggtaaatggcccgcctggctgaccgcccaacgacccccgcccattgacgtcaataa<br>tgacgtatgttcccatagtaacgccaatagggactttccattgacgtcaatgggtggagtatttacgctaaactgcccacttggcagtac<br>atcaagtgtatcatatgccaagtacgccccctattgacgtcaatgacggtaaatggcccgcctggcattatgcccagtacatgacctta<br>tgggactttcctacttggcagtacatctacgtattagtcatcgctattaccatggtgatgcggttttggcagtacatcaatgggcgtggata<br>gcggtttgactcacggggatttccaagtctccaccccattgacgtcaatgggagtttgttttggcaccaaaatcaacgggactttccaa<br>aatgtcgtaacaactccgccccattgacgcaaatgggcgggatccgtcgaccgatgcccttgagagccttcaacccagtcagctcct<br>tccggtgggcgcggggcatgactatcgtcgccgcacttatgactgtcttctttatcatgcaactcgtaggacaggtgccggcagcgctc<br>ttccgcttcctcgctcactgactcgctgcgctcggtcgttcggctgcggcgagcggtatcagctcactcaaaggcggtaatacggttat<br>ccacagaatcaggggataacgcaggaaagaacatgtgagcaaaaggccagcaaaaggccaggaaccgtaaaaaggccgc<br>gttgctggcgtttttccataggctccgcccccctgacgagcatcacaaaaatcgacgctcaagtcagaggtggcgaaacccgacag<br>gactataaagataccaggcgtttccccctggaagctccctcgtgcgctctcctgttccgaccctgccgcttaccggatacctgtccgcct<br>ttctcccttcgggaagcgtggcgctttctcatagctcacgctgtaggtatctcagttcggtgtaggtcgttcgctccaagctgggctgtgtg<br>cacgaaccccccgttcagcccgaccgctgcgccttatccggtaactatcgtcttgagtccaacccggtaagacacgacttatcgcca<br>ctggcagcagccactggtaacaggattagcagagcgaggtatgtaggcggtgctacagagttcttgaagtggtggcctaactacgg<br>ctacactagaagaacagtatttggtatctgcgctctgctgaagccagttaccttcggaaaaagagttggtagctcttgatccggcaaac<br>aaaccaccgctggtagcggtggtttttttgtttgcaagcagcagattacgcgcagaaaaaaaggatctcaagaagatcctttgatctttt<br>ctacggggtctgacgctcagtggaacgaaaactcacgttaagggattttggtcatgagattatcaaaaaggatcttcacctagatcctt<br>ttaaattaaaaatgaagttttaaatcaatctaaagtatatatgagtaaacttggtctgacagttaccaatgcttaatcagtgaggcaccta<br>tctcagcgatctgtctatttcgttcatccatagttgcctgactccccgtcgtgtagataactacgatacgggagggcttaccatctggccc<br>cagtgctgcaatgataccgcgagagccacgctcaccggctccagatttatcagcaataaaccagccagccggaagggccgagc<br>gcagaagtggtcctgcaactttatccgcctccatccagtctattaattgttgccgggaagctagagtaagtagttcgccagttaatagttt<br>gcgcaacgttgttgccattgctacaggcatcgtggtgtcacgctcgtcgtttggtatggcttcattcagctccggttcccaacgatcaagg<br>cgagttacatgatcccccatgttgtgcaaaaaagcggttagctccttcggtcctccgatcgttgtcagaagtaagttggccgcagtgtta<br>tcactcatggttatggcagcactgcataattctcttactgtcatgccatccgtaagatgcttttctgtgactggtgagtactcaaccaagtc<br>attctgagaatagtgtatgcggcgaccgagttgctcttgcccggcgtcaatacgggataataccgcgccacatagcagaactttaaa<br>agtgctcatcattggaaaacgttcttcggggcgaaaactctcaaggatcttaccgctgttgagatccagttcgatgtaacccactcgtg<br>cacccaactgatcttcagcatcttttactttcaccagcgtttctgggtgagcaaaaacaggaaggcaaaatgccgcaaaaaaggga<br>ataagggcgacacggaaatgttgaatactcatactcttcctttttcaatattattgaagcatttatcagggttattgtctcatgagcggatac<br>atatttgaatgtatttagaaaaataaacaaataggggttccgcgcacatttccccgaaaagtgccacctgacgcgccctgtagcggc<br>gcattaagcgcggcgggtgtggtggttacgcgcagcgtgaccgctacacttgccagcgccctagcgcccgctcctttcgctttcttccc<br>ttcctttctcgccacgttcgccggctttccccgtcaagctctaaatcgggggctccctttagggttccgatttagtgctttacggcacctcga<br>ccccaaaaaacttgattagggtgatggttcacgtagtgggccatcgccctgatagacggtttttcgccctttgacgttggagtccacgtt<br>ctttaatagtggactcttgttccaaactggaacaacactcaaccctatctcggtctattcttttgatttataagggattttgccgatttcggcct<br>attggttaaaaaatgagctgatttaacaaaaatttaacgcgaattttaacaaaatattaacgcttacaattt

**pWHE237_Dox-G12,**<br>containing a splice-regualting construct under control of the doxycycline aptamer G12, enabling doxycycline-dependent Firefly expression	gccattcgccattcaggctgcgcaactgttgggaagggcgatcggtgcgggcctcttcgctattacgccagcccaagctaccatgat<br>aagtaagtaatattaaggtacgggaggtacttggagcggccctagcccgggctcgagatctgcgatcgtaggcgtgtacggtggga<br>ggtctatataagcagagctcgtttagtgaaccgtcagatcgcctggagacgccatccacgctgttttgacctccatagaagacaccg<br>ggaccgatccagcctccgcggccccgaattcaccatggaagacgccaaaaacataaagaaaggcgtgagtctatgggacccttg<br>atgttttctttccccttcttttctatggttaagttcatgtcataggaaggggagaagtaacagggtacagtttagaatgggaaacagacga<br>atgattgcatcagtgtggaagtctcaggatcgttttagtttcttttatttgctgttcataacaattgttttctttgtttaattcttgctttctttttttttcttc<br>tccgcaatttttactattatacttaatgccttaacattgtgtataacaaaaggaaatatctctgagatacattaagtaacttaaaaaaaaa<br>ctttacacagtctgcctagtacattactatttggaatatatgtgtgcttatttgcatattcataatctccctactttattttcttttattttttaattgata<br>cataatcattatacatatttatgggttaaagtgtaatgttttaatatgtgtacacatattgaccaaatcagggtaattttgcatttgtaattttaa<br>aaaatgctttcttcttttaatatacttttttgtttatcttatttctaatactttccctaatctctttctttcagggcaataatgatacaatgtatcatgcc<br>tctttgcaccattctaaagaataacagtgataatttctgggttaaggcaatagcaatatttctgcatataaatatttctgcatataaattgta<br>actgatgtaagaggtttcatattgctaatagcagctacaatccagctaccattctgcttttattttatggttgggataaggctggattattctg<br>agtccaagctaggcccttttgctaatcatgttcatacctcttatcttcctcccacagccggcgccattctatccgctggaagatggaaccg<br>ctggagagcaactgcataaggctatgaagagatacgccctggttcctggaacaattgcttttacagatgcacatatcgaggtggaca<br>tcacttacgctgagtacttcgaaatgtccgttcggttggcagaagctatgaaacgatatgggctgaatacaaatcacagaatcgtcgt<br>atgcagtgaaaactctcttcaattctttatgccggtgttgggcgcgttatttatcggagttgcagttgcgcccgcgaacgacatttataatg<br>aacgtgaattgctcaacagtatgggcatttcgcagcctaccgtggtgttcgtttccaaaaaggggttgcaaaaaattttgaacgtgcaa<br>aaaaagctcccaatcatccaaaaaattattatcatggattctaaaacggattaccagggatttcagtcgatgtacacgttcgtcacatct<br>catctacctcccggttttaatgaatacgattttgtgccagagtccttcgatagggacaagacaattgcactgatcatgaactcctctggat<br>ctactggtctgcctaaaggtgtcgctctgcctcatagaactgcctgcgtgagattctcgcatgccagagatcctatttttggcaatcaaat<br>cattccggatactgcgattttaagtgttgttccattccatcacggttttggaatgtttactacactcggatatttgatatgtggatttcgagtcgt<br>cttaatgtatagatttgaagaagagctgtttctgaggagccttcaggattacaagattcaaagtgcgctgctggtgccaaccctattctc<br>cttcttcgccaaaagcactctgattgacaaatacgatttatctaatttacacgaaattgcttctggtggcgctcccctctctaaggaagtc<br>ggggaagcggttgccaagaggttccatctgccaggtatcaggcaaggatatgggctcactgagactacatcagctattctgattaca<br>cccgagggggatgataaaccgggcgcggtgagtcatatggggaacttctgttgggtgtttggtggttcgaatcccatcttaagcgtttg<br>tgcgaacggagtctcctgagcttcgcttttgtctgtagttttgagctgaagggggagtgtgtgggcgactctcttccttttcagtagatgctgt<br>gccagcctttgcccagaggcgctctagctgggagcaaagtccggtcactgggcagcaccacccccggacttgcatgggtagccg<br>ctgagatggagcctgagcacacgcggccgctgttaacgcagtgtttctcttttttcagcgcgcgcggtcattgagtgaacagacgtca<br>agctagttgggctactggcttcgcgcgcgctgaaacaaccaaacaaccaaacaaccaaacaaccaaacaaccaaacaaccaa<br>acaaccaaacaaccaaacaaccaaacaaccaaacaaccacaacacaggttggtgctagctggccagtgagatccaagaatctt<br>cctgtctctgctgatccactgataggattacaagtacatgccaccaagcccagcttcctcttaccaggtgctggggaccaaacttagg<br>ccctcattcctacacagtgaatactttgttaggacttttaatttttggagagcatgtttgccctgttttttttttttcttttcttttgctgttgtaatagg<br>cccatcatttactttttaattttaagtcttgtgccttgaaactcacctttgttgttggacactttcttttttgaggtcggtaaagttgttccatttttga<br>agcgaaggttgtggatctggataccgggaaaacgctgggcgttaatcaaagaggcgaactgtgtgtgagaggtcctatgattatgtc<br>cggttatgtaaacaatccggaagcgaccaacgccttgattgacaaggatggatggctacattctggagacatagcttactgggacg<br>aagacgaacacttcttcatcgttgaccgcctgaagtctctgattaagtacaaaggctatcaggtggctcccgctgaattggaatccatc<br>ttgctccaacaccccaacatcttcgacgcaggtgtcgcaggtcttcccgacgatgacgccggtgaacttcccgccgccgttgttgttttg<br>gagcacggaaagacgatgacggaaaaagagatcgtggattacgtcgccagtcaagtaacaaccgcgaaaaagttgcgcggag<br>gagttgtgtttgtggacgaagtaccgaaaggtcttaccggaaaactcgacgcaagaaaaatcagagagatcctcataaaggccaa<br>gaagggcggaaagatcgccgtgtaattctagagtcggggcggccggccgcttcgagcagacatgataagatacattgatgagtttg<br>gacaaaccacaactagaatgcagtgaaaaaaatgctttatttgtgaaatttgtgatgctattgctttatttgtaaccattataagctgcaat<br>aaacaagttaacaacaacaattgcattcattttatgtttcaggttcagggggaggtgtgggaggttttttaaagcaagtaaaacctctac<br>aaatgtggtaaactgcagaaggatctgaactcgaggagcttggcccattgcatacgttgtatccatatcataatatgtacatttatattgg<br>ctcatgtccaacattaccgccatgttgacattgattattgactagttattaatagtaatcaattacggggtcattagttcatagcccatatat<br>ggagttccgcgttacataacttacggtaaatggcccgcctggctgaccgcccaacgacccccgcccattgacgtcaataatgacgt<br>atgttcccatagtaacgccaatagggactttccattgacgtcaatgggtggagtatttacgctaaactgcccacttggcagtacatcaa<br>gtgtatcatatgccaagtacgccccctattgacgtcaatgacggtaaatggcccgcctggcattatgcccagtacatgaccttatggg<br>actttcctacttggcagtacatctacgtattagtcatcgctattaccatggtgatgcggttttggcagtacatcaatgggcgtggatagcg<br>gtttgactcacggggatttccaagtctccaccccattgacgtcaatgggagtttgttttggcaccaaaatcaacgggactttccaaaatg<br>tcgtaacaactccgccccattgacgcaaatgggcgggatccgtcgaccgatgcccttgagagccttcaacccagtcagctccttccg<br>gtgggcgcggggcatgactatcgtcgccgcacttatgactgtcttctttatcatgcaactcgtaggacaggtgccggcagcgctcttcc<br>gcttcctcgctcactgactcgctgcgctcggtcgttcggctgcggcgagcggtatcagctcactcaaaggcggtaatacggttatcca<br>cagaatcaggggataacgcaggaaagaacatgtgagcaaaaggccagcaaaaggccaggaaccgtaaaaaggccgcgttg<br>ctggcgtttttccataggctccgcccccctgacgagcatcacaaaaatcgacgctcaagtcagaggtggcgaaacccgacaggac<br>tataaagataccaggcgtttccccctggaagctccctcgtgcgctctcctgttccgaccctgccgcttaccggatacctgtccgcctttct<br>cccttcgggaagcgtggcgctttctcatagctcacgctgtaggtatctcagttcggtgtaggtcgttcgctccaagctgggctgtgtgcac<br>gaaccccccgttcagcccgaccgctgcgccttatccggtaactatcgtcttgagtccaacccggtaagacacgacttatcgccactg<br>gcagcagccactggtaacaggattagcagagcgaggtatgtaggcggtgctacagagttcttgaagtggtggcctaactacggcta<br>cactagaagaacagtatttggtatctgcgctctgctgaagccagttaccttcggaaaaagagttggtagctcttgatccggcaaacaa<br>accaccgctggtagcggtggtttttttgtttgcaagcagcagattacgcgcagaaaaaaaggatctcaagaagatcctttgatcttttct<br>acggggtctgacgctcagtggaacgaaaactcacgttaagggattttggtcatgagattatcaaaaaggatcttcacctagatcctttt<br>aaattaaaaatgaagttttaaatcaatctaaagtatatatgagtaaacttggtctgacagttaccaatgcttaatcagtgaggcacctat<br>ctcagcgatctgtctatttcgttcatccatagttgcctgactccccgtcgtgtagataactacgatacgggagggcttaccatctggcccc<br>agtgctgcaatgataccgcgagagccacgctcaccggctccagatttatcagcaataaaccagccagccggaagggccgagcg<br>cagaagtggtcctgcaactttatccgcctccatccagtctattaattgttgccgggaagctagagtaagtagttcgccagttaatagtttg<br>cgcaacgttgttgccattgctacaggcatcgtggtgtcacgctcgtcgtttggtatggcttcattcagctccggttcccaacgatcaaggc<br>gagttacatgatcccccatgttgtgcaaaaaagcggttagctccttcggtcctccgatcgttgtcagaagtaagttggccgcagtgttat<br>cactcatggttatggcagcactgcataattctcttactgtcatgccatccgtaagatgcttttctgtgactggtgagtactcaaccaagtca<br>ttctgagaatagtgtatgcggcgaccgagttgctcttgcccggcgtcaatacgggataataccgcgccacatagcagaactttaaaa<br>gtgctcatcattggaaaacgttcttcggggcgaaaactctcaaggatcttaccgctgttgagatccagttcgatgtaacccactcgtgc<br>acccaactgatcttcagcatcttttactttcaccagcgtttctgggtgagcaaaaacaggaaggcaaaatgccgcaaaaaagggaat<br>aagggcgacacggaaatgttgaatactcatactcttcctttttcaatattattgaagcatttatcagggttattgtctcatgagcggatacat<br>atttgaatgtatttagaaaaataaacaaataggggttccgcgcacatttccccgaaaagtgccacctgacgcgccctgtagcggcgc<br>attaagcgcggcgggtgtggtggttacgcgcagcgtgaccgctacacttgccagcgccctagcgcccgctcctttcgctttcttcccttc

	ctttctcgccacgttcgccggctttccccgtcaagctctaaatcgggggctccctttagggttccgatttagtgctttacggcacctcgacc<br>ccaaaaaacttgattagggtgatggttcacgtagtgggccatcgccctgatagacggtttttcgccctttgacgttggagtccacgttcttt<br>aatagtggactcttgttccaaactggaacaacactcaaccctatctcggtctattcttttgatttataagggattttgccgatttcggcctatt<br>ggttaaaaaatgagctgatttaacaaaaatttaacgcgaattttaacaaaatattaacgcttacaattt
**pJO_mCherry**, plasmid used as transfection control in human cells constitutively expressing mCherry	gtagtccttagacttgggaccaatatgaccgccatgttggcattgattattgactagttattaatagtaatcaattacggggtcattagttca<br>tagcccatatatggagttccgcgttacataacttacggtaaatggcccgcctggctgaccgcccaacgacccccgcccattgacgtc<br>aataatgacgtatgttcccatagtaacgccaatagggactttccattgacgtcaatgggtggagtatttacggtaaactgcccacttgg<br>cagtacatcaagtgtatcatatgccaagtccgccccctattgacgtcaatgacggtaaatggcccgcctggcattatgcccagtacat<br>gaccttacgggactttcctacttggcagtacatctacgtattagtcatcgctattaccatggtgatgcggttttggcagtacaccaatggg<br>cgtggatagcggtttgactcacggggatttccaagtctccaccccattgacgtcaatgggagtttgttttggcaccaaaatcaacggga<br>ctttccaaaatgtcgtaataaccccgccccgttgacgcaaatgggcggtaggcgtgtacggtgggaggtctatataagcagagctcg<br>tttagtgaaccgtcagatcactagaagctttattgcggtagtttatcacagttaaattgctaacgcagtcagtgggcctcggcggccaa<br>gcttggcaatccggtactgttggtaaagccaccacgcgtccagtgtggtggaattcatggtgagcaagggcgaggaggataacatg<br>gccatcatcaaggagttcatgcgcttcaaggtgcacatggagggctccgtgaacggccacgagttcgagatcgagggcgagggc<br>gagggccgcccctacgagggcacccagaccgccaagctgaaggtgaccaagggtggccccctgcccttcgcctgggacatcct<br>gtcccctcagttcatgtacggctccaaggcctacgtgaagcaccccgccgacatccccgactacttgaagctgtccttccccgaggg<br>cttcaagtgggagcgcgtgatgaacttcgaggacggcggcgtggtgaccgtgacccaggactcctccctgcaggacggcgagttc<br>atctacaaggtgaagctgcgcggcaccaacttcccctccgacggccccgtaatgcagaagaagaccatgggctgggaggcctcc<br>tccgagcggatgtaccccgaggacggcgccctgaagggcgagatcaagcagaggctgaagctgaaggacggcggccactac<br>gacgctgaggtcaagaccacctacaaggccaagaagcccgtgcagctgcccggcgcctacaacgtcaacatcaagttggacat<br>cacctcccacaacgaggactacaccatcgtggaacagtacgaacgcgccgagggccgccactccaccggcggcatggacgag<br>ctgtacaagtaatctagatgaatgacaacaacacccggggtcggggcggccggccgcttcgagcagacatgataagatacattga<br>tgagtttggacaaaccacaactagaatgcagtgaaaaaaatgctttatttgtgaaatttgtgatgctattgctttatttgtaaccattataag<br>ctgcaataaacaagttaacaacaacaattgcattcattttatgtttcaggttcagggggaggtgtgggaggttttttaaagcaagtaaaa<br>cctctacaaatgtggtaaaatcgataaggatccgtcgaccgatgcccttgagagccttcaacccagtcagctccttccggtgggcgc<br>ggggcatgactatcgtcgccgcacttatgactgtcttctttatcatgcaactcgtaggacaggtgccggcagcgctcttccgcttcctcg<br>ctcactgactcgctgcgctcggtcgttcggctgcggcgagcggtatcagctcactcaaaggcggtaatacggttatccacagaatca<br>ggggataacgcaggaaagaacatgtgagcaaaaggccagcaaaaggccaggaaccgtaaaaaggccgcgttgctggcgtttt<br>ccataggctccgcccccctgacgagcatcacaaaaatcgacgctcaagtcagaggtggcgaaacccgacaggactataaagat<br>accaggcgtttccccctggaagctccctcgtgcgctctcctgttccgaccctgccgcttaccggatacctgtccgcctttctcccttcggg<br>aagcgtggcgctttctcatagctcacgctgtaggtatctcagttcggtgtaggtcgttcgctccaagctgggctgtgtgcacgaacccc<br>cgttcagcccgaccgctgcgccttatccggtaactatcgtcttgagtccaacccggtaagacacgacttatcgccactggcagcagc<br>cactggtaacaggattagcagagcgaggtatgtaggcggtgctacagagttcttgaagtggtggcctaactacggctacactagaa<br>gaacagtatttggtatctgcgctctgctgaagccagttaccttcggaaaaagagttggtagctcttgatccggcaaacaaaccaccgc<br>tggtagcggtggtttttttgtttgcaagcagcagattacgcgcagaaaaaaaggatctcaagaagatcctttgatcttttctacggggtct<br>gacgctcagtggaacgaaaactcacgttaagggattttggtcatgagattatcaaaaaggatcttcacctagatccttttaaattaaaa<br>atgaagttttaaatcaatctaaagtatatatgagtaaacttggtctgacagcggccgcaaatgctaaaccactgcagtggttaccagtg<br>cttgatcagtgaggcaccgatctcagcgatctgcctatttcgttcgtccatagtggcctgactccccgtcgtgtagatcactacgattcgt<br>gagggcttaccatcaggccccagcgcagcaatgatgccgcgagagccgcgttcaccggcccccgatttgtcagcaatgaaccag<br>ccagcagggagggccgagcgaagaagtggtcctgctactttgtccgcctccatccagtctatgagctgctgtcgtgatgctagagta<br>agaagttcgccagtgagtagtttccgaagagttgtggccattgctactggcatcgtggtatcacgctcgtcgttcggtatggcttcgttca<br>actctggttcccagcggtcaagccgggtcacatgatcacccatattatgaagaaatgcagtcagctccttagggcctccgatcgttgtc<br>agaagtaagttggccgcggtgttgtcgctcatggtaatggcagcactacacaattctcttaccgtcatgccatccgtaagatgcttttcc<br>gtgaccggcgagtactcaaccaagtcgttttgtgagtagtgtatacggcgaccaagctgctcttgcccggcgtctatacgggacaac<br>accgcgccacatagcagtactttgaaagtgctcatcatcgggaatcgttcttcggggcggaaagactcaaggatcttgccgctattga<br>gatccagttcgatatagcccactcttgcacccagttgatcttcagcatcttttactttcaccagcgtttcggggtgtgcaaaaacaggcaa<br>gcaaaatgccgcaaagaagggaatgagtgcgacacgaaaatgttggatgctcatactcgtcctttttcaatattattgaagcatttatc<br>agggttac
**pJO_splice-GG**, empty plasmid for the insertion of aptamers controlling mRNA splicing, aptamer sequences can be inserted through Golden Gate assembly using BsaI	attggccattagccatattattcattggttatatagcataaatcaatattggctattggccattgcatacgttgtatctatatcataatatgtac<br>atttatattggctcatgtccaatatgaccgccatgttggcattgattattgactagttattaatagtaatcaattacggggtcattagttcata<br>gcccatatatggagttccgcgttacataacttacggtaaatggcccgcctggctgaccgcccaacgacccccgcccattgacgtcaa<br>taatgacgtatgttcccatagtaacgccaatagggactttccattgacgtcaatgggtggagtatttacggtaaactgcccacttggca<br>gtacatcaagtgtatcatatgccaagtccgccccctattgacgtcaatgacggtaaatggcccgcctggcattatgcccagtacatga<br>ccttacgggactttcctacttggcagtacatctacgtattagtcatcgctattaccatggtgatgcggttttggcagtacaccaatgggcgt<br>ggatagcggtttgactcacggggatttccaagtctccaccccattgacgtcaatgggagtttgttttggcaccaaaatcaacgggacttt<br>ccaaaatgtcgtaataaccccgccccgttgacgcaaatgggcggtaggcgtgtacggtgggaggtctatataagcagagctcgttta<br>gtgaaccgtcagatcactagaagctttattgcggtagtttatcacagttaaattgctaacgcagtcagtgggcctcggcggccaagctt<br>ggcaatccggtactgttggtaaagccacctcgagctagcaccggtatggtgagcaagggcgaggagctgttcaccggggtggtgc<br>ccatcctggtcgagcgtgagtctatgggacccttgatgttttctttccccttcttttctatggttaagttcatgtcataggaaggggagaagt<br>aacagggtacagtttagaatgggaaacagacgaatgattgcatcagtgtggaagtctcaggatcgttttagtttcttttatttgctgttcat<br>aacaattgttttcttttgtttaattcttgctttctttttttttcttctccgcaatttttactattatacttaatgccttaacattgtgtataacaaaaggaa<br>atatctctgagatacattaagtaacttaaaaaaaaactttacacagtctgcctagtacattactatttggaatatatgtgtgcttatttgcat<br>attcataatctccctactttattttcttttatttttaattgatacataatcattatacattgctgtgccagcctttgcccagaggcgctctagctgg<br>gagcaaagtccggtcactgggcagcaccacccccccggacttgcatgggtagccgctgagatggagcctgagcacacgcggccg<br>ctgttaacgcagtgtttctcttttttttcaggAgACCaaacataccagatgaaagtctggagaggtgaagaatacgaccacGGTCT<br>Ccctgaaacaaccaaacaaccaaacaaccaaacaaccaaacaaccaaacaaccaaacaaccaaacaaccaaacaacca<br>aacaaccaaacaaccaaacaacacaggttggtgctagctggccagtgagatccaagaatcttcctgtctctgctgatccactgata<br>ggattacaagtacatgccaccaagcccagcttcctcttaccaggtgctggggaccaaacttaggccctcattcctacacagtgaata<br>cttatttatgggttaaagtgtaatgttttaatatgtgtacacatattgaccaaatcagggtaattttgcatttgtaattttaaaaaatgctttcttct<br>tttaatatactttttttgtttatcttatttctaatactttccctaatctctttctttcagggcaataatgatacaatgtatcatgcctctttgcaccattct<br>aaagaataacagtgataatttctgggttaaggcaatagcaatatttctgcatataaatatttctgcatataaattgtaactgatgtaagag<br>gtttcatattgctaatagcagctacaatccagctaccattctgcttttattttatggttgggataaggctggattattctgagtccaagctagg<br>cccttttgctaatcatgttcatacctcttatcttcctcccacagtggacggcgacgtaaacggccacaagttcagcgtgtccggcgagg<br>gcgagggcgatgccacctacggcaagctgaccctgaagttcatctgcaccaccggcaagctgcccgtgccctggcccaccctcgt

	gaccaccctgacctacggcgtgcagtgcttcagccgctaccccgaccacatgaagcagcacgacttcttcaagtccgccatgcccg<br>aaggctacgtccaggagcgcaccatcttcttcaaggacgacggcaactacaagacccgcgccgaggtgaagttcgagggcgac<br>accctggtgaaccgcatcgagctgaagggcatcgacttcaaggaggacggcaacatcctggggcacaagctggagtacaacta<br>caacagccacaacgtctatatcatggccgacaagcagaagaacggcatcaaggtgaacttcaagatccgccacaacatcgagg<br>acggcagcgtgcagctcgccgaccactaccagcagaacacccccatcggcgacggccccgtgctgctgcccgacaaccactac<br>ctgagcacccagtccgccctgagcaaagaccccaacgagaagcgcgatcacatggtcctgctggagttcgtgaccgccgccggg<br>atcactctcggcatggacgagctgtacaagcccaaaaaaaaacgaaaagtagaggatccctgatctagagtcggggcggccgg<br>ccgcttcgagcagacatgataagatacattgatgagtttggacaaaccacaactagaatgcagtgaaaaaaatgctttatttgtgaa<br>atttgtgatgctattgctttatttgtaaccattataagctgcaataaacaagttaacaacaacaattgcattcattttatgtttcaggttcagg<br>gggaggtgtgggaggttttttaaagcaagtaaaacctctacaaatgtggtaaaatcgataaggatccgtcgaccgatgcccttgaga<br>gccttcaacccagtcagctccttccggtgggcgcggggcatgactatcgtcgccgcacttatgactgtcttctttatcatgcaactcgta<br>ggacaggtgccggcagcgctcttccgcttcctcgctcactgactcgctgcgctcggtcgttcggctgcggcgagcggtatcagctcac<br>tcaaaggcggtaatacggttatccacagaatcaggggataacgcaggaaagaacatgtgagcaaaaggccagcaaaaggcca<br>ggaaccgtaaaaaggccgcgttgctggcgtttttccataggctccgcccccctgacgagcatcacaaaaatcgacgctcaagtcag<br>aggtggcgaaacccgacaggactataaagataccaggcgtttccccctggaagctccctcgtgcgctctcctgttccgaccctgccg<br>cttaccggatacctgtccgcctttctcccttcgggaagcgtggcgctttctcatagctcacgctgtaggtatctcagttcggtgtaggtcgtt<br>cgctccaagctgggctgtgtgcacgaaccccccgttcagcccgaccgctgcgccttatccggtaactatcgtcttgagtccaacccg<br>gtaagacacgacttatcgccactggcagcagccactggtaacaggattagcagagcgaggtatgtaggcggtgctacagagttctt<br>gaagtggtggcctaactacggctacactagaagaacagtatttggtatctgcgctctgctgaagccagttaccttcggaaaaagagtt<br>ggtagctcttgatccggcaaacaaaccaccgctggtagcggtggtttttttgtttgcaagcagcagattacgcgcagaaaaaaagga<br>tctcaagaagatcctttgatcttttctacggggtctgacgctcagtggaacgaaaactcacgttaagggattttggtcatgagattatcaa<br>aaaggatcttcacctagatccttttaaattaaaaatgaagttttaaatcaatctaaagtatatatgagtaaacttggtctgacagcggcc<br>gcaaatgctaaaccactgcagtggttaccagtgcttgatcagtgaggcaccgatctcagcgatctgcctatttcgttcgtccatagtgg<br>cctgactccccgtcgtgtagatcactacgattcgtgagggcttaccatcaggccccagcgcagcaatgatgccgcgagagccgcgt<br>tcaccggcccccgatttgtcagcaatgaaccagccagcagggagggccgagcgaagaagtggtcctgctactttgtccgcctccat<br>ccagtctatgagctgctgtcgtgatgctagagtaagaagttcgccagtgagtagtttccgaagagttgtggccattgctactggcatcgt<br>ggtatcacgctcgtcgttcggtatggcttcgttcaactctggttcccagcggtcaagccgggtcacatgatcacccatattatgaagaa<br>atgcagtcagctccttagggcctccgatcgttgtcagaagtaagttggccgcggtgttgtcgctcatggtaatggcagcactacacaat<br>tctcttaccgtcatgccatccgtaagatgcttttccgtgaccggcgagtactcaaccaagtcgttttgtgagtagtgtatacggcgacca<br>agctgctcttgcccggcgtctatacgggacaacaccgcgccacatagcagtactttgaaagtgctcatcatcgggaatcgttcttcgg<br>ggcggaaagactcaaggatcttgccgctattgagatccagttcgatatagcccactcttgcacccagttgatcttcagcatcttttactttc<br>accagcgtttcggggtgtgcaaaaacaggcaagcaaaatgccgcaaagaagggaatgagtgcgacacgaaaatgttggatgct<br>catactcgtcctttttcaatattattgaagcatttatcagggttactagtacgtctctcaaggataagtaagtaatattaaggtacgggagg<br>tattggacaggccgcaataaaatatctttattttcattacatctgtgtgttggttttttgtgtgaatcgatagtactaacatacgctctccatca<br>aaacaaaacgaaacaaaacaaactagcaaaataggctgtccccagtgcaagtgcaggtgccagaacatttctctggcctaactg<br>gcctcaat
**pJO-splice-ON**, positive control leading to constitutive eGFP expression in human cells	ggttatatagcataaatcaatattggctattggccattgcatacgttgtatctatatcataatatgtacatttatattggctcatgtccaatatg<br>accgccatgttggcattgattattgactagttattaatagtaatcaattacggggtcattagttcatagcccatatatggagttccgcgttac<br>ataacttacggtaaatggcccgcctggctgaccgcccaacgacccccgcccattgacgtcaataatgacgtatgttcccatagtaac<br>gccaatagggactttccattgacgtcaatgggtggagtatttacggtaaactgcccacttggcagtacatcaagtgtatcatatgccaa<br>gtccgccccctattgacgtcaatgacggtaaatggcccgcctggcattatgcccagtacatgaccttacgggactttcctacttggcag<br>tacatctacgtattagtcatcgctattaccatggtgatgcggttttggcagtacaccaatgggcgtggatagcggtttgactcacgggga<br>tttccaagtctccaccccattgacgtcaatgggagtttgttttggcaccaaaatcaacgggactttccaaaatgtcgtaataaccccgcc<br>ccgttgacgcaaatgggcggtaggcgtgtacggtgggaggtctatataagcagagctcgtttagtgaaccgtcagatcactagaag<br>ctttattgcggtagtttatcacagttaaattgctaacgcagtcagtgggcctcggcggccaagcttggcaatccggtactgttggtaaag<br>ccacctcgagctagcaccggtatggtgagcaagggcgaggagctgttcaccggggtggtgcccatcctggtcgagcgtgagtctat<br>gggacccttgatgttttctttccccttcttttctatggttaagttcatgtcataggaaggggagaagtaacagggtacagtttagaatggga<br>aacagacgaatgattgcatcagtgtggaagtctcaggatcgttttagtttcttttatttgctgttcataacaattgttttcttttgtttaattcttgct<br>ttcttttttttcttctccgcaatttttactattatacttaatgccttaacattgtgtataacaaaaggaaatatctctgagatacattaagtaactt<br>aaaaaaaaactttacacagtctgcctagtacattactatttggaatatatgtgtgcttatttgcatattcataatctccctactttattttctttat<br>ttttaattgatacataatcattatacattgctgtgccagcctttgcccagaggcgctctagctgggagcaaagtccggtcactgggcagc<br>accacccccggacttgcatgggtagccgctgagatggagcctgagcacacgcggccgctgttaacgcagtgtttctcttttttttcatc<br>aacaaccattgacgtattagcataggcgctctagctctgaaacaaccaaacaaccaaacaaccaaacaaccaaacaaccaaac<br>aaccaaacaaccaaacaaccaaacaaccaaacaaccaaacaaccaaacaacacaggttggtgctagctggccagtgagatc<br>caagaatcttcctgtctctgctgatccactgataggattacaagtacatgccaccaagcccagcttcctcttaccaggtgctggggacc<br>aaacttaggccctcattcctacacagtgaatacttatttatgggttaaagtgtaatgttttaatatgtgtacacatattgaccaaatcagggt<br>aattttgcatttgtaattttaaaaaatgctttcttcttttaatatactttttttgtttatcttatttctaatactttccctaatctctttctttcagggcaataa<br>tgatacaatgtatcatgcctctttgcaccattctaaagaataacagtgataatttctgggttaaggcaatagcaatatttctgcatataaat<br>atttctgcatataaattgtaactgatgtaagaggtttcatattgctaatagcagctacaatccagctaccattctgcttttattttatggttggg<br>ataaggctggattattctgagtccaagctaggcccttttgctaatcatgttcatacctcttatcttcctcccacagtggacggcgacgtaa<br>acggccacaagttcagcgtgtccggcgagggcgagggcgatgccacctacggcaagctgaccctgaagttcatctgcaccaccg<br>gcaagctgcccgtgccctggcccaccctcgtgaccaccctgacctacggcgtgcagtgcttcagccgctaccccgaccacatgaa<br>gcagcacgacttcttcaagtccgccatgcccgaaggctacgtccaggagcgcaccatcttcttcaaggacgacggcaactacaag<br>acccgcgccgaggtgaagttcgagggcgacaccctggtgaaccgcatcgagctgaagggcatcgacttcaaggaggacggca<br>acatcctggggcacaagctggagtacaactacaacagccacaacgtctatatcatggccgacaagcagaagaacggcatcaag<br>gtgaacttcaagatccgccacaacatcgaggacggcagcgtgcagctcgccgaccactaccagcagaacacccccatcggcga<br>cggccccgtgctgctgcccgacaaccactacctgagcacccagtccgccctgagcaaagaccccaacgagaagcgcgatcaca<br>tggtcctgctggagttcgtgaccgccgccgggatcactctcggcatggacgagctgtacaagcccaaaaaaaaacgaaaagtag<br>aggatccctgatctagagtcggggcggccggccgcttcgagcagacatgataagatacattgatgagtttggacaaaccacaacta<br>gaatgcagtgaaaaaaatgctttatttgtgaaatttgtgatgctattgctttatttgtaaccattataagctgcaataaacaagttaacaac<br>aacaattgcattcattttatgtttcaggttcagggggaggtgtgggaggttttttaaagcaagtaaaacctctacaaatgtggtaaaatcg<br>ataaggatccgtcgaccgatgcccttgagagccttcaacccagtcagctccttccggtgggcgcggggcatgactatcgtcgccgc<br>acttatgactgtcttctttatcatgcaactcgtaggacaggtgccggcagcgctcttccgcttcctcgctcactgactcgctgcgctcggtc<br>gttcggctgcggcgagcggtatcagctcactcaaaggcggtaatacggttatccacagaatcaggggataacgcaggaaagaac

	atgtgagcaaaaggccagcaaaaggccaggaaccgtaaaaaggccgcgttgctggcgtttttccataggctccgcccccctgacg<br>agcatcacaaaaatcgacgctcaagtcagaggtggcgaaacccgacaggactataaagataccaggcgtttccccctggaagct<br>ccctcgtgcgctctcctgttccgaccctgccgcttaccggatacctgtccgcctttctcccttcgggaagcgtggcgctttctcatagctca<br>cgctgtaggtatctcagttcggtgtaggtcgttcgctccaagctgggctgtgtgcacgaaccccccgttcagcccgaccgctgcgcctt<br>atccggtaactatcgtcttgagtccaacccggtaagacacgacttatcgccactggcagcagccactggtaacaggattagcagag<br>cgaggtatgtaggcggtgctacagagttcttgaagtggtggcctaactacggctacactagaagaacagtatttggtatctgcgctctg<br>ctgaagccagttaccttcggaaaaagagttggtagctcttgatccggcaaacaaaccaccgctggtagcggtggtttttttgtttgcaa<br>gcagcagattacgcgcagaaaaaaaggatctcaagaagatcctttgatcttttctacggggtctgacgctcagtggaacgaaaact<br>cacgttaagggattttggtcatgagattatcaaaaaggatcttcacctagatccttttaaattaaaaatgaagttttaaatcaatctaaagt<br>atatatgagtaaacttggtctgacagcggccgcaaatgctaaaccactgcagtggttaccagtgcttgatcagtgaggcaccgatctc<br>agcgatctgcctatttcgttcgtccatagtggcctgactccccgtcgtgtagatcactacgattcgtgagggcttaccatcaggccccag<br>cgcagcaatgatgccgcgagagccgcgttcaccggcccccgatttgtcagcaatgaaccagccagcagggagggccgagcga<br>agaagtggtcctgctactttgtccgcctccatccagtctatgagctgctgtcgtgatgctagagtaagaagttcgccagtgagtagtttcc<br>gaagagttgtggccattgctactggcatcgtggtatcacgctcgtcgttcggtatggcttcgttcaactctggttcccagcggtcaagcc<br>gggtcacatgatcacccatattatgaagaaatgcagtcagctccttagggcctcccgatcgttgtcagaagtaagttggccgcggtgttg<br>tcgctcatggtaatggcagcactacacaattctcttaccgtcatgccatccgtaagatgcttttccgtgaccggcgagtactcaaccaa<br>gtcgttttgtgagtagtgtatacggcgaccaagctgctcttgcccggcgtctatacgggacaacaccgcgccacatagcagtactttg<br>aaagtgctcatcatcgggaatcgttcttcggggcggaaagactcaaggatcttgccgctattgagatccagttcgatatagcccactct<br>tgcacccagttgatcttcagcatcttttactttcaccagcgtttcggggtgtgcaaaaacaggcaagcaaaatgccgcaaagaaggg<br>aatgagtgcgacacgaaaatgttggatgctcatactcgtcctttttcaatattattgaagcatttatcagggttactagtacgtctctcaag<br>gataagtaagtaatattaaggtacgggaggtattggacaggccgcaataaaatatctttattttcattacatctgtgtgttggttttttgtgtg<br>aatcgatagtactaacatacgctctccatcaaaacaaaacgaaacaaaacaaactagcaaaataggctgtccccagtgcaagtg<br>caggtgccagaacatttctctggcctaactggcctcaatattggccattagccatattattcatt
**pJO_splice-Dox-switch**,<br>plasmid containing the aptamer G12 for controlling mRNA processing of eGFP	tgtacatttatattggctcatgtccaatatgaccgccatgttggcattgattattgactagttattaatagtaatcaattacggggtcattagtt<br>catagcccatatatggagttccgcgttacataacttacggtaaatggcccgcctggctgaccgcccaacgacccccgcccattgacg<br>tcaataatgacgtatgttcccatagtaacgccaatagggactttccattgacgtcaatgggtggagtatttacggtaaactgcccacttg<br>gcagtacatcaagtgtatcatatgccaagtccgccccctattgacgtcaatgacggtaaatggcccgcctggcattatgcccagtaca<br>tgaccttacgggactttcctacttggcagtacatctacgtattagtcatcgctattaccatggtgatgcggttttggcagtacaccaatggg<br>cgtggatagcggtttgactcacggggatttccaagtctccaccccattgacgtcaatgggagtttgttttggcaccaaaatcaacggga<br>ctttccaaaatgtcgtaataaccccgccccgttgacgcaaatgggcggtaggcgtgtacggtgggaggtctatataagcagagctcg<br>tttagtgaaccgtcagatcactagaagctttattgcggtagtttatcacagttaaattgctaacgcagtcagtgggcctcggcggccaa<br>gcttggcaatccggtactgttggtaaagccacctcgagctagcaccggtatggtgagcaagggcgaggagctgttcaccggggtgg<br>tgcccatcctggtcgagcgtgagtctatgggacccttgatgttttctttccccttcttttctatggttaagttcatgtcataggaaggggagaa<br>gtaacagggtacagtttagaatgggaaacagacgaatgattgcatcagtgtggaagtctcaggatcgttttagtttcttttatttgctgttc<br>ataacaattgttttcttttgtttaattcttgctttctttttttttcttctccgcaatttttactattatacttaatgccttaacattgtgtataacaaaagga<br>aatatctctgagatacattaagtaacttaaaaaaaaactttacacagtctgcctagtacattactatttggaatatatgtgtgcttatttgca<br>tattcataatctccctactttattttcttttatttttaattgatacataatcattatacattgctgtgccagcctttgcccagaggcgctctagctgg<br>gagcaaagtccggtcactgggcagcaccacccccccggacttgcatgggtagccgctgagatggagcctgagcacacgcggccg<br>ctgttaacgcagtgtttctcttttttttcaggcgccggtcattgagtgaacagacgtcaagctagttgggctactggcttcggcgcctgaaa<br>caaccaaacaaccaaacaaccaaacaaccaaacaaccaaacaaccaaacaaccaaacaaccaaacaaccaaacaacca<br>aacaaccaaacaacacaggttggtgctagctggccagtgagatccaagaatcttcctgtctctgctgatccactgataggattacaa<br>gtacatgccaccaagcccagcttcctcttaccaggtgctggggaccaaacttaggccctcattcctacacagtgaatacttatttatgg<br>gttaaagtgtaatgttttaatatgtgtacacatattgaccaaatcagggtaattttgcatttgtaattttaaaaaatgctttcttcttttaatatact<br>tttttgtttatcttatttctaatactttccctaatctctttctttcagggcaataatgatacaatgtatcatgcctctttgcaccattctaaagaataa<br>cagtgataatttctgggttaaggcaatagcaatatttctgcatataaatatttctgcatataaattgtaactgatgtaagaggtttcatattgc<br>taatagcagctacaatccagctaccattctgcttttattttatggttgggataaggctggattattctgagtccaagctaggcccttttgcta<br>atcatgttcatacctcttatcttcctcccacagtggacggcgacgtaaacggccacaagttcagcgtgtccggcgagggcgagggcg<br>atgccacctacggcaagctgaccctgaagttcatctgcaccaccggcaagctgcccgtgccctggcccaccctcgtgaccaccctg<br>acctacggcgtgcagtgcttcagccgctaccccgaccacatgaagcagcacgacttcttcaagtccgccatgcccgaaggctacgt<br>ccaggagcgcaccatcttcttcaaggacgacggcaactacaagacccgcgccgaggtgaagttcgagggcgacaccctggtga<br>accgcatcgagctgaagggcatcgacttcaaggaggacggcaacatcctggggcacaagctggagtacaactacaacagcca<br>caacgtctatatcatggccgacaagcagaagaacggcatcaaggtgaacttcaagatccgccacaacatcgaggacggcagcg<br>tgcagctcgccgaccactaccagcagaacacccccatcggcgacggccccgtgctgctgcccgacaaccactacctgagcacc<br>cagtccgccctgagcaaagaccccaacgagaagcgcgatcacatggtcctgctggagttcgtgaccgccgccgggatcactctcg<br>gcatggacgagctgtacaagcccaaaaaaaaacgaaaagtagaggatccctgatctagagtcggggcggccggccgcttcgag<br>cagacatgataagatacattgatgagtttggacaaaccacaactagaatgcagtgaaaaaaatgctttatttgtgaaatttgtgatgct<br>attgctttatttgtaaccattataagctgcaataaacaagttaacaacaacaattgcattcattttatgtttcaggttcagggggaggtgtg<br>ggaggttttttaaagcaagtaaaacctctacaaatgtggtaaaatcgataaggatccgtcgaccgatgcccttgagagccttcaaccc<br>agtcagctccttccggtgggcgcggggcatgactatcgtcgccgcacttatgactgtcttctttatcatgcaactcgtaggacaggtgcc<br>ggcagcgctcttccgcttcctcgctcactgactcgctgcgctcggtcgttcggctgcggcgagcggtatcagctcactcaaaggcggt<br>aatacggttatccacagaatcaggggataacgcaggaaagaacatgtgagcaaaaggccagcaaaaggccaggaaccgtaa<br>aaaggccgcgttgctggcgtttttccataggctccgcccccctgacgagcatcacaaaaatcgacgctcaagtcagaggtggcgaa<br>acccgacaggactataaagataccaggcgtttccccctggaagctccctcgtgcgctctcctgttccgaccctgccgcttaccggata<br>cctgtccgcctttctcccttcgggaagcgtggcgctttctcatagctcacgctgtaggtatctcagttcggtgtaggtcgttcgctccaagc<br>tgggctgtgtgcacgaaccccccgttcagcccgaccgctgcgccttatccggtaactatcgtcttgagtccaacccggtaagacacg<br>acttatcgccactggcagcagccactggtaacaggattagcagagcgaggtatgtaggcggtgctacagagttcttgaagtggtgg<br>cctaactacggctacactagaagaacagtatttggtatctgcgctctgctgaagccagttaccttcggaaaaagagttggtagctcttg<br>atccggcaaacaaaccaccgctggtagcggtggtttttttgtttgcaagcagcagattacgcgcagaaaaaaaggatctcaagaag<br>atcctttgatcttttctacggggtctgacgctcagtggaacgaaaactcacgttaagggattttggtcatgagattatcaaaaaggatctt<br>cacctagatccttttaaattaaaaatgaagttttaaatcaatctaaagtatatatgagtaaacttggtctgacagcggccgcaaatgcta<br>aaccactgcagtggttaccagtgcttgatcagtgaggcaccgatctcagcgatctgcctatttcgttcgtccatagtggcctgactcccc<br>gtcgtgtagatcactacgattcgtgagggcttaccatcaggccccagcgcagcaatgatgccgcgagagccgcgttcaccggccc<br>ccgatttgtcagcaatgaaccagccagcagggagggccgagcgaagaagtggtcctgctactttgtccgcctccatccagtctatga<br>gctgctgtcgtgatgctagagtaagaagttcgccagtgagtagtttccgaagagttgtggccattgctactggcatcgtggtatcacgct

	cgtcgttcggtatggcttcgttcaactctggttcccagcggtcaagccgggtcacatgatcacccatattatgaagaaatgcagtcagc<br>tccttagggcctccgatcgttgtcagaagtaagttggccgcggtgttgtcgctcatggtaatggcagcactacacaattctcttaccgtc<br>atgccatccgtaagatgcttttccgtgaccggcgagtactcaaccaagtcgttttgtgagtagtgtatacggcgaccaagctgctcttgc<br>ccggcgtctatacgggacaacaccgcgccacatagcagtactttgaaagtgctcatcatcgggaatcgttcttcggggcggaaaga<br>ctcaaggatcttgccgctattgagatccagttcgatatagcccactcttgcacccagttgatcttcagcatcttttactttcaccagcgtttc<br>ggggtgtgcaaaaacaggcaagcaaaatgccgcaaagaagggaatgagtgcgacacgaaaatgttggatgctcatactcgtcct<br>ttttcaatattattgaagcatttatcagggttactagtacgtctctcaaggataagtaagtaatattaaggtacgggaggtattggacagg<br>ccgcaataaaatatctttattttcattacatctgtgtgttggtttttgtgtgaatcgatagtactaacatacgctctccatcaaaacaaaacg<br>aaacaaaacaaactagcaaaataggctgtccccagtgcaagtgcaggtgccagaacatttctctggcctaactggcctcaatattg<br>gccattagccatattattcattggttatatagcataaatcaatattggctattggccattgcatacgttgtatctatatcataata
