# Supplementary material for: Mechanism underlying the high regulatory performance of the doxycycline riboswitch G12
Source: Nat Commun. 2026 Aug 3;17:7697. doi: 10.1038/s41467-026-76256-2 (PMC13434769; doi:10.1038/s41467-026-76256-2)
Supplement: Supplementary file 2 [file 41467_2026_76256_MOESM2_ESM.pdf]

nature portfolio

Corresponding author(s): Beatrix Suess

Last updated by author(s): Jul 20, 2026

Reporting Summary

Nature Portfolio wishes to improve the reproducibility of the work that we publish. This form provides structure for consistency and transparency in reporting. For further information on Nature Portfolio policies, see our Editorial Policies and the Editorial Policy Checklist.

Statistics

For all statistical analyses, confirm that the following items are present in the figure legend, table legend, main text, or Methods section.

n/a	Confirmed	
☐	☒	The exact sample size (*n*) for each experimental group/condition, given as a discrete number and unit of measurement
☐	☒	A statement on whether measurements were taken from distinct samples or whether the same sample was measured repeatedly
☒	☐	The statistical test(s) used AND whether they are one- or two-sided<br>*Only common tests should be described solely by name; describe more complex techniques in the Methods section.*
☒	☐	A description of all covariates tested
☒	☐	A description of any assumptions or corrections, such as tests of normality and adjustment for multiple comparisons
☐	☒	A full description of the statistical parameters including central tendency (e.g. means) or other basic estimates (e.g. regression coefficient) AND variation (e.g. standard deviation) or associated estimates of uncertainty (e.g. confidence intervals)
☒	☐	For null hypothesis testing, the test statistic (e.g. *F*, *t*, *r*) with confidence intervals, effect sizes, degrees of freedom and *P* value noted<br>*Give P values as exact values whenever suitable.*
☒	☐	For Bayesian analysis, information on the choice of priors and Markov chain Monte Carlo settings
☒	☐	For hierarchical and complex designs, identification of the appropriate level for tests and full reporting of outcomes
☒	☐	Estimates of effect sizes (e.g. Cohen's *d*, Pearson's *r*), indicating how they were calculated

Our web collection on statistics for biologists contains articles on many of the points above.

Software and code

Policy information about availability of computer code

Data collection	Radioactivity was measured using a Tri-Carb 2800TR scintillation counter from PerkinElmer. Flow cytometry measurements were performed on a Beckman Coulter CytoFLEX S cytometer and analysed using the CytoFlow software (v1.1.1). Fluorescence-activated cell sorting was performed using a Beckman Coulter CytoFLEX SRT and analysed using the CytExpert SRT software (v1.0.2). Fluorescence and optical density were measured using a TECAN Infinite 200 Pro and data was analysed using the i-control software (Tecan, v2.0). SELEX pools were sequenced using NovaSeq (Illumina) via the sequencing service of Genewiz Azenta. Short-read sequencing was performed via the EZ-Amplicon sequencing of Genewiz Azenta. Fluorescnce titration was performed using a HORIBA Fluorolog FL3-22 and data was analysed using the FluorEsence software (v3.8.2.2). Isothermal titration calorimetry was perofrmed using a Malvern Instruments MicroCal PEAQ-ITC, data recorded using the MicroCal PEAQ-ITC software (v1.41). For single-molecule force spectroscopy a C-Trap (Lumicks) and the Bluelake software (v2.4.2) were used. Stopped flow spectroscopy was performed on a pi*-180 instrument (Applied Photophysics) and modelling analysis sone on DynaFit4 software.<br>The NGS data generated in this study have been deposited in the European Nucleotide Archive (ENA, ...) and together with cod eused for data analysis also on the Zenodo database under accession code 20424385 (https://zenodo.org/records/20424385) together with the code used for its analysis.
Data analysis	Cytometry data was analysed using the CytoFlow software (v1.1.1). Fluorescence-activated cell sorting data was analysed using the CytExpert SRT software (v1.0.2). data from the Tecan plate reader was analysed using the i-control software (Tecan, v2.0) and FlowJo (v10.10.1). Isothermal titration calorimetry was analysed using the MicroCal PEAQ-ITC analysis software (v1.1.0.1262). Analysis of stopped flow spectroscopy data was performed using DynaFit4. Single-molecule force spectroscopy data was analysed using the Igor Pro software from Wavemetrics (v8.0). The simulation sorce code is freely availabe under GNU public license, along with documentation, installation instructions and example data at https://github.com/lorenzo-rovigatti/oxDNA. Motif discovery from NGS data was performed using the MEME algorithm (MEME Suite v5.5.8). For creating the rooted tree diagram the stringdist package (v0.9.15) and the hclust function base R package (v4.5.1)

were used. Creation of the stacked bar plots was was done using ggplot2 (v4.0.0). SHAPE-MaP data was analysed using SHAPE-Mapper 2 (v2.1.3) from the Weeks lab, available with example data and instrcutions under https://github.com/Weeks-UNC/shapemapper2.

For manuscripts utilizing custom algorithms or software that are central to the research but not yet described in published literature, software must be made available to editors and reviewers. We strongly encourage code deposition in a community repository (e.g. GitHub). See the Nature Portfolio guidelines for submitting code & software for further information.

Data

Policy information about availability of data

All manuscripts must include a data availability statement. This statement should provide the following information, where applicable:

- Accession codes, unique identifiers, or web links for publicly available datasets
- A description of any restrictions on data availability
- For clinical datasets or third party data, please ensure that the statement adheres to our policy

All plasmids used in this publication are availbale upon request. NGS data and analysis are are publicly available under https://doi.org/10.5281/zenodo.20424385 (ZENODO).All experimental data are included in the Supplementary Information / Source Data file attached to the publicaiton.

Research involving human participants, their data, or biological material

Policy information about studies with human participants or human data. See also policy information about sex, gender (identity/presentation), and sexual orientation and race, ethnicity and racism.

Reporting on sex and gender	N/A
Reporting on race, ethnicity, or other socially relevant groupings	N/A
Population characteristics	N/A
Recruitment	N/A
Ethics oversight	N/A

Note that full information on the approval of the study protocol must also be provided in the manuscript.

Field-specific reporting

Please select the one below that is the best fit for your research. If you are not sure, read the appropriate sections before making your selection.

☒ Life sciences ☐ Behavioural & social sciences ☐ Ecological, evolutionary & environmental sciences

For a reference copy of the document with all sections, see nature.com/documents/nr-reporting-summary-flat.pdf

Life sciences study design

All studies must disclose on these points even when the disclosure is negative.

Sample size	No sample size calculation was performed. Sample size was determined based on pilot experiments and adjusted to match convetions in the field (Esvelt et al., Nature, 2011; Fishman et al., Nat. Biotechnol., 2024)
Data exclusions	No experimental data were excluded from analysis
Replication	All replications were succesful. The number of replicates is indicated for each experiment and figure.
Randomization	No randomization was used, as samples and controls were treated side-by-side using the identical protocols and workflows for analysis.
Blinding	No blinding was used, as the majority of data was automatically collected by machines and analyzed by standard workflows.

Reporting for specific materials, systems and methods

We require information from authors about some types of materials, experimental systems and methods used in many studies. Here, indicate whether each material, system or method listed is relevant to your study. If you are not sure if a list item applies to your research, read the appropriate section before selecting a response.

Materials & experimental systems

n/a	Involved in the study
☒	☐ Antibodies
☐	☒ Eukaryotic cell lines
☒	☐ Palaeontology and archaeology
☒	☐ Animals and other organisms
☒	☐ Clinical data
☒	☐ Dual use research of concern
☒	☐ Plants

Methods

n/a	Involved in the study
☒	☐ ChIP-seq
☐	☒ Flow cytometry
☒	☐ MRI-based neuroimaging

Eukaryotic cell lines

Policy information about cell lines and Sex and Gender in Research

Cell line source(s)
: Human HEK293 (ACC 305) and HeLa (ACC 57) cells were purchased from the DMSZ - German Collection of Microorganisms and Cell Cultures. Saccharomyces cerevisiae RS453alpha were kindly provided by the group of Norbert Sauer from the university of Erlangen.

Authentication
: The cells were not authenticated as they were recently obtained form the DMSZ.

Mycoplasma contamination
: All cells were tested negative using the MycoStrip test from InvivioGen.

Commonly misidentified lines (See ICLAC register)
: HEK293 were chosen for being well-suited for use in cytometry experiments.

Plants

Seed stocks
: N/A

Novel plant genotypes
: N/A

Authentication
: N/A

Flow Cytometry

Plots

Confirm that:

☒ The axis labels state the marker and fluorochrome used (e.g. CD4-FITC).

☒ The axis scales are clearly visible. Include numbers along axes only for bottom left plot of group (a 'group' is an analysis of identical markers).

☒ All plots are contour plots with outliers or pseudocolor plots.

☒ A numerical value for number of cells or percentage (with statistics) is provided.

Methodology

Sample preparation
: Yeast RS453alpha were cultured in 200 µL SCD-Ura medium in 96-deep-well round bottom plates (Greiner, cat. No. HR3-105) for 24h at 30°C shaking at 1200 rpm. 20 µL of this culture were transferred into 180 µL PBS in optical 96-well plates (Thermo FIsher Scientific, cat. No. 165305) and mixed before measurement.
Human HEK293 cells were seeded as 10x10^5 cells per well of a 24 well plate and cultured in DMEM at 37°C and 5 % CO_2.
The cells were transfected with the respective plasmids and 2 h post transfection the medium was changed to DMEM with or without doxycycline. The cells were incubated for 24 h. The cells were detached from the plate with trypsin/EDTA and transferred to PBS before being measured.

Instrument
: CytoFlex S & CytoFlex SRT (Beckman Coulter)

Software
: CytoFLow (v1.1.1)

Cell population abundance	N/A, since GFP and mCherry were measured from S. cerevisiae or HEK293 cell cultures.
Gating strategy	The general gating strategies are shown in Supplementary Figures S2a, S8 & S13.

☒ Tick this box to confirm that a figure exemplifying the gating strategy is provided in the Supplementary Information.
