# Supplementary material for: Mechanism underlying the high regulatory performance of the doxycycline riboswitch G12
Source: Nat Commun. 2026 Aug 3;17:7697. doi: 10.1038/s41467-026-76256-2 (PMC13434769; doi:10.1038/s41467-026-76256-2)
Supplement: Supplementary file 3 [file 41467_2026_76256_MOESM3_ESM.pdf]

Peer Review File

Mechanism underlying the high regulatory performance of the doxycycline riboswitch G12

Corresponding Author: Professor Beatrix Suess

This file contains all reviewer reports in order by version, followed by all author rebuttals in order by version.

Version 0:

Reviewer comments:

Reviewer #1

(Remarks to the Author)
Overall assessment

This manuscript describes the identification of a doxycycline-binding RNA aptamer (G12) using a selection strategy that integrates Capture-SELEX with intracellular screening, and the construction of doxycycline-responsive RNA switches that function in yeast and mammalian cells using distinct regulatory architectures. The study is supported by a substantial dataset spanning selection dynamics, in vitro binding measurements, cellular switching performance, and mechanistic characterization including single-molecule/kinetic analyses. Overall, the work is carefully executed and provides a strong foundation for a broadly useful tool.

To further strengthen the manuscript for Nature Communications and maximize its impact for a general readership, I recommend major revision with focused additions: (i) clearer conceptual positioning relative to prior riboswitch literature, (ii) more actionable guidance on when to use Capture-SELEX versus column-SELEX, and (iii) a small number of targeted experiments to reinforce usability in mammalian cells and ligand specificity.

Major revisions
1) Capture-SELEX vs column-SELEX: clearer “how-to” guidance for readers (highest priority)
The manuscript would be even more valuable if it provided explicit guidance on how readers should choose between Capture-SELEX and column-SELEX, and when running both in parallel is advisable. This is particularly relevant because, as presented, the highest-performing aptamer (G12) was recovered from the column-SELEX pool, while Capture-SELEX enriches different solutions under the docking/hybridization constraint.
· Add a concise workflow/decision framework (recommended library design, docking strategy, anticipated biases/failure modes, and practical decision points).
· Revise Fig. 1b to depict more explicitly how the selection/screening system relates to translation initiation in terms of RNA structure/accessibility, enabling readers to judge transferability to other translation-regulatory screens.
· If feasible, include a brief quantitative comparison such as functional hit rate (fraction of clones above a defined switching threshold). If replicate selections are beyond scope, state limitations clearly and adjust wording accordingly.
2) Mammalian relevance / usability: rationale for splicing readout + single-cell robustness
The mammalian validation is interesting, and clarifying the rationale for using a splicing-based readout (distinct from the yeast translation reporter) would help readers understand design choices and transferability. In addition, demonstrating robustness at the single-cell level would better establish usability for common workflows.
· Briefly explain why splicing was chosen in mammalian cells (e.g., architecture constraints, dynamic range, background, stability) and what aspects are expected to generalize across regulatory modes.
· Provide single-cell distribution-level evidence of switching (e.g., a fluorescent reporter with flow cytometry/FACS plots or an equivalent single-cell readout), demonstrating that ON/OFF states are practically separable rather than only detectable in population averages.
· In the Discussion, clarify which biological/medical use cases are realistically enabled by the current performance and what limitations remain for deployment in cell engineering (without necessarily adding a full application demonstration).
3) Ligand specificity and scope: strengthen selectivity while keeping experiments focused
The doxycycline-focused dataset is strong. Adding a small amount of additional selectivity evidence would further increase confidence in specificity and help avoid overgeneralization.

· Clarify whether any cellular cross-reactivity to tetracycline (or related analogs) is observed in yeast and/or mammalian assays, and reconcile this with the in vitro binding results (e.g., sensitivity limits, intracellular accumulation, indirect effects).
· Test a small panel of related analogs beyond tetracycline in the cellular assay (even limited data would be informative) to illustrate which chemical features drive specificity.
· If feasible, add tetracycline (or a close analog) to one selected mechanistic readout to help interpret ligand-specific conformational changes; if not feasible, briefly discuss this limitation.
· If the authors wish to emphasize broad platform generality beyond doxycycline, an additional selection example would be ideal; however, if this is beyond scope, temper generality claims and frame conclusions around the doxycycline case.

Minor revisions
4) Linking conformational dynamics to translation mechanism
Any biochemical probing of interplay with translation machinery (e.g., toeprinting or an in vitro translation assay) would strengthen biological interpretation. If beyond scope, a short discussion of alternative models and limitations would still be valuable.
5) Focused mutational validation across in vitro and cellular contexts
A focused set of mutations (plus compensatory/rescue where appropriate) tested both in vitro (structure/kinetics) and in cells (switch performance) would strengthen the causal link between the proposed structural model and switching activity.

Reviewer #2

(Remarks to the Author)
Overview
In this manuscript, Hoetzel et al. present a detailed mechanistic analysis of a doxycycline aptamer, “G12” that was discovered in conventional column SELEX. The mechanistic analysis includes functional validation of G12 as a ligand-dependent translational blockade in yeast, and decoding structural information relevant to ligand binding and a complex stabilization mechanism through a combination of methods including chemical probing and single-molecule force spectroscopy. G12 is the first Doxycycline aptamer described with high affinity and regulatory/riboswitch capabilities.
In addition to presenting the mechanistic studies and functional validation of G12, which are highly valuable to the field, a central claim of the manuscript is that RNA Capture-SELEX aids in the identification of highly functional aptamers. This claim derives from the nature of the Capture-SELEX experiment, which requires that aptamers candidates structurally rearrange to be selected, thus enriching for more dynamic aptamer mechanisms. While exciting if true, the evidence presented in the manuscript that supports this claim is weak for several reasons. First and foremost, while the manuscript showed that RNA Capture-SELEX clearly aids in the identification of moderate-performance aptamers, the exceptional G12 was identified only in the follow-up to conventional column SELEX, and it was shown that it cannot even hybridize to an RNA Capture-SELEX probe. In addition, there were no follow up experiments that compared the best aptamers selected by the two different approaches head-to-head.

Despite this, the manuscript provided strong and convincing evidence of the outstanding regulatory capacity of the G12 aptamer (and variants thereof). The demonstrated fold induction of translational blockade in yeast at 250µM doxycycline is clearly better than other aptamers tested for comparison, there is additional validation in HeLa cells, and the high affinity and specificity against tetracycline is impressive. The mutational studies and SHAPE-MaP data provide comprehensive insight into some of the structural behavior, showing what regions become less reactive after binding. The force spectroscopy data adds nuance to this structural data, for instance demonstrating clearly that there are two distinct kinetic paths to the bound state.

Overall, the paper is well-written and compelling. G12 is extremely well-characterized. The mechanistic findings are promising for the potential application of G12 in engineering applications, especially due to the exploration of multiple stem variants and function in multiple cell types. However, the emphasis of RNA Capture-SELEX feels out of place, which suggests some of the text, figures, and claims should be refined and reorganized. The generation and characterization of G12 is clearly a major step forward in riboswitch structure biology and engineering. Below we outline several comments that should be addressed prior to publication, noting that we did not focus on the single molecule studies as they are outside of our area of expertise.

Major Comments

Framing of the benefits of RNA Capture-SELEX:
The majority of the paper is dedicated to a characterization of an aptamer that was not discovered in the Capture-SELEX screening. As a result, the flow of the manuscript is hard to follow as presented, starting with the benefits of Capture-SELEX but pivoting into a focus solely on G12, which was not identified using Capture-SELEX. The benefits of Capture-SELEX seem relatively unexplored, and those benefits appear unrelated to the analysis of G12. It would be more interesting if the C01 aptamer from Capture-SELEX was compared head-to-head against other aptamers and/or previous doxycycline aptamers, alongside G12. Without additional data on variants from Capture-SELEX (as written), it would make more sense to reduce the scope of emphasis and claims around Capture-SELEX and instead focus on the characterization of G12. This could be done for example by moving some (or all) of Figures 1 and 2 to the supplementary information and re-organize figures around the characterization of G12.
Selection conditions

Figure 1d shows that during Column-SELEX the authors used selection, counter-selection and then a more stringent selection. However it appears that the same was not done for capture-SELEX. Why were the same selection conditions not

used in both cases?

Analysis of chemical probing data:
The chemical probing data presented in Figure 3e and f that shows ligand-dependent structural changes of G12 is on the surface very interesting and compelling. However, upon closer inspection of the underlying data in Figure S6 the data become less clear, raising several questions and necessitating some changes to how the data is presented.

In terms of data presentation: the reactivity plots in Figure S6a and c are cutoff at a reactivity of 4. This seems like an arbitrary cutoff and should not be used. If there is a data visualization issue then present the un-cutoff axes and show a zoom in of the data. In addition, the orange reactivity scale bars have no numbers on them, making it impossible to know what 'non-reactive' and 'reactive' actually mean – this should be corrected.

A question raised by the chemical probing data is why there are so many negative reactivities, which should not be possible and usually show up when there is poor data quality. Or at least, there appear to be negative reactivities: they could be large error bars that are hard to see in the current presentation.

With regards to the stabilization analysis, it appears that there are also positions that have negative stabilities which could be interesting and reflect positions that become more un-structured upon ligand binding. Rather than present stabilization on a scale of 0 to 1, the data should be presented with negative and positive stabilizations to reflect this. A common way to do this is to have a multi-colored scale where red is negative, blue is positive and white is neutral stabilization. In addition to presenting the stabilization bar chart, the authors should present side-by-side bar charts of the raw reactivities with and without ligand for key positions in the aptamer that change so that readers can compare these values and their associated errors. Finally, statistical analysis should be done to determine which position reactivity changes are actually statistically significant.

Comparison of G12 against other synthetic riboswitches:
The claim that G12 "outperforms all previously described synthetic riboswitches" is overstated, as the comparisons involve different aptamer-ligand pairs, which have distinct affinities and concentrations that affect apparent performance. It would be more accurate to emphasize that the riboswitch context in yeast and human cells enables strong regulatory behavior comparable to other synthetic riboswitches rather than implying general superiority across designs.

Minor Comments
Main Text
• Results, Paragraph 18 (Magnesium ions...): "50 µM Mg+2". The position of the charge is swapped.
• Discussion, Paragraph 4: The phrase "i.e. the aptamer" should be "i.e., the aptamer".
• Methods, Pool preparation vs. In vitro selection: Materials lists "32P- alpha -ATP" , but the In vitro selection method states that "32P-gamma-ATP" was used for body-labeling the transcribed RNA. Please verify which isotope was used.
• Methods, Control of mRNA processing in human cells: Luziferase should be Luciferase
• Methods, Off-rates and on-rates determination (should be bolded): Table S### reference should be specified.
Main Text Figures
• Figure 1 Legend (d): The antibiotic is spelled as "kanamycine A" , but in the main text Methods it is spelled "kanamycin A". Please ensure spelling consistency.
• Figure 1f: Consider adding an arrow to point out C01 (top of the Capture SELEX box plots)
• Figure 2h: Consider adding fold change per construct as a separate graph or annotation.
• Figure 3 Legend (a): There is a typo in the color coding description: "variabel position" should be "variable position."
• Figure 3i: Place the complete DNA template at the top of the schematic, with the ON and OFF states depicted as branches below.
• Figure 4: it is unclear if the coloration of 4e is meant to match the coloration of 4g. Please disambiguate the color scheme.
References
• Misspelling: "Doxycyline"
Supplement
• Figure S3 Legend (c): There is a typo where "re arrow heads" should be corrected to "red arrow heads".
• Figure S4 Legend: The lettering for the S1 variant restarts at "d)", "e)", "f)" after already using "d)", "e)", "f)" for the M3 variant. This should be updated sequentially to "g)", "h)", "i)".
• Figure S5 Legend: The lettering is highly inconsistent and missing parentheses. It lists "c)" , "d)" , then "e Titration curve" , then repeats "d) Fluorescence signal" , and jumps to "g)".
• Figure S10 / S11 Legend: Figure S11 is labled S10.
• Figure S11 Legend (d): The sentence ends abruptly with "fit according to equation" and provides no actual equation.
• Supplementary Methods, Kinetic modelling: The capitalization in the phrase "It Is Important" should be corrected to "It is important".
• Supplementary Methods, Kinetic modelling: The software is referred to as "Dynafit4", but it is capitalized as "DynaFit4" in the main text and DYNAFIT in references. Ensure consistent capitalization across both documents.

Reviewer #3

(Remarks to the Author)
I co-reviewed this manuscript with one of the reviewers who provided the listed reports. This is part of the Nature Communications initiative to facilitate training in peer review and to provide appropriate recognition for Early Career

Researchers who co-review manuscripts.

Reviewer #4

(Remarks to the Author)
I co-reviewed this manuscript with one of the reviewers who provided the listed reports. This is part of the Nature Communications initiative to facilitate training in peer review and to provide appropriate recognition for Early Career Researchers who co-review manuscripts.

Version 1:

Reviewer comments:

Reviewer #1

(Remarks to the Author)
The authors have addressed my major concerns in a substantial and constructive manner. The revised manuscript includes additional experimental validation, including single-cell analysis of mammalian switching, expanded ligand-selectivity assays, improved SHAPE-MaP data, and additional mutational/binding analyses. Overall, I find the experimental response thorough and the revised manuscript significantly strengthened.

I remain somewhat cautious about the breadth of the conceptual claims regarding Capture-SELEX, particularly because the best-performing aptamer, G12, was recovered from the column-SELEX pool rather than the Capture-SELEX pool. Nevertheless, the authors now discuss the limitations more clearly, and I agree that the main strength of the manuscript lies in the discovery and detailed mechanistic characterization of G12 as a high-performance doxycycline-responsive RNA regulator.

I also reviewed the revised Supplementary Information. The added comparison between column-SELEX and Capture-SELEX is useful and provides a promising practical resource for readers, especially the workflow, anticipated biases, and decision-making framework. I support retaining this information in the Supplementary Information, provided that the associated claims in the main text remain appropriately tempered. However, the Supplementary Information still appears to contain several editorial issues that should be corrected before publication, including apparent residual author/editorial comments or unpolished text, figure-numbering inconsistencies, table-numbering inconsistencies, and minor typographical errors. These issues do not affect my overall assessment of the science, but they should be carefully removed or corrected in the final version.

I therefore support publication after minor textual and editorial revisions. I have the following remaining comments, which should be addressable by brief clarifications and careful final editing:

1. Rationale for the mammalian splicing readout
The rebuttal explains that the yeast translation roadblock mechanism does not readily transfer to mammalian cells, likely due to stronger helicase/scanning activity of mammalian ribosomes, and that splicing control was therefore chosen as a more robust mammalian regulatory architecture. This is a useful and important rationale, but it is only weakly conveyed in the manuscript ("We decided to engineer a splice-regulating riboswitch, based on its reliable regulatory performance in human cells across target genes and cell types."). I suggest adding one or two sentences in the Results or Discussion to explicitly explain why the authors chose a splicing-based mammalian readout rather than attempting the same translation-roadblock architecture used in yeast.

2. Tempering biomedical and cell-engineering claims
G12 is clearly a promising regulatory module, and the added mammalian data strengthen its potential utility. However, the current mammalian validation is still based mainly on reporter/splicing assays, transient transfection, and relatively high doxycycline concentrations. Thus, statements regarding biomedical applications, therapeutic cell engineering, or broad deployment should remain appropriately cautious. I recommend using language such as “potential,” “may enable,” or “could be useful for future applications,” rather than implying that these applications have already been demonstrated.

3. Final clean-up of the Supplementary Information
The new supplementary section comparing column-SELEX and Capture-SELEX is helpful and should be retained. However, the Supplementary Information should be carefully checked before publication to remove any residual author/editorial comments or unpolished text and to correct remaining figure- and table-numbering inconsistencies and typographical errors.

Provided these textual and editorial clarifications are incorporated, I do not think further experiments are necessary. The revised manuscript is suitable for publication.

Reviewer #2

(Remarks to the Author)
The response from the authors was thorough and addressed all my concerns. The added elements strengthen the manuscript. I have no further comments.

Reviewer #3

(Remarks to the Author)
I co-reviewed this manuscript with one of the reviewers who provided the listed reports. This is part of the Nature Communications initiative to facilitate training in peer review and to provide appropriate recognition for Early Career Researchers who co-review manuscripts.

Reviewer #4

(Remarks to the Author)
I co-reviewed this manuscript with one of the reviewers who provided the listed reports. This is part of the Nature Communications initiative to facilitate training in peer review and to provide appropriate recognition for Early Career Researchers who co-review manuscripts.

Open Access This Peer Review File is licensed under a Creative Commons Attribution 4.0 International License, which permits use, sharing, adaptation, distribution and reproduction in any medium or format, as long as you give appropriate credit to the original author(s) and the source, provide a link to the Creative Commons license, and indicate if changes were made.
In cases where reviewers are anonymous, credit should be given to 'Anonymous Referee' and the source.
The images or other third party material in this Peer Review File are included in the article's Creative Commons license, unless indicated otherwise in a credit line to the material. If material is not included in the article's Creative Commons license and your intended use is not permitted by statutory regulation or exceeds the permitted use, you will need to obtain permission directly from the copyright holder.
To view a copy of this license, visit https://creativecommons.org/licenses/by/4.0/
